# MEMS inductor fabrication and emerging applications in power electronics and neurotechnologies

**DOI:** 10.1038/s41378-021-00275-w

**Published:** 2021-08-11

**Authors:** Hoa Thanh Le, Rubaiyet I. Haque, Ziwei Ouyang, Seung Woo Lee, Shelley I. Fried, Ding Zhao, Min Qiu, Anpan Han

**Affiliations:** 1 The Rowland Institute at Harvard, Harvard University, Cambridge, MA USA; 2 Department of Mechanical Engineering, Technical University of Denmark, Lyngby, Denmark; 3 Department of Electrical Engineering, Technical University of Denmark, Lyngby, Denmark; 4 Department of Neurosurgery, Massachusetts General Hospital, Harvard Medical School, Boston, MA USA; 5 Boston VA Healthcare System, Boston, MA USA; 6 Key Laboratory of 3D Micro/Nano Fabrication and Characterization of Zhejiang Province, School of Engineering, Westlake University, Hangzhou, China; 7 Institute of Advanced Technology, Westlake Institute for Advanced Study, Hangzhou, China

**Keywords:** Engineering, Nanoscience and technology

## Abstract

MEMS inductors are used in a wide range of applications in micro- and nanotechnology, including RF MEMS, sensors, power electronics, and Bio-MEMS. Fabrication technologies set the boundary conditions for inductor design and their electrical and mechanical performance. This review provides a comprehensive overview of state-of-the-art MEMS technologies for inductor fabrication, presents recent advances in 3D additive fabrication technologies, and discusses the challenges and opportunities of MEMS inductors for two emerging applications, namely, integrated power electronics and neurotechnologies. Among the four top-down MEMS fabrication approaches, 3D surface micromachining and through-substrate-via (TSV) fabrication technology have been intensively studied to fabricate 3D inductors such as solenoid and toroid in-substrate TSV inductors. While 3D inductors are preferred for their high-quality factor, high power density, and low parasitic capacitance, in-substrate TSV inductors offer an additional unique advantage for 3D system integration and efficient thermal dissipation. These features make in-substrate TSV inductors promising to achieve the ultimate goal of monolithically integrated power converters. From another perspective, 3D bottom-up additive techniques such as ice lithography have great potential for fabricating inductors with geometries and specifications that are very challenging to achieve with established MEMS technologies. Finally, we discuss inspiring and emerging research opportunities for MEMS inductors.

## Introduction

Transistors, capacitors, resistors, and inductors are the four fundamental components of electrical circuits. The importance of each component to science, engineering and society is paramount. In this review, we focus on inductors such as solenoid, toroidal, and spiral inductors, coils, and transformers. For the first time, we review microelectromechanical systems (MEMS) inductor fabrication technologies, which serve as the foundation of our literature search. We analyze and process the literature to give the reader a comprehensive overview of MEMS inductor fabrication technologies and emerging applications. In “MEMS fabrication of inductors” section, we group MEMS inductors into four categories and present the technical terminology used for MEMS inductor fabrication. In the same section, we review four different fabrication technologies and highlight their advantages for their target applications.

Most reported microinductors were made with established top-down or subtractive MEMS microfabrication and nanofabrication methods such as lithography, etching, electroplating, and thin-film deposition processes. Owing to important and creative MEMS fabrication technology innovations, additive microfabrication, and nanofabrication methods have emerged over the past 10 years. In “Emerging 3D nanofabrication technologies” section, we review important emerging nanoscale fabrication methods that have been used for MEMS inductor fabrication or hold promise for becoming new versatile tools for microinductor and nanoinductor production. An overview section maps the emerging technologies, and four promising technologies are presented.

**Fig. 1:**
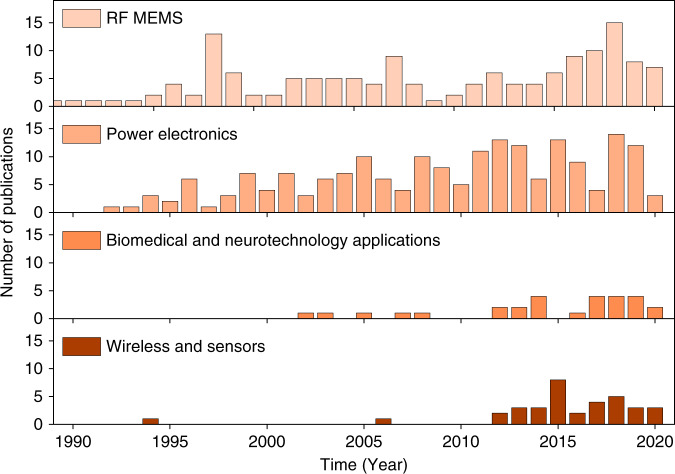
Number of publications on MEMS inductor applications in the last 30 years. Please see supplementary material for source data

In addition to reviewing inductor fabrication technologies, our literature study and analysis categorize MEMS inductor applications into four groups: radio frequency (RF) MEMS, wireless and sensors, power electronics, and biomedical MEMS (Fig. 1). RF MEMS and power electronics applications of MEMS inductors account for over 300 studies. Excellent reviews on RF applications exist^1,2^. However, there are no reviews on the three other applications. Thus, we reasoned that it is important to review the power electronics applications, as presented in “Power electronics applications” section. The number of studies on power inductors for power converters has increased steadily following the rapid growth of exciting smart electronic devices, wearables, light-emitting diode (LED) lighting, and the Internet of things (IoT).

In the last 10 years, the usage of MEMS inductors for biomedical applications has been explored, and we observed an increasing number of publications. In “Biomedical and neurotechnology applications” section, we review a subset of the biomedical applications, which is an emerging field of neurotechnology. Neurotechnology research is substantially supported by the BRAIN Initiative, which focuses on the development of cutting-edge neurotechnologies for science and medicine. Here, we review electrifying new developments in magnetic stimulation of the brain using MEMS coils, which might outperform the well-established electrical stimulation. We discuss important challenges and research opportunities addressing societal challenges.

## MEMS fabrication of inductors

### Overview

**Fig. 2:**
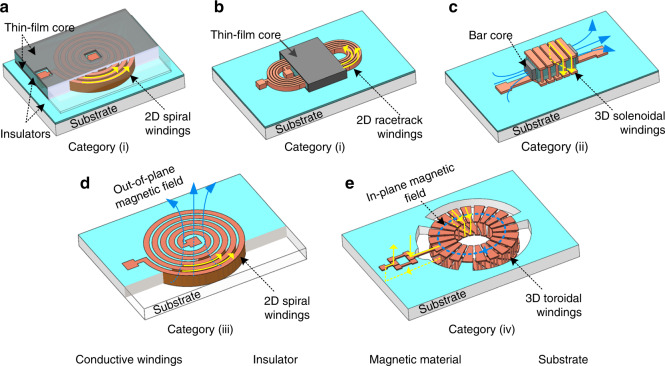
3D illustrations of four categories of MEMS inductor. Category (i): 2D on-substrate inductors such as spiral inductors (**a**) and racetrack inductors (**b**). Category (ii): 3D on-substrate solenoidal inductors with a magnetic bar core (**c**). Category (iii): 2D in-substrate spiral inductor (**d**). Category (iv): 3D in-substrate toroidal inductor (**e**). The directions of current flows are depicted by the yellow arrows

MEMS inductors consist of three parts: (i) conductive windings, (ii) insulators, and (iii) a core. First, conductive windings are used to carry currents, thus producing magnetic flux. They are made of electrically conductive metals, e.g., copper (Cu), and are fabricated by deposition techniques such as electrodeposition or sputtering. Second, insulators are used to provide electrical isolation between adjacent conductor windings and between windings and the underlying substrate. Here, insulators are dielectric thin films such as silicon oxide (SiO_2_), aluminum oxide (Al_2_O_3_), or silicon nitride (SiN). The selection of dielectric materials and deposition techniques is performed carefully based on the required isolation level and processing temperature. Third, MEMS inductor cores refer to the material in the magnetic flux path. Inductor cores can be made of either nonmagnetic materials such as air (Fig. 2d, e) or magnetic materials that come in a variety of shapes, such as magnetic thin films (Fig. 2a, b) and bar shapes (Fig. 2c). Innovatively, magnetic cores are also placed around the windings.

MEMS inductors are categorized into four groups based on the shape of the conductor windings and their position relative to the substrate: (i) 2D on-substrate inductors, (ii) 3D on-substrate inductors, (iii) 2D in-substrate inductors, and (iv) 3D in-substrate inductors. Figure 2 shows 3D illustrations of the MEMS inductors of these four groups, including 2D on-substrate spiral and racetrack inductors (Fig. 2a, b), a 3D on-substrate solenoidal inductor (Fig. 2c), a 2D in-substrate spiral inductor (Fig. 2d), and a 3D in-substrate toroidal inductor (Fig. 2e). Table 1 summarizes these fabrication technologies, applications, merits, and limitations corresponding to inductors in these four inductor groups.

**Table 1:** MEMS inductor fabrication technology

Fabrication technology	Geometry	Year	Key features	Application	Developments and trends
2D surface micromachining	Planar spiral		On-chip square spiral.	RF	Winding layout optimization^152^, winding density improvement, substrate isolation by suspended windings^17,25^, and buried oxide^14,15^.
	Racetrack	2012^28,153^	Substrate-embedded racetrack windings, nanolaminated sputtered Co-Zr-O magnetic core.	Power	Winding density improvement by high-aspect-ratio (high-AR) winding^10–12^, thin-film nanolaminated magnetic core^27,154^, axial anisotropy implementation^26,155^.
3D surface micromachining	Solenoid	2005^156^	Polymer-core windings, high profile.	Power	Nanolaminated core implementation^38^.
	Solenoid	2008^36^	Low profile, CMOS-compatible integration.	Power	Substrate electrical isolation by suspended windings^34,35^, substrate-embedded windings^157,158^, thin-film nanolaminated magnetic core^37,38^, low-loss magnetic materials^26,38,101,102^, vertical laminated magnetic core^158^.
	Toroidal	2013^159^	Polymer-core windings, high profile, nanolaminated core.		Substrate-embedded windings^157^, radical anisotropy implementation for toroidal inductor^160^.
TSV fabrication processes	Spiral	2011^51^	Low profile, substrate-embedded spiral windings, Si core, through-substrate interconnections.	Power, wireless	Through-silicon windings, double-sided embedded windings^161^, stacked substrate^162^, magnetic core integration.
	Solenoid, Toroidal	2018^57^	Void-free high-AR TSVs, substrate-isolation by suspended windings.	Power	Hollow Cu TSVs^45,163^, magnetic self-assembly for TSV fabrication^164^, void-free high-AR TSV filling^165,166^, magnetic core integration^59,106,163^. Substrate materials: glass^45^, organic substrate^163^, stacked substrate^45,167,168^.
Other processes	Solenoid	2019^84^	3D-printed vertical solenoid, selective silver deposition.	RF	Direct writing by femtosecond laser^169^.
	3D spiral	2015^63^	Self-rolled-up membrane.	RF, BioMEMS	Magnetic core integration using magnetic liquid^64^, multilayer transformers^170^.
	Solenoid	2010^68^	Wire-bond vertical solenoid, automatic wire bonder.	RF, sensing	Horizontal toroidal winding^69^, soft ferrite core integration^69^.

The four inductor categories are realized by different fabrication technologies, which play an essential role in MEMS inductor technology development. They determine an inductor’s physical dimensions and construction materials; therefore, the fabrication process greatly influences the inductor properties. The generic properties of MEMS inductors are the quality factor, inductance density, and operating frequency. Furthermore, there are important parameters for MEMS inductors, such as the EMI, breakdown voltage, leakage current, and parasitic capacitance.

**Fig. 3:**
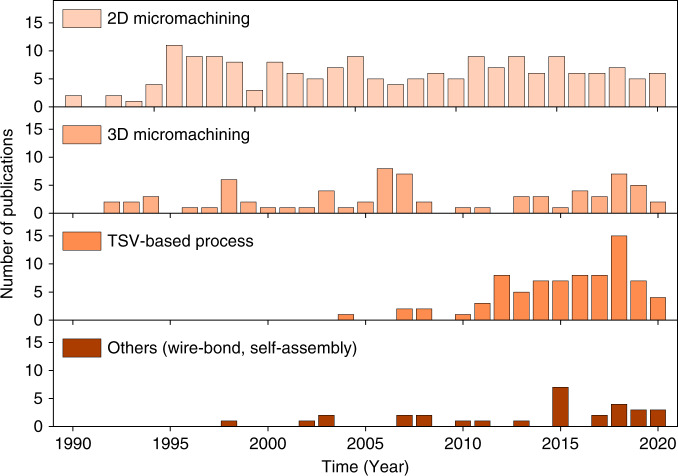
Number of publications on MEMS inductor fabrication technologies over time. Please see supplementary material for source data

We classify inductor fabrication technologies into four categories: 2D micromachining, 3D micromachining, through-substrate via (TSV)-based technology, and other technologies such as wire bonding, 3D printing, and self-assembly approaches. Figure 3 summarizes the development of MEMS inductor fabrication technologies since 1990.

2D and 3D micromachining technologies were used in the early 1990s and have been studied intensively since then. In the last 10 years, TSV technology has received tremendous attention and has become an emerging technology for 3D MEMS inductor fabrication and integration. Advances in TSV technology have enabled a new category of compact, robust, high-quality TSV inductors that find themselves particularly useful in power conversion applications. Wire bonding, stress-activated self-assembly processes, and 3D printing have also been reported.

Next, we review these four fabrication approaches and conclude with our view on future development trends of MEMS inductor fabrication technology.

### 2D surface micromachining

**Fig. 4:**
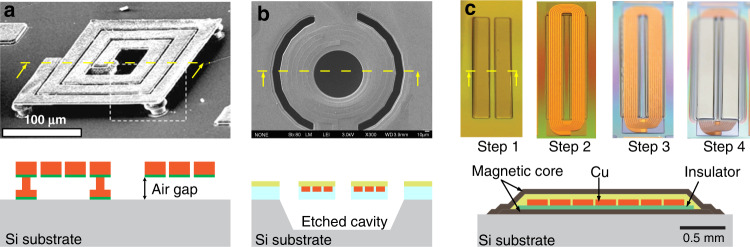
2D micromachined spiral inductors. Suspended windings and air gaps made by **a** removing a sacrificial layer^25^ (Copyright IEEE 2004) and **b** etching the underlying substrate^17^ (Copyright MDPI 2011). **c** 2D micromachined racetrack inductor with a magnetic thin-film core^27^ (Copyright IEEE 2013)

The first fabrication technology for MEMS inductor fabrication is 2D surface micromachining (Fig. 4). The development of surface micromachined inductors dates back to the early 1990s, with a number of foundational studies by Allen et al.^3,4^ on meander magnetic core inductors, Sullivan et al.^5^ on racetrack inductors, and Sato et al.^6^ on strip line inductors. They are widely used in many applications because of their compact size, process simplicity, and CMOS compatibility. Tremendous efforts have been invested towards design optimization^7^ and building physical models for 2D planar inductors^8,9^, providing a comprehensive understanding of how physical parameters and high-frequency eddy currents affect inductor performance. Here, we focus on fabrication advances in winding deposition technology and methods to enhance the electrical isolation between a winding and the substrate to minimize unwanted parasitic capacitance and substrate losses.

First, developments in winding technology have been driven towards the creation of compact, thick, high-aspect-ratio windings. Resist mold electrodeposition is a powerful method for fabricating 2D planar inductor windings; thus, high-aspect-ratio lithography processes are necessary. Anthony et al.^10^ studied high-aspect-ratio THB-151N resist molds for Cu electrodeposition. An aspect ratio of 1:17 was achieved, resulting in the successful fabrication of 80-µm-thick, 25-µm-wide, and 5-µm-spacing spiral inductor windings. They showed that the direct current (DC) resistance of 80-µm-thick winding inductors is 42% lower than that of 50-µm-thick winding inductors. In addition, inductors with a 5-µm spacing also have 25% smaller footprints than inductors with a 15-µm spacing. Similar studies on other high-aspect-ratio resist molds can also be found^11,12^.

Second, another notable improvement in the 2D micromachining process is the fabrication approaches followed to increase winding-substrate isolation to reduce unwanted parasitic capacitance and substrate losses^13^. There are two approaches. The first approach is to fabricate inductors on a thick isolation layer. A common method to fabricate thick buried oxide islands is to etch deep trenches into Si substrates followed by a SiO_2_ deposition step either by PECVD^14,15^ or thermal oxidation^16^. For example, the quality factor increased from 3.5 at 4.6 GHz to 7 at 7 GHz for inductors fabricated on 2-µm-thick and 20-µm-thick oxide layers, respectively^16^. The second approach is to induce an air gap between the winding conductors and substrate, resulting in suspended windings^17–24^. This is carried out by removing materials under the winding conductors, for example, by using sacrificial layers^25^ or by etching the substrate under the inductor windings^18^. In general, the quality factor and operating frequency increase as a result.

Another approach is to integrate magnetic thin films that act as a shield to reduce eddy currents generated in the underlying substrate. In addition, a magnetic thin-film core is a key feature to achieve a high inductance density, which is desired in applications such as switched-mode power supplies (SMPSs)^6^. A similar design of windings and thin-film cores can be seen in racetrack inductors^26^.

Figure 4c shows an elegant fabrication process of the racetrack inductor^27^. The process has four steps: (i) depositing the bottom magnetic thin film, (ii) depositing the 1st insulator layer and Cu windings, (iii) depositing the 2nd insulator layer, and (vi) depositing the top magnetic thin film. Similar fabrication approaches can be found for other racetrack power inductors^28,29^. Although the winding structures are similar in most racetrack inductors, innovative variations exist in magnetic thin-film technology. For example, because racetrack inductors are mainly used for power conversion, developments have focused on discovering new magnetic materials and new core structures for high permeability and low core losses. Recent developments in magnetic materials are summarized in references^30,31^.

### 3D surface micromachining

**Fig. 5:**
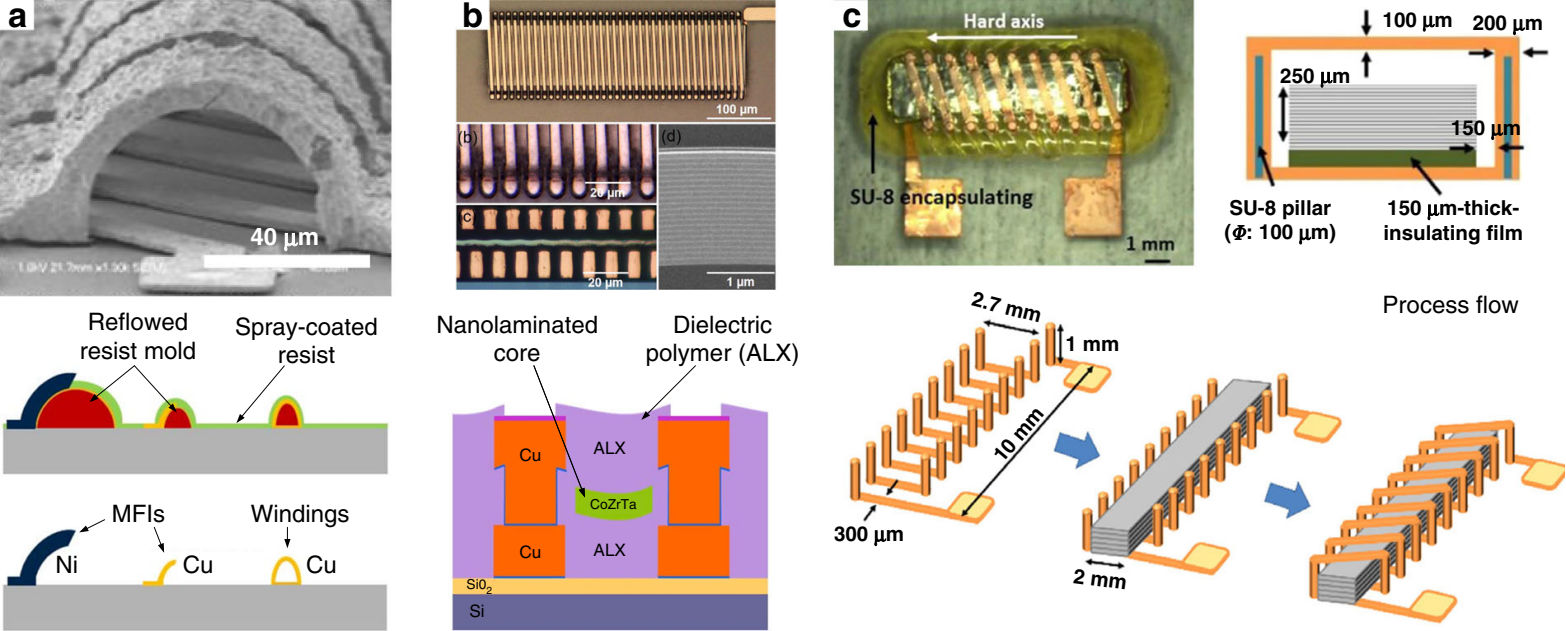
3D on-substrate inductors. **a** 3D air-core solenoidal inductor with dome-shaped windings^32^ (Copyright IEEE 2018). **b** 3D magnetic-core solenoidal inductor with a profile of 150 µm^37^ (Copyright IEEE 2019). **c** 3D magnetic-core solenoidal inductor with a tall profile of 1 mm, fabricated by a high-aspect-ratio SU-8 process for vertical windings^38^ (Copyright IOP 2015)

The second fabrication technology is 3D surface micromachining technology, which has been developed for fabricating 3D inductors such as solenoid and toroidal inductors. There are three groups of on-substrate 3D micromachined inductors: low-profile air-core inductors (Fig. 5a), low-profile magnetic-core inductors (Fig. 5b), and tall-profile inductors (Fig. 5c).

3D micromachined processes for low-profile inductors typically involve several resist-mold electroplating and sacrificial layers to construct multiple levels of conductors. Zia et al.^32^ reported a 110-µm-tall domed-shaped solenoidal inductor for RF applications, as shown in Fig. 5a. The key aspect is the 3D patterning of Cu windings on dome-shaped resist molds created by resist reflowing. The fabricated 110-µm-tall inductor has an inductance of 2.5 nH with a quality factor of 25 at 33 GHz. Small-inductance and low-profile 3D micromachined air-core solenoidal inductors are used for RF applications^30,33^. In some cases^34,35^, suspending inductor windings improves the high-frequency quality factor and frequency of 3D micromachined solenoidal inductors. The underlying substrate material is etched to produce an air gap between the windings and substrate, thus reducing unwanted parasitic capacitance and substrate effects. A similar approach is also found in the 2D micromachining processing of 2D inductors, as mentioned above.

For high-frequency power conversion, a higher inductance density is needed. Two approaches exist: either integrate the magnetic core between the 3D windings^36,37^ or increase the inductor height, because the inductance of the solenoidal and toroidal inductors is proportional to the core cross-sectional area. For the first approach, an example of a low-profile 3D magnetic-core inductor is shown in Fig. 5b. Michael et al.^37^ reported 150-µm-tall solenoid inductors embedded with a sputtered nanolaminated magnetic core for high-frequency power conversion. An inductance of up to 500 nH was achieved with a stack of 20 layers alternating between an 80-nm-thick Co–Zr–Ta magnetic layer and a 4-nm-thick Al_2_O_3_ layer. The second approach is to increase the inductor cross-sectional area. For example, SU-8 technology was utilized to fabricate 3D inductors with mm-tall, high-aspect-ratio vertical conductors^38,39^. In these studies, polymer-encapsulated vertical pillars were used as supports for subsequent Cu electroplating to form inductor vertical conductors. Figure 5c illustrates a process reported by Kim et al.^38^, which starts with the fabrication of SU-8 support pillars followed by seed layer sputtering and Cu electroplating to form the bottom and vertical windings. Next, a premade laminated magnetic core is inserted on an insulating film between vertical solenoidal windings. SU-8 pellets are then cast on a substrate to partially cover the Cu windings and magnetic core. Last, an electroplating mold is lithographically patterned, followed by top Cu winding electrodeposition. On-chip solenoid inductors of 1-mm-tall, 200-µm-diameter vertical vias were successfully demonstrated. Because of the mm-tall profile, these inductors are suitable for high-power applications where the device profile is not a limiting factor. Alternatively, windings can be embedded into the substrate to construct tall 3D inductors for high inductance.

### Through-substrate-via fabrication process

TSV technology was originally developed as a packaging solution for the vertical integration of multiple chips in the third dimension^40,41^. This packaging approach uses chips, or so-called interposers, consisting of vertical interconnections and redistribution layers (RDLs) on both substrate sides to pass electrical signals between stacked chips, thus substantially increasing the interconnection density. TSV technology was adapted for MEMS inductor fabrication in the mid-2000s, with studies on both 2D spiral inductors^42–44^ and 3D solenoid inductors^45^.

In the last 10 years, substantial developments have been made in all four aspects of TSV fabrication technology, including (i) TSV hole etching, (ii) dielectric insulator deposition, (iii) TSV conductor deposition, and (iv) substrate planarization and routing conductor patterning.

First, TSV hole fabrication technologies can be categorized into four main methods: physical ablation (laser drilling and sand blasting), lithographically based processes (photosensitive glass substrate^46^), wet etching (metal-assisted wet etching^47^), and plasma etching (deep reactive ion etching (DRIE) Bosch Si etching, SiO_2_ dry etching). Details on the advantages and disadvantages of each method are presented in reference^48^. According to reference^48^, DRIE Si etching using the Bosch process is by far the most versatile technique, providing excellent control over TSV dimensions, e.g., fine TSV spacings (10–15 µm) and ultrahigh aspect ratios (AR = 50–100).

The second step is dielectric insulator deposition. The dielectric constant, thickness, and conformality are important properties that have a large influence on the parasitic capacitance, breakdown voltage, and cross-talk between TSVs. The insulator deposition technology needs to be carefully considered. Deposition technologies include plasma-enhanced vapor deposition (PECVD) SiO_2_, atomic layer deposition (ALD) SiO_2_ and Al_2_O_3_, and polymers such as SU-8, spin-coated or PVD-growth polyimide (PI), polybenzoxazole (PBO), or benzocyclobutene (BCB). A summary of important characteristics such as the conformality, processing temperature, and dielectric properties of these materials and the corresponding deposition technologies are presented in reference^48^.

The third step is vertical via conductor fabrication. Electrodeposition is a widely used technology for TSV fabrication, in addition to other methods such as the magnetic assembly of microwires^49^. There are two approaches for TSV electrodeposition: electroless electroplating for hollow TSVs, top-down electroplating for half-through TSVs, and bottom-up electroplating for TSVs^50^. Electroless plating offers great conformality, but it is time-consuming and requires a conformally coated seed layer on the TSV sidewalls. It is often used for fabricating hollow TSVs. Top-down electroplating is the most developed technique for TSV fabrication. It is most suited for fabricating 2D in-substrate inductors^51–56^. To realize TSVs, extra steps of substrate back-lapping and planarization are needed. Bottom-up electroplating enables the fabrication of tall, high-aspect-ratio, solid TSVs, at the cost of a long plating time. This technique is best used in fabricating inductors with thick windings, as required to handle high DC and AC currents, such as in power electronic applications. For example, Wang et al.^43^ demonstrated TSV spiral inductors with 200-µm-wide TSVs that successfully carried up to 5A of DC current with a DC resistance of 23 mΩ. Examples of 3D TSV-based power inductors can be found in references^57,58^. The last steps in the TSV fabrication process are substrate planarization and routing conductor patterning.

**Fig. 6:**
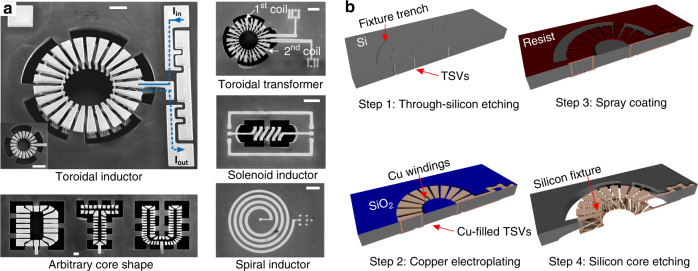
A TSV-based process for fabricating a 3D in-substrate toroidal inductor^57^ (Copyright Springer Nature 2018). **a** Fabricated inductors, including air-core toroidal inductors, a toroidal transformer, a solenoid inductor, a spiral inductor, and an inductor with an arbitrary “DTU” core shape. **b** Four main steps for fabricating the toroidal inductor^181^ (Copyright IEEE 2018)

Next, we discuss a representative TSV-based fabrication process for 3D in-substrate inductors for VHF power conversion. Figure 6a shows SEM micrographs of fabricated inductors and transformers, including toroidal inductors, a toroidal transformer, a solenoid inductor, a spiral inductor, and a “DTU” inductor with an arbitrary core shape. These inductors have unique features, such as in-substrate suspended windings that are held by five Si fixtures. The windings are separated from the Si substrate by an air gap of 300 µm, thus significantly reducing unwanted substrate effects. As shown in Fig. 6b, there are four main steps in the fabrication process: (i) TSV hole etching by DRIE and insulation layer deposition, including ALD Al_2_O_3_ and PECVD SiO_2_. The inductor core shape is defined by the fixture trench, as depicted in Fig. 6c. (ii) TSV bottom-up electroplating and winding patterning. (iii) Creation of a core-etching mask. (iv) Selective removal of a silicon core via isotropic dry etching by inductively coupled plasma (ICP) silicon etching, leaving Si fixtures protected by a layer of Al_2_O_3_ deposited on the fixture trench in step 1. More details of the process parameters can be found in reference^57^. This process has a unique ability to precisely remove an unwanted Si core to boost the inductor performance. The hollow air core, which can be fabricated in arbitrary core shapes, can be subsequently filled by magnetic particle composites to realize magnetic-core inductors with a higher inductance density^59^. The process is CMOS-compatible and scalable to a wide range of TSV diameters (30–50 µm) and substrate thicknesses (280–500 µm). A similar approach was also presented in recent studies on the in-substrate 3D solenoidal inductor^60^.

### Other processes: self-assembly processes and bonding techniques

**Fig. 7:**
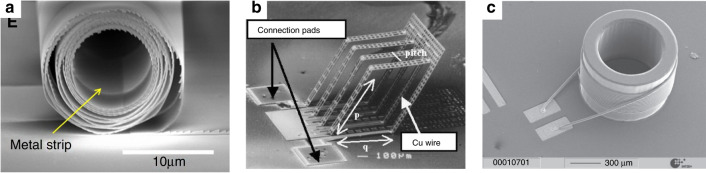
Other processes. SEM images of microinductors fabricated by **a** a self-rolled-up membrane (S-RUM)^63^ (Copyright Elsevier 2002) and **b** the surface micromachining and post release folding process^61^ (Copyright Springer Nature 2015). **c** Wire bonding^68^ (Copyright IOP 2010)

Self-assembly fabricates MEMS inductors by a post-micromachining step to arrange out-of-plane 2D or 3D inductor windings. An advantage of self-assembled inductors is the simplicity in fabricating complex winding structures without the need for excessive multilayer processing. Self-assembled inductor windings were originally planar conductors that are later released and assembled in a 3D fashion. Several mechanisms have been reported for inductor self-assembly, such as the post release folding process^61^ (Fig. 7b), plastic deformation magnetic assembly (PDMA)^62^, and self-rolled-up membrane (S-RUM)^63^ (Fig. 7a).

Taking the S-RUM process as an example, its self-assembly mechanism is achieved by depositing a bilayer of oppositely strained silicon nitride (SiN_x_). By etching an e-beam-evaporated Ge sacrificial layer to release a stack of multilayer thin films, the SiN_x_-Cu layers roll up, forming 3D tubular solenoidal inductors. An additional step to put ferrofluid into a solenoidal core to enhance the inductance density has also been demonstrated^64^. It has been reported that S-RUM tubular inductors have a high inductance density and reduced parasitic substrate effects. Moreover, the Cu thickness is critical for controlling the rolling step; therefore, thin Cu layers are preferred. Inductors with Cu layer thicknesses from 100 to 250 nm have been demonstrated. The same limitation applies to other self-assembly mechanisms that are applicable only for Cu thicknesses from hundreds of nanometers to a few micrometers, which are ideal for low-current applications, such as RFID^65^, RF applications^66^, and transformers for power transfer^67^.

Bonding technologies such as wire bonding and flip-chip bonding are also utilized for inductor fabrication. Wire-bonded inductors use bonded wires to construct 3D windings^68,69^ (Fig. 7c). The main advantages of this approach are the process simplicity and feasibility and the lower manufacturing costs compared to top-down microfabrication processing. Chip-to-chip bonding techniques such as flip-chip bonding could also be used to realize complex inductor windings by bonding chips, such as suspended spiral inductors^70^.

## Emerging 3D nanofabrication technologies

“MEMS fabrication of inductors” section reviewed top-down MEMS fabrication for inductor fabrication; in this section, we focus on emerging bottom-up methods. The trend of developing 3D structures using bottom-up fabrication methods has been stimulated by the global demand for environmentally friendly maskless high-resolution fabrication techniques along with the reduced use of hazardous chemicals and materials. Direct ink writing (DIW) is a simple and versatile additive manufacturing (AM) method to produce 3D structures with dimensions above 1 µm. Here, a wide range of materials can be printed by extruding printing materials through a nozzle. In the last 10 years, techniques such as fluidic forced microscopy (FluidFM), two-photon stereolithography (TPS), focused electron beam-induced deposition (FEBID), and ice lithography (IL) have emerged as bottom-up AM methods for high-precision three-dimensional manufacturing, enabling multilevel device prototyping in one printing step. The smallest structures made by these methods are 1 nm in size. Table 2 summarizes the main characteristics of the emerging AM techniques. These techniques have the potential to be employed as cost-effective and time-efficient ways of developing 3D micro- and nanostructures, including 3D microinductors.

**Table 2:** Main characteristics of nanoscale 3D direct-write technologies

Technology	Patterning method	Ink/precursor	Deposited materials	Minimal deposit dimension [nm]	Throughput [m/s]	Inductor and year
FluidFM^72^	Direct ink extrusion	shear thinning ink	nanoparticles, polymers	100–500	5 × 10^–4^	Helices, 2016^74^
TPS^76,171^	NIR lasers	photopolymers, composites	polymers, composites	<100	2 × 10^−2^	Multiturn microcoil, 2019^84^
FEBID^87,172^	E-beam	vapors of organic chemicals	metals, carbon compounds	~1	5 × 10^−8^	Moebius strip, 2019^87^
Ice lithography^95^	E-beam	condensed gasses	cross-linked chemicals	<5	4 × 10^−7^	½ turn coil, 2012^94^

### Fluidic forced microscopy

**Fig. 8:**
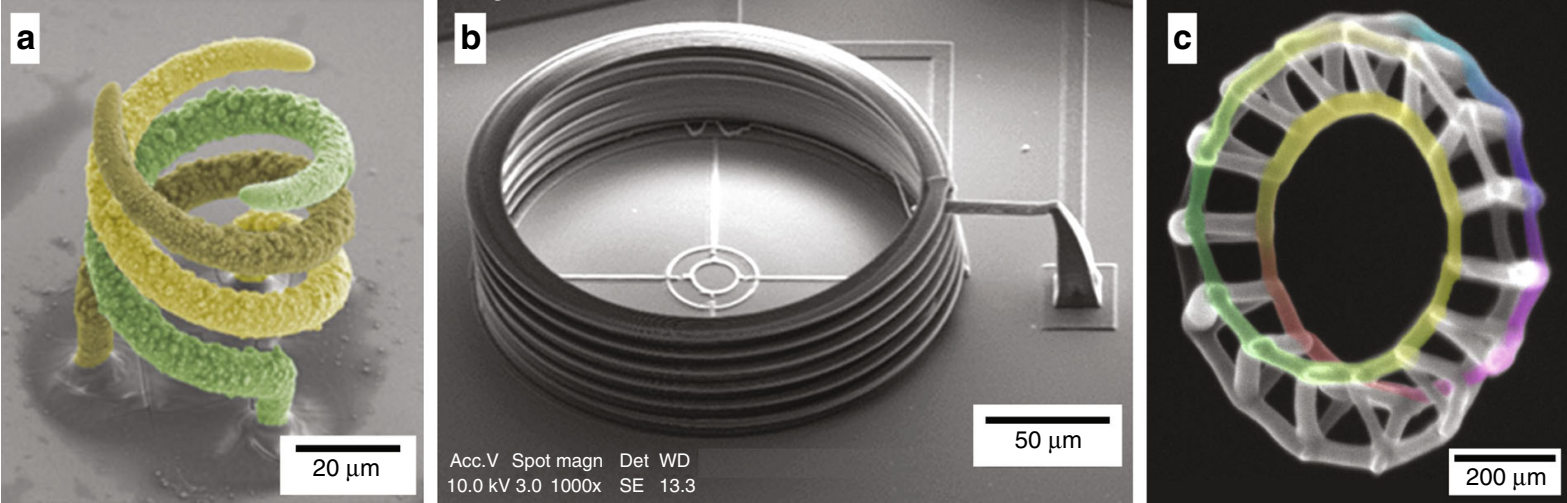
Microcoils and nanocoils made by emerging 3D nanofabrication technologies. SEM images of coil-like 3D microstructures: **a** Metallic 3D triple helix structure, printed with FluidFM^74^ (Copyright Wiley 2016). **b** 3D microcoil with a diameter of 200 μm, a height of 60 μm and five turns, printed using TPS^84^ (Copyright Leibert 2019). **c** 3D Moebius strip with a triangular cross section and individual wire dimensions of approximately 25 nm, fabricated by FEBID^87^ (Copyright AIP 2019)

FluidFM is a DIW method that takes advantage of the spatial precision of atomic force microscopy (AFM) along with the local ink delivery capability of microfluidics and nanofluidics. Thanks to the advancement of miniaturization technologies, AFM probes with integrated microscopic channels inside the tip enable microscale and nanoscale 3D printing via direct material delivery^71,72^. The microchanneled cantilevers have a flexible design, and the volumes they can handle are in the femtoliter to picoliter range^73^. FluidFM allows accurate printing on existing structures. The inks for the FluidFM deposition method are often composed of particulate and polymeric materials that are suspended or dissolved in a liquid solvent and solidify upon extrusion. A wide variety of materials, namely, polymeric, ceramic, metallic, hydrogel, and biomaterials, can be printed using this technique^71^. The viscosity of the printing materials is the most important property to tune for DIW additive deposition. The inks must exhibit pronounced shear-thinning behavior. 3D structures can be fabricated via layer-by-layer printing. In addition, the fabrication of metal structures and electrochemical 3D deposition with FluidFM have been reported^74^. Figure 8a shows a triple helix structure made by FluidFM and illustrates the inductor fabrication capabilities.

### Two-photon stereolithography

Two-photon stereolithography (TPS) implements a two-photon polymerization approach for patterning^75^. It uses high-intensity femtosecond laser pulse scanning through a photopolymer or photoresist in a layer-by-layer fashion to create microstructures and nanostructures in a single fabrication step. TPS utilizes the two-photon absorption of near-infrared (NIR) light to excite photosensitive materials. The total energy from two photons is resonant with the energy difference between the ground state and excited state. The TPS photopolymer is transparent in the NIR region; thus, the lasers penetrate deep into the materials and directly induce polymerization inside only the focal volume. The nonlinear two-photon process allows subwavelength fabrication, which is not possible by diffraction-limited stereolithography. Thus, TPS enables the fabrication of structures with dimensions below 100 nm^76^.

For selected TPS processes, negative-tone photoresists are used as photosensitive materials. Their solubility decreases with exposure to the dose of photons due to the cross-linking of monomers or oligomers in the photoresist. Free-radical-based or cation-based intermediates are applied to excite cross-linking, which can be initiated, respectively, by a photoinitiator or a photoacid generator^75^. Different polymeric materials and their composites, depending on the desired functionalities, are used for TPS. For example, to develop conductive structures, carbon nanotube (CNT)-based polymer composites have been employed^77^. Furthermore, Ag and Au salt and photoresist composites have been reported for metal deposition^78^. TPS has many applications, e.g., the development of microfluidics^79^, sensors^80^, actuators^81^, microbots^82^, and biomedical devices^83^. Ha et al.^84^ reported 3D microcoils (Fig. 8b) employing TPS using a dual-stage femtosecond laser scanning process. The coil was made of a hybrid material consisting of a polymer and a “metal-coatable polymer”. Electroless plating of silver was performed to achieve selective metallization of the metal-coatable polymer microstructure. The 3D microcoil with five turns, a diameter of 200 μm and a height of 60 μm can be operated at 25.4 GHz.

### Focused electron beam induced deposition (FEBID)

Based on electron-gas interactions, FEBID is a direct-write approach for the fabrication of 2D and 3D nanostructures. During FEBID, an electron beam (e-beam) is scanned over a sample in the presence of precursor gases to deposit patterns of materials onto a substrate^85^. Scanning electron microscope (SEM), along with a gas injection system, is employed for the FEBID method, and organic precursor gases are used as the printing materials. In FEBID, the material deposition (growth) process depends on several precursor-specific aspects, namely, the vapor pressure of the precursor, the adsorption characteristics of the precursor molecules, and their stability under adsorption. Under continuous e-beam exposure, the surface physisorbed or chemisorbed precursor molecules are dissociated. The low-energy secondary electrons and backscattered electrons generated by the primary electrons trigger the electron-induced dissociation process^86,87^. FEBID is particularly appropriate for microscale and nanoscale structure manufacturing since precursor molecules are small and e-beams can be focused onto spots with diameters varying from micrometers down to the angstrom level^88^. As examples, SEM images of a 3D magnetic double-loop nanospiral and Moebius strip with a triangular cross section and individual wire dimensions of approximately 25 nm are illustrated in Fig. 8c.

Taking advantage of the organometallic precursors designed for chemical vapor deposition, FEBID deposits metallic, superconducting alloys and intermetallic compounds and metamaterials^87,89^, such as silver (Ag), gold (Au), platinum (Pt), iron (Fe), tungsten (W), cobalt (Co), and ferrocobalt (Co_3_Fe) alloys. It enables a high degree of miniaturization that opens the way for a wide variety of applications. A purity of the as-deposited Au, Co, Fe, Si of 94–100%, Ag of ~75% and W of 66% has been reported for FEBID^90^. However, due to incomplete dissociation of the precursors, deposition from standard precursors, such as tungsten hexacarbonyl (W(CO)_6_), copper (II) hexafluoroacetylacetonate hydrate (Cu(hfac)_2_), and dimethyl-(1,1,1-trifluoro-2,4-pentandionato) gold (III) (Me_2_Au(tfac)), could result in deposition with a high carbon content. Therefore, a postdeposition purification process is developed to reduce contaminants by annealing in a reducing 98% N_2_ and 2% H_2_ atmosphere at elevated temperatures. For example, the purity of as-deposited ruthenium (Ru) was increased from 23 to 83% after postdeposition annealing at 300 °C.

### Ice lithography

**Fig. 9:**
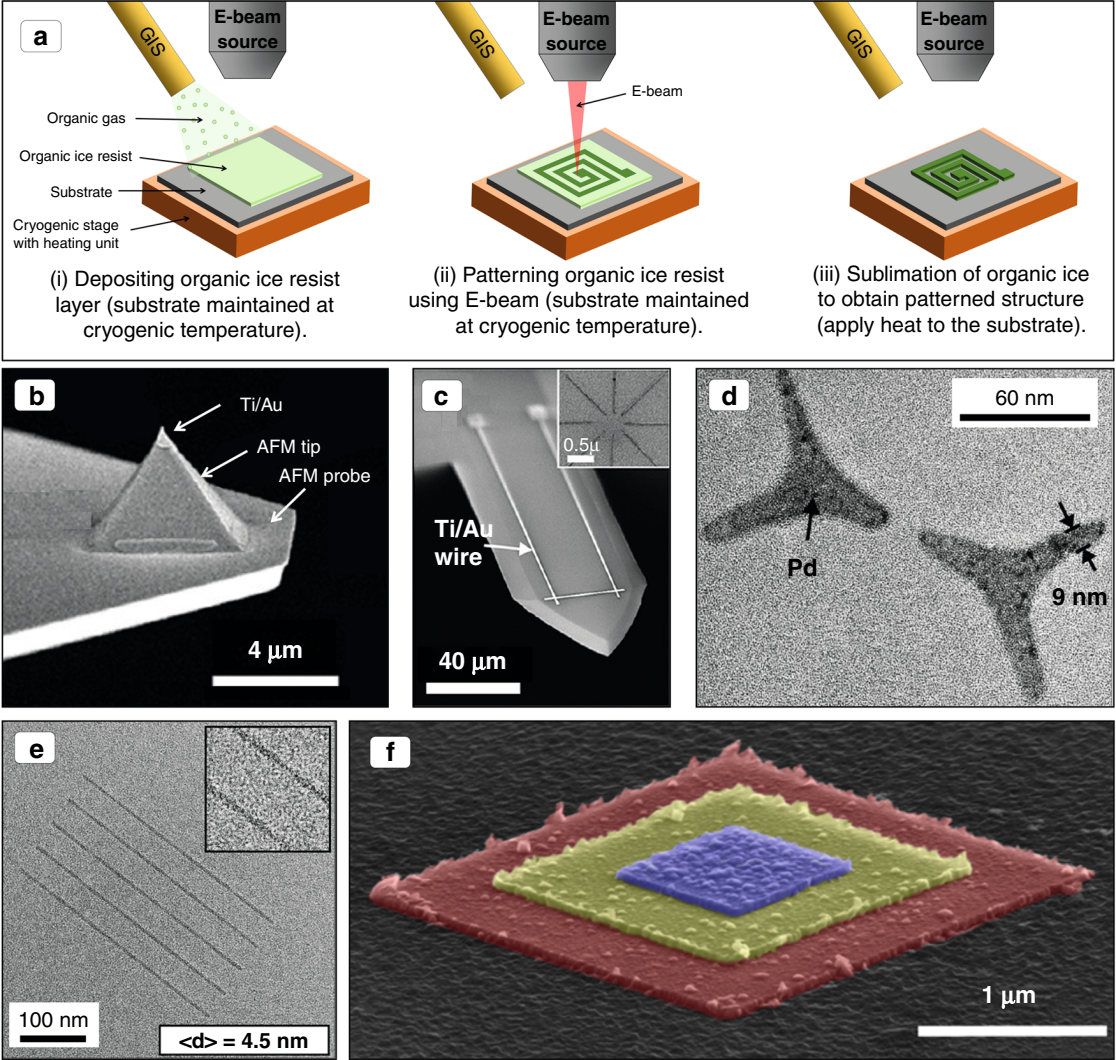
Ice lithography. **a** Schematic diagram of the process of ice lithography pattering. Example of ice lithography patterning, where the lift-off process is used to melt the ice layer^94^. **b** Patterning of the Ti/Au layer on an AFM tip^94^. **c** An SEM image of a patterned Ti/Au wire on a microcantilever. **d** A TEM image of a three-bladed pattern of palladium (Pd) metal patterns on a free-standing Si_3_N_2_ membrane^94^. **e** Patterned parallel lines on octane^95^. **f** SEM images of a 3D pyramidal nanostructure, made of Ag layers, fabricated at room temperature using a water ice resist for patterning^96^ (Copyright ACS 2012, 2018)

IL is a direct-write technique that uses electron-solid interaction principles for nanoscale and microscale fabrication^91^. During the IL process, organic precursor gas is condensed at cryogenic temperatures on the substrate under a vacuum to create an organic ice resist layer, and then a high-energy focused e-beam is used for cross-linking. Figure 9a summarizes the IL process. The e-beam cross-link condenses organic ice to form a large molecule network^92,93^. Thereafter, excess materials are removed by an evaporation step. Layer-by-layer deposition enables the printing of 3D structures. For the IL process, the focused electron energy is efficiently injected into the organic ice layer. Thus, in comparison to FEBID, IL is 1000 times faster^92^. Moreover, also true for FEBID, the IL process can be applied to substrates with complex geometries. A wide variety of precursors can be patterned by employing IL, considering the formation of an ice resist at cryogenic temperatures. In the case of highly impure and contaminated deposits, similar to FEBID, postdeposition purification processes can be added.

IL can pattern metals; here, the “lift-off” approaches were adapted to dissolve the ice layer^94^. For example, the manufacturing of a rectangular metallic cap on the pyramidal tip of a standard atomic force microscope cantilever is presented (Fig. 9b). Moreover, Au-Ti wires (0.5-μm wide and 300-μm long) with bonding pads are demonstrated on a microcantilever^94^ (Fig. 9c). An IL-patterned metallic layer on fragile support structures, where conventional techniques cannot be performed. A palladium (Pd) metallic triangular-shaped pattern with features below 10 nm on a fragile free-standing Si_3_N_2_ membrane (Fig. 9d) has also been presented. Elsukova et al.^95^ reported the use of a transmission electron microscope as a source of e-beam, which minimizes the influence of instrumental limitations, and showed that the onset dose of organic ice resists correlates with the inverse molecular weight of the organic compound. Continuous parallel lines down to 4.5 nm have been fabricated using a 0.4 pA beam current with frozen octane (C_8_H_18_), as presented in Fig. 9d. Furthermore, a 3D Ag-layered pyramidal structure (Fig. 9f) has been reported, where a water ice resist, which acts as a positive-tone resist, was utilized for patterning^96^. Ice lithography is a suitable candidate for developing ultraminiaturized microscale and nanoscale inductors in 2D and 3D forms for a wide range of applications due to its ability to produce fine features in fewer steps. We are currently developing IL to fabricate microinductors.

## Power electronics applications

### Power supply in package (PwrSiP) and power supply on chip (PwrSoC)

**Fig. 10:**
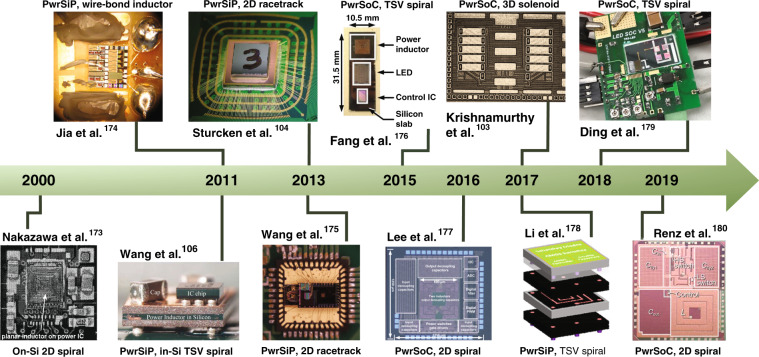
Overview of the timeline of notable developments of PwrSoC and PwrSiP using MEMS inductors. Copyright IEEE 2000, 2011, 2011, 2013, 2015, 2016, 2019, 2018, 2019

Power supplies play an essential role in all electronic devices. For example, they convert alternating current (AC) signals to direct current (DC) signals. In modern devices where space is strictly limited, including consumer electronic products, light-emitting diode (LED) lighting, wearables, implantable electronics, and the Internet of things (IoT), the miniaturization of power supplies has become a prime interest for future power supply generation. Monolithic integration is the ultimate solution to realize highly miniaturized power supplies while achieving superior performance in efficiency and power density. These integrated power supplies are the so-called power supply in package (PwrSiP) and power supply on chip (PwrSoC). Figure 10 illustrates a timeline overview of the notable developments of PwrSoC and PwrSiP.

In fact, in switched-mode power supplies (SMPSs), which are the most widely used power supply technology today, passive components such as inductors and transformers are essential, but they are often the most lossy and bulky components. Switching at higher frequencies allows using smaller inductors. For example, it was predicted that the target switching frequencies for PwrSiP and PwrSoC are at high frequencies (1–30 MHz) and very high frequencies (above 30 MHz), respectively^97^. Previous reviews cover technology trends, specifications, target applications, and enabling technologies for PwrSoC^97,98^. While other aspects of PwrSoC technology have greatly advanced, such as CMOS integration technologies for active components (e.g., high band-gap GaN, SiC FETs), gate drivers and controllers, and efficient topologies, integrated passive technology and 3D packaging technology remain technology gaps to overcome. Here, we discuss these two topics: (i) MEMS power inductor technology for PwrSoC and (ii) MEMS–CMOS integration and packaging technologies of SMPS in relation to MEMS inductor fabrication.

### MEMS inductors for PwrSiP and PwrSoC

**Fig. 11:**
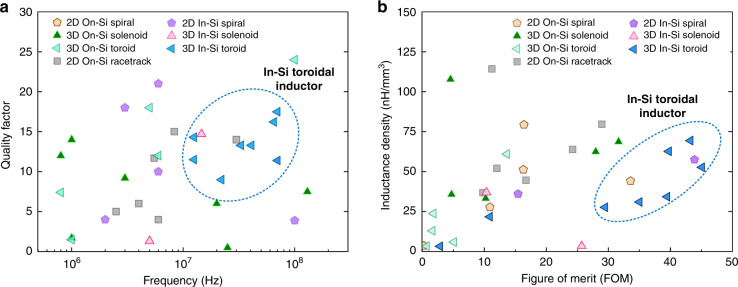
Characteristics of MEMS inductors that are used in power converters. **a** Quality factor versus frequency. **b** Inductance density versus a figure of merit (${\rm{FOM}} = \sqrt {Q_{{\rm{DC}}} \cdot Q_{{\rm{AC}}}} /V$)

First, MEMS inductors offer unique characteristics to be used in PwrSiP and PwrSoC applications thanks to the unique advantage of MEMS-CMOS integration. In addition to fundamental properties such as the quality factor and inductance, power MEMS inductors are required to have a sufficient current handling capability (100 mA to 3 A), low electromagnetic interference (EMI), low parasitic capacitance, etc. We summarize the electrical performance of state-of-the-art power MEMS inductors in Fig. 11. First, Fig. 11a presents the frequency-dependent quality factor versus the operating frequency. The presented inductor types have been demonstrated for power electronic applications in the frequency range of interest from 100 kHz to 30 MHz. While other groups of MEMS inductors cover a wide range of *Q* and frequency, 3D in-Si toroidal inductors stand out, with both a high quality factor (*Q*) and high frequency.

In power applications, an inductor often carries a DC current in addition to a high-frequency AC current. Therefore, it is important to also account for DC performance when evaluating the performance of the power MEMS inductor. Figure 11b shows the inductance density versus a figure of merit (FOM)^59^ for previous MEMS power inductors. We introduce the FOM to better scope the inductor efficiency at both DC and AC frequencies. The FOM is calculated using (1).

1 $${\rm{FOM}} = \sqrt {Q_{{\rm{DC}}} \cdot } Q_{{\rm{AC}}}/V = \sqrt {\frac{{L_{{\rm{DC}}}}}{{R_{{\rm{DC}}}}} \cdot } Q_{{\rm{max}}}/V$$

where *Q*_DC_ is the low-frequency quality factor, *Q*_max_ is the maximum quality factor, *V* is the total inductor volume, *L*_DC_ is the low-frequency inductance, and *R*_DC_ is the DC resistance. It is noted that the inductor substrate thickness is chosen to be 350 µm unless otherwise mentioned. It is shown in Fig. 11b that 3D in-Si toroidal inductors also outperform 3D on-Si toroidal inductors with higher inductance densities and FOMs, as shown in Fig. 11b. On the other hand, on-Si inductors cover a wider range of *Q* (3–19) and inductance density (25–110 nH/mm^3^).

While air-core MEMS inductors are useful for switching at very high frequencies (VHFs) above 22 MHz, magnetic-core MEMS inductors are needed for DC–DC power supplies below 22 MHz to achieve sufficient inductance. However, the advances in power conversion technologies have outpaced the growth in magnetics, and magnetic materials have become a key limitation constraining the overall miniaturization due to their excessive core losses at HF and VHF frequency ranges. Fundamental understandings of the core losses can be found in references^31,99^.

Developments in magnetic technology for power MEMS inductors have focused on exploring novel material combinations, optimizing core structures, and developing integration technology for magnetic materials. Electroplated ferrites, such as Ni_45_Fe_55_^100^, have been intensively studied due to their ease of fabrication, high permeability, and useful frequency range (MHz). Nanogranular cobalt-based alloys have recently received much attention due to their low core loss at high frequencies^26,101,102^. Notable studies of MEMS inductors with this material category include CoNiFe^38^, sputtered Co–Zr–O^26^, Ta/Co_91.5_Zr_4.0_Ta_4.5_ (CZT)/SiO_2_^101^, and sputtered cobalt-based amorphous alloys^102^. This approach often requires advanced deposition techniques for nanolaminated thin-film structures. Alternating electrodeposition and co-sputtering are the two most common deposition methods that have been demonstrated.

Another notable category of magnetic cores is composite cores. Using microscale and nanoscale magnetic particles that are electrically isolated, the eddy current loss can be significantly reduced, thus extending nanocomposite materials to high-frequency applications. Magnetic composites consist of magnetic particles that are electrically isolated either by insulator shells or by non-conducting media such as polymers. Casting or screen printing has the advantage of forming a large-volume magnetic core at low costs.

### Packaging technology for PwrSoC

For PwrSoC research, packaging technology plays a crucial role in improving the power density and efficiency for efficient thermal dissipation and minimal unwanted parasitics. For heat dissipation, high-thermal-conductivity materials such as Al_2_O_3_ and AlN are used as substrates and encapsulation materials. MEMS packaging technologies are adopted for PwrSoC integration as an alternative for common packaging architectures such as leaded-frame power modules and PCB-embedded power modules. There are 2D/2.5D packaging architectures that use wire bonding^103^ or flip-chip bonding techniques^104^. On the other hand, 3D packaging architectures refer to the vertical stacking of ICs using TSV-based Si interposers. Vertical stacking using TSV interconnections reduces the electrical path, thus minimizing the parasitics between vertically stacked chips. In addition, TSV-based packaging technology provides efficient thermal dissipation due to spreading effects over a thermally conductive substrate^105^, thus enhancing the power density and efficiency of PwrSoC. Important challenges are MEMS-CMOS integration of passive components such as inductors, TSV process optimization to maximize the current-carrying capability, and TSV insulator technology optimization to achieve CMOS compatibility and high-voltage isolation while keeping the TSV substrate thermal resistance low. Low-temperature ALD-enabled dielectrics, such as Al_2_O_3_ and AlN, are promising alternatives for dielectrics deposited by PECVD. Table 3 summarizes the key specifications of the power converters shown in Fig. 10, including the converter efficiency and power density corresponding to 2D, 2.5D, and 3D packaging technologies.

**Table 3:** Specifications of notable integrated power supplies using MEMS inductors, as presented in Fig. 10

Study	Year	Inductor @ Core material	Packaging	Converter specifications				
				*V*_in_ − *V*_out_ (*V*_DC_)	Freq. (MHz)	Eff. (%)	*P*_den_ (W/cm^2^)	*P*_den_ (W/cm^3^)
Nakazawa et al.^173^	2000	On-sub spiral @ CoHfTaPd	Integrated on IC	5–3	3	80	0.178	5.6
Jia et al.^174^	2011	Wire bond @ ferrite	Wire bond	5–2.2	5	52	0.371	–
Wang et al.^106^	2011	In-sub TSV spiral @ NiZn	3D, stacked, TSV interposer	3.6–1.8	6	80	10	76.9
Wang et al.^175^	2013	racetrack @ Ferrite	Co-packaged, wire bond	5–0.5	40	78	0.46	4.25
Sturcken et al.^104^	2013	Racetrack @ Ni_45_Fe_55_	2.5D, interposer, wire bond	1.8–1.2	100	71	1.89	41.7
Fang et al.^176^	2015	In-sub TSV spiral @ ferrite	3D, stacked, interposer	110 V_AC_ – 70 V_DC_	5	73	0.175	1.75
Lee et al.^177^	2016	On-sub spiral @ air	On-sub CMOS-integrated	2.2–1.2	500	76	0.76	115
Krishnamurthy et al.^103^	2017	On-sub 3D solenoid @ laminated Co-based amorphous	On-sub CMOS-integrated	1.5–1.2	100	84	11.7	234
Li et al.^178^	2017	In-sub TSV spiral @ NiZn	3D, stacked, TSV interposer	3.6–1.8	6	83	12	144.5
Ding et al.^179^	2018	In-sub TSV spiral @ ferrite	3D, stacked, TSV interposer	130–70	5	69	1.36	13.5
Renz et al.^180^	2019	On-sub spiral @ air	On-sub CMOS-integrated	4.5–1.8	47.5	85	0.054	5.5

### Challenges and research opportunities for power electronics

Power supply technology needs to keep up with the rapid development pace of modern electronics with respect to size reduction and performance improvement. Integrated power supplies have the potential to become an ideal solution. However, to increase the integration level, we envision the further development of four aspects.

First, further developments are needed to optimize the winding design and magnetic materials. We believe that inductors constructed by using 3D windings and TSV windings have great potential for the MHz switching frequency. 3D solenoid and toroidal windings have been shown to have better performance in terms of the inductance density and power density. Although these winding structures have been intensively studied, accurate models of winding losses are still needed to minimize losses due to eddy-current effects, especially the proximity effect.

Second, magnetic materials and deposition technologies are essential for power MEMS inductors. In addition to the deposition methods studied intensively, such as electroplating and sputtering, atomic layer deposition (ALD) is an emerging deposition method for fabricating nanolaminated magnetic thin films. ALD provides unique advantages, depositing alternating thin-film systems with superior flexibility and controllability over the film thickness, quality, and uniformity. The challenge is the limited variety of available magnetic materials that can be deposited by ALD. Superparamagnetic particles could also be interesting to investigate due to their extremely low eddy current loss regarding core losses. However, developing fabrication technology for the particle-based magnetic core is a challenge.

Third, packaging technology is another essential aspect of efficiently downsizing power supplies. We envision that the use of TSV-based interposer technology is a promising approach towards 3D integration of the power supply. Several intermediate steps are to be carried out towards monolithically integrated PwrSoC, including passive interposers, active interposers, and active-passive interposers. Several integrated power supplies have been demonstrated using passive interposers, such as inductive interposers^106,107^ and capacitive interposers^108^.

Finally, MEMS–CMOS integration is a crucial technology for PwrSoC integration. This technology would enable the monolithic integration of MEMS passive components (e.g., inductors and capacitors) and active components on the same substrate. A CMOS-compatible technology for a power MEMS inductor is needed.

## Biomedical and neurotechnology applications

MEMS inductors and magnetic devices also find important applications in the field of biomedical devices and systems, including microfluidics (nanobead trapping^109,110^, magnetic sensing^111,112^ and biotarget sorting^113^), nuclear magnetic resonance (NMR) spectroscopy^114,115^, implantable biomedical devices^116–118^, magnetic stimulation, and excitation (excitable cells^119^ and neurostimulation^116^). There are review papers covering the first three application categories, such as microfluidics^120^, magnetic sensing^111,121^, NMR^122^, wireless power transfer for implantable medical devices^123^, and NMR technologies for biomedical applications^124^. However, there is no review on the last application category. In this section, we focus on an exciting and growing area of microinductor research and application: neurotechnology.

Since the nervous system uses electrical signals to perform information transmission and to control the sensory organs, external electromagnetic fields can modulate neural activity. In 1985, Barker et al.^125^ demonstrated the stimulation of neurons using localized transcranial magnetic stimulation (TMS). Over the last 20 years, TMS, which uses rapidly fluctuating magnetic fields, has emerged as a noninvasive and painless technique to modulate brain function^126,127^ and brain mapping and to explore the excitability of different brain regions. Furthermore, TMS has been employed for the detection and monitoring of neurodegenerative diseases^128^ and for clinical therapy^129^ due to its ability to dampen neuronal hyperexcitability, decrease neuroinflammation, alter blood-brain-barrier permeability and promote neuronal survival.

**Fig. 12:**
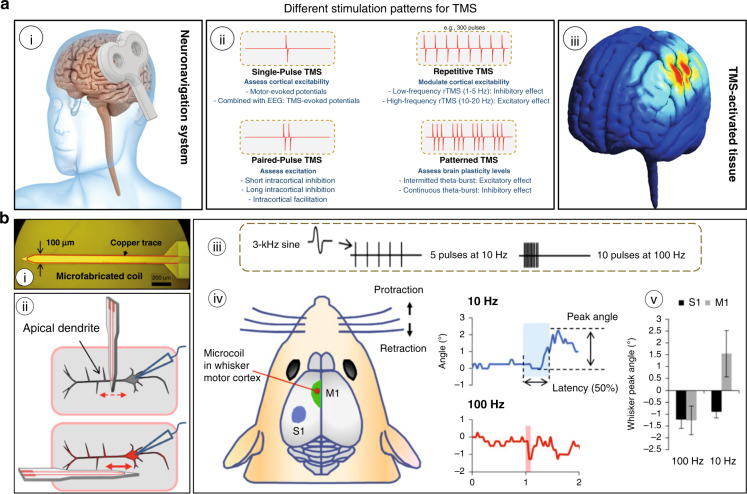
Magnetic brain stimulation techniques. **a** Transcranial magnetic stimulation (TMS) (adapted from reference^127^, Copyright Elsevier 2020): (i) The coil induces a strong magnetic field exciting or inhibiting cortical activity. (ii) A summary of different formulations of pulse patterns—different protocols can be implemented by pairing more pulses with various effects. (iii) Simulated area of activation by the TMS. **b** Silicon MEMS implant with a half-turn coil (adapted from reference^116^, Copyright AAAS 2016): (i) The microfabricated microcoil implant. (ii) Schematic of the coil orientation over the apical dendrites of cortical pyramidal neurons: (top) perpendicular orientation, which resulted in very weak electric fields along the neuron and did not produce spiking, and (bottom) parallel orientation, which produced robust spiking. (iii) Stimulus waveforms composed of pulses that consisted of one full period of a 3-kHz sinusoid with an amplitude of 112 mV. (iv) Coils inserted into the whisker motor cortex (M1). Ten-hertz stimulation resulted in protraction of the whiskers (upward deflections) on the right side (top), whereas 100-Hz stimulation induced retraction (downward deflections) (bottom). (v) Mean amplitudes of the peak whisker movements for each stimulus condition

The magnetic flux created due to dynamic current flow through the coil generates its own electric field^130^. This electric field induces changes in transmembrane currents, eventually leading to the depolarization or hyperpolarization of neurons, making them, respectively, more or less excitable^131^. Depending on the frequency, duration, and intensity of stimulation, the effects of TMS can be varied^132^. Low-frequency and high-frequency stimulation patterns can, respectively, exert dampening of neural activity and excitatory effects on brain activity^133^. Single-pulse and paired-pulse TMS are used to examine the functionality of the brain, whereas repetitive TMS is utilized to induce long-lasting changes in brain activity beyond the stimulation period, as summarized in Fig. 12a. Chronic prosthetic applications require deeper brain activation with accurate focusing of specific neural targets.

### Micromagnetic stimulation (μMS)

**Fig. 13:**
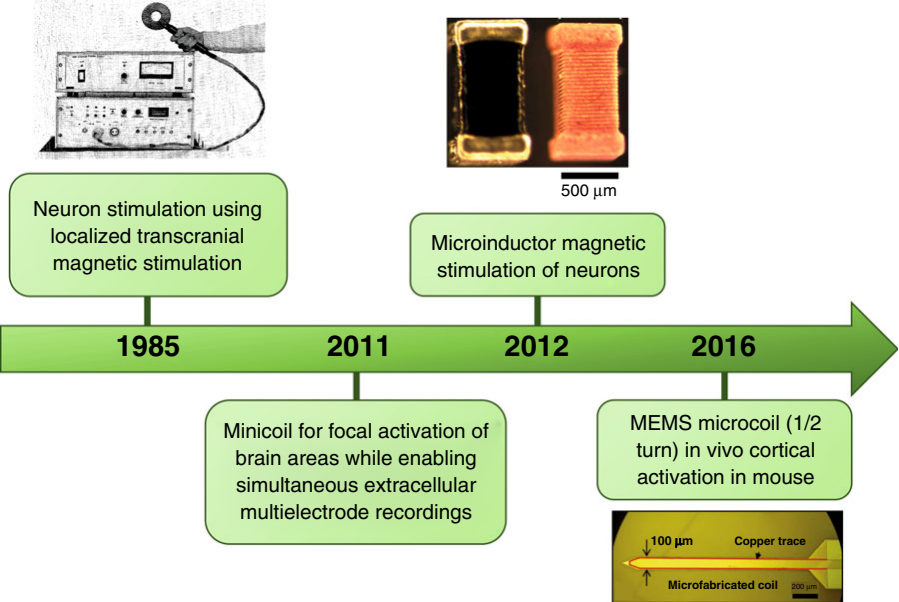
Timeline of the discoveries and development in the field of micromagnetic neurostimulation. Transcranial stimulation^125^ (Copyrights Elsevier 1985), minicoils^182^, microinductor^134^ and MEMS inductor neurostimulation^116^, Springer Nature 2012, AAAS 2016

μMS uses an implanted MEMS microcoil to produce a magnetic field. Unlike TMS, which uses a relatively large coil, e.g., often 50–100 mm in diameter, that is positioned above the scalp (Fig. 12a) and typically targets large regions in the superficial cortex (e.g., several centimeters or more in diameter), the use of MEMS allows coils to be implanted into the brain and may facilitate confined activation of narrow regions of the brain or even a single neuron. Bonmassar et al. reported, for the first time in 2012^134^, that the electric currents flowing through the commercially available microcoil inductor generated adequate time-varying magnetic fields and, in the process, could induce electric currents in a focal area to elicit neuronal activity. This development raises new possibilities of using a micromagnetic stimulation inductor as neural prosthetics, which would allow implantation near the areas of interest to stimulate deeper brain targets with higher spatial selectivity. Figure 13 summarizes the timeline of development and the applications of noninvasive micromagnetic stimulation of brain activation.

For example, Lee et al.^135^ used a commercial solenoid coil (0.5 mm in diameter) to stimulate subthalamic nucleus (STN) neurons, i.e., the primary target of deep brain stimulation for the treatment of Parkinson’s disease. A similar approach was reported by Minusa et al.^136^, where microcoil magnetic stimulation using the same solenoid coil was employed for activation of the auditory cortex of anesthetized mice. The results show that µMS can selectively target local neural networks and modulate their activities in vivo. Unfortunately, because commercial microcoils are not designed for implantation into the cortex, tissue damage during implantation is a serious problem^135^. Therefore, dedicated MEMS coil implants are needed.

In 2016, the first MEMS microcoil-based neural prosthetics were reported^116^. The coil had a half turn because it was fabricated using the 2D lithography method. The width and length of the implant were 100 μm and 2 mm, respectively. The implant was tested in living mouse subjects. Despite generating a weaker electric field than that of large TMS coils, the field was confined to a much smaller region, and therefore, the spatial gradient of the field was strong enough to activate neurons in both in vitro and in vivo experiments. The implant was inserted into the mouse whisker motor cortex, and depending on the stimulation frequency, the whiskers exhibited movements in response to microcoil stimulation (Fig. 12b). Although this inductor is thin and small, there is still room for improvement in terms of its magnetic field strength and selectivity. A study showed that the coil design (e.g., shape and number of turns) influenced the selectivity and strength of the microcoil^137^. Several microcoil designs, namely, rectangular, V-shaped and W-shaped designs, were studied numerically and experimentally in vitro using coronal brain slices from the mouse primary visual cortex. The results show that both the V-shaped and W-shaped designs can reliably activate neurons. The W-shaped coil design provides higher selectivity, whereas the V-shaped coil design renders a higher strength of stimulation. Moreover, when compared to the single-turn coil design, the multiturn coil exhibits enhanced strength of stimulation and thus reduces the activation thresholds. The findings present guidelines for the development of next-generation microcoil-based neural prostheses.

### Challenges and research opportunities for neurotechnologies

Since its first manifestation in the mid-1980s, the TMS method has evolved to overcome some of the issues associated with electric brain stimulation^138^, such as pain links to the stimulation process and tissue damage. However, the noninvasive nature of TMS limits its specificity and focality. This makes deeper brain area activation difficult without affecting greater regions than the targeted location. Since divergent magnetic stimulation could increase the risk of seizures and other side effects^139^, design optimization of the appropriate coil has become increasingly important, and challenging to improve the focality for brain stimulation^140^. The multichannel TMS approach^141^, where multiple independent coils are simultaneously operated with altered magnitudes of current to produce a magnetic field profile in demand, has also been developed to overcome issues. Although the multichannel approach shows promise, the energy and thermal dissipation still need to be addressed. Another drawback of the TMS is variability, which contributes to uncertainty and inefficiency. Often, the results of the TMS are inconsistent. A study showed that a particular sequence of pulse patterns could produce different responses for different persons^140^.

Progress in µMS employing microcoil implants allows for ultrafocused intracortical stimulation and focal cortical responses around coil implants. Compared to TMS, micromagnetic stimulation coils can be positioned in close proximity to the target region, which helps to improve the spatial control of the elicited activity. Additionally, compared to recently developed implantable neural stimulators powered by using inductive coupling coils^142^, magnetic transducers^143,144^, and ultrasound transducers^145,146^, microcoil implants have important advantages. For example, regardless of the method of wireless power transmission, since most wireless stimulators generally deliver direct electric stimulation to targeted neurons via implanted electrodes, there are several limitations, such as limited control of the electric fields (e.g., spatially symmetric electric fields) and decreased performance associated with tissue inflammation (e.g., glial scarring). In contrast, microcoil implants can produce spatially asymmetric electric fields around the coils, thereby selectively targeting specific neurons while avoiding passing axons. This enhanced selectivity suggests that coil implants can provide better stimulation resolution than electrode-based implants. Additionally, since the magnetic field has high permeability to biological materials, the efficacy of magnetic stimulation from the coil implants will not be diminished even by severe tissue encapsulation. Thus, microcoil implants have the potential to improve the effectiveness and reliability of implantable neural stimulators.

Since magnetic fields can also pass through nonmagnetic insulating materials, microcoils can be fully encapsulated using a wide range of flexible biocompatible materials, e.g., Parylene^147^, liquid crystal polymer^148^, and hydrogel^149^; this will help to reduce the inflammatory tissue reactions that can arise in response to chronic implantation in the cortex. This encapsulation also enables the μMS coils to be electrically isolated from the adjacent tissue and may also reduce the amount of heat transfer. However, regarding the mechanisms underlying µMS induction, e.g., modulation of the activity of single neurons or cortical neural networks, we have a very limited understanding^150,151^.

For neural prosthetic implants with ultrafine stimulation resolution, we envision opportunities and challenges in four areas. First, since a 3D microcoil requires dimensions similar to those of neuron cells (20 µm), there are significant fabrication challenges in fabricating multiturn inductors with conductors approximately 1 µm in diameter and in performing magnetic core integration to achieve a high inductance density. Second, unlike the RF and power electronics applications, inductors for neural stimulations must induce large electrical field gradients with low power consumption. Hence, the inductor designs are very different and have only marginally been explored. Third, for medical applications in artificial vision, an array of millions of inductors is needed, and the array must be implanted in the visual cortex with a complex topology. The challenge is to make individualized, flexible, multiplexed, and durable implants. Fourth, since there are 30 million blind people around the world and most patients cannot afford expensive implants, the final challenge is to provide accessible technology.

## Supplementary information

Data for figures 1 and 3

Table: MEMS inductor categories and their characteristics
