# Supplementary material for: MEMS inductor fabrication and emerging applications in power electronics and neurotechnologies
Source: Microsyst Nanoeng. 2021 Aug 11;7:59. doi: 10.1038/s41378-021-00275-w (PMC8433479; doi:10.1038/s41378-021-00275-w)
Supplement: Supplementary file 1 — Data for figures 1 and 3 [file 41378_2021_275_MOESM1_ESM.pdf]

MEMS inductor fabrication and emerging applications in power electronics and neurotechnologies

Hoa Le, Rubaiyet Haque, Ziwei Ouyang, Seung Woo Lee, Shelley Fried, Ding Zhao, Qiu Min, and Anpan Han

Data for Figure 1 and 3: The papers are sorted for four applications: (i) RF MEMS, (ii) Power Electronics, (iii) Biomedical and Neurotechnology Applications, and (iv) Wireless and Sensors.

I. **RF MEMS**:

	Ref.	Author Year	Fabrication Technology
1.	[1]	Nhat M. Nguyen 1990	2D spiral (2D surface micromachining)
2.	[2]	Matsuki et al. 1991	2D spiral (2D surface micromachining)
3.	[3]	(Ahn and Allen 1992)	3D meander toroidal (3D surface micromachining)
4.	[4]	(Ahn and Allen 1993)	Stacked 2D spiral (2D surface micromachining)
5.	[5]	(Ahn and Allen 1994)	3D toroidal (3D surface micromachining)
6.	[6]	(Reyes et al. 1995)	2D spiral (2D surface micromachining)
7.	[7]	(Long and Copeland 1995)	2D spiral (2D surface micromachining)
8.	[8]	(Rofougaran et al. 1996)	2D spiral (2D surface micromachining)
9.	[9]	(A. Rofougaran et al. 1996)	2D spiral (2D surface micromachining)
10.	[10]	(Y. Sun et al. 1996)	2D spiral (2D surface micromachining), suspended
11.	[11]	(Zu et al. 1996)	2D spiral (2D surface micromachining)
12.	[12]	(Nam and Kwon 1997)	2D spiral (2D surface micromachining)
13.	[13]	(J. Y. P. and M. G. Allen 1997)	3D solenoid (3D surface micromachining)
14.	[14]	(Ahn and Allen 1998)	2D spiral (2D surface micromachining)
15.	[15]	(Rofougaran et al., 1998)	2D spiral (2D surface micromachining)
16.	[16]	(Ribas et al.1998)	2D spiral (2D surface micromachining)
17.	[17]	(Yoon et al. 1998)	3D solenoid (3D surface micromachining)
18.	[18]	(Kim et al. 1998)	3D solenoid (3D surface micromachining)
19.	[19]	(Yoon et al. 1998)	3D solenoid (3D surface micromachining)
20.	[20]	(Park et al. 1998)	2D spiral (2D surface micromachining)
21.	[21]	(Ribas et al. 1998)	2D spiral (2D surface micromachining)
22.	[22]	(Young et al. 1998)	2D spiral (2D surface micromachining)
23.	[23]	(Yoon et al. 1998)	3D solenoid (surface micromachining)
24.	[24]	(Fan et al. 1998)	2D spiral (2D surface micromachining), Others: self-assembly
25.	[25]	(Kim and Allen 1998)	3D solenoid (surface micromachining)
26.	[26]	(Liakopoulos and Ahn 1999)	3D toroidal (surface micromachining)
27.	[27]	(Park and Allen 1999)	2D spiral (2D surface micromachining)
28.	[28]	(Yoon et al. 1999)	2D spiral (2D surface micromachining)
29.	[29]	(Yamaguchi et al. 1999)	2D spiral (2D surface micromachining)
30.	[30]	(Yoon and Han 1999)	2D/3D stacked-spiral (surface micromachining)
31.	[31]	(Yue and Wong 2000)	2D spiral (2D surface micromachining)
32.	[32]	(López-Villegas et al. 2000)	2D spiral (2D surface micromachining)

33.	[33]	(Chomnawang and Lee 2001)	3D solenoid (surface micromachining)
34.	[34]	(Yamaguchi et al. 2001)	2D spiral (2D surface micromachining)
35.	[35]	(Liang et al. 2002)	3D solenoid (3D surface micromachining)
36.	[36]	(Gel et al. 2002)	Others: self-assembly (2D surface micromachining)
37.	[37]	(Massin et al. 2002)	2D spiral (2D surface micromachining)
38.	[38]	(Brunet et al. 2002)	2D racetrack (2D surface micromachining)
39.	[39]	(Lakdawala et al. 2002)	2D spiral (2D surface micromachining)
40.	[40]	(Burghartz et al. 2003)	2D spiral (2D surface micromachining)
41.	[41]	(Cao et al. 2003)	2D spiral (2D surface micromachining)
42.	[42]	(Zou et al. 2003)	Others: Self-assembly
43.	[43]	(M. G. Allen 2003)	3D solenoid (3D surface micromachining)
44.	[44]	(Chua et al. 2003)	3D self-assembly (2D surface micromachining)
45.	[45]	(Chen and Liou 2004)	2D spiral (2D surface micromachining)
46.	[46]	(Wang et al. 2004)	2D spiral (2D surface micromachining)
47.	[47]	(Pan et al. 2004)	2D in-Sub Spiral (2D substrate-embedded)
48.	[48]	(Pisani et al. 2004)	2D spiral (2D surface micromachining)
49.	[49]	(Scott et al. 2004)	3D solenoid/toroidal (3D surface micromachining), self-assembly
50.	[50]	(Zhuang et al. 2005)	3D solenoid/toroidal (3D surface micromachining)
51.	[51]	(Raieszadeh et al. 2005)	2D spiral (2D surface micromachining), thick oxide
52.	[52]	(Rais-Zadeh and Ayazi 2005)	2D spiral (2D surface micromachining), thick oxide
53.	[53]	(Yoon and Allen 2005)	3D solenoid (3D surface micromachining), UV-LIGA
54.	[54]	(Turitsyna and Webb 2005)	2D spiral (2D surface micromachining), thick oxide
55.	[55]	(Dixit and Miao 2006)	In-Sub TSV process development
56.	[56]	(Lei et al. 2006)	3D solenoid (3D surface micromachining)
57.	[57]	(Seemann and Bekker 2006)	3D solenoid (3D surface micromachining)
58.	[58]	(Fang et al. 2006)	3D solenoid (3D surface micromachining)
59.	[59]	(Fang et al. 2007)	3D solenoid (3D surface micromachining)
60.	[60]	(Bell et al. 2007)	Others: Flip-Chip
61.	[61]	(Lu et al. 2007)	3D solenoid (3D surface micromachining), UV-LIGA
62.	[62]	(Tai and Liao 2007)	Stacked spiral (2D surface micromachining)
63.	[63]	(Zhuang et al. 2007)	3D solenoid (3D surface micromachining)
64.	[64]	(L. Gu and Li 2007)	3D solenoid (3D surface micromachining), suspended
65.	[65]	(Mekaru et al. 2007)	Others: Molding using 3D-LIGA
66.	[66]	(Zine-El-Abidine and Okoniewski 2007)	3D solenoid (3D surface micromachining)
67.	[67]	(C. Xue et al. 2008)	2D spiral (2D surface micromachining)
68.	[68]	(Hsieh et al. 2008)	2D spiral (2D surface micromachining), suspended
69.	[69]	(Park et al. 2008)	2D spiral (surface micromachining), UV-LIGA
70.	[70]	(Wu and Zaghloul 2008)	2D spiral /3D solenoid (surface micromachining)
71.	[71]	(Lee et al. 2009)	2D spiral (2D surface micromachining)
72.	[72]	(Kratt et al. 2010)	Others: wire-bond
73.	[73]	(Ding et al. 2010)	2D spiral (2D surface micromachining)
74.	[74]	(Artillan et al. 2011)	2D spiral (2D surface micromachining)
75.	[75]	(Mohamed Ali et al. 2011)	2D spiral (2D surface micromachining)
76.	[76]	(P. Shen et al. 2011)	2D spiral (2D surface micromachining)

77.	[77]	(Yang, Dai, and Hong 2011)	2D spiral (2D surface micromachining), suspended
78.	[78]	(Vitale et al. 2012)	2D in-sub planar inductor (TSV)
79.	[79]	(Meyer et al. 2012)	Stacked spiral (2D surface micromachining)
80.	[80]	(Zhan et al. 2012)	Stacked spiral (2D surface micromachining)
81.	[81]	(Xue et al. 2012)	Stacked spiral (2D surface micromachining)
82.	[82]	(Li et al. 2012)	Stacked 2D in-sub spiral inductor (TSV)
83.	[83]	(Ebefors et al. 2013)	3D in-sub toroidal (TSV)
84.	[84]	(F. Liang et al. 2013)	2D in-sub spiral inductor (TSV)
85.	[85]	(Mohamed Ali et al. 2013)	2D spiral (2D surface micromachining), wireless SMA actuators
86.	[86]	(Moazenzadeh et al. 2013)	Others: wire-bond
87.	[87]	(Y. Gao et al. 2014)	3D solenoid (3D surface micromachining)
88.	[88]	(Jitae Kim et al. 2014)	3D in-sub solenoid (TSV)
89.	[89]	(Kahlouche et al. 2014)	2D spiral (2D surface micromachining)
90.	[90]	(Kim and Kim 2015)	2D in-sub spiral inductor (substrate-embedded)
91.	[91]	(Rahman et al. 2015)	2D spiral (2D surface micromachining)
92.	[92]	(S. Y. Wu et al. 2015)	Others: Printing
93.	[93]	(Vitale et al. 2015)	3D in-sub solenoid (TSV)
94.	[94]	(Kifle and Koul 2015)	2D spiral (2D surface micromachining), thick oxide
95.	[95]	(Huang et al. 2015)	Others: self-rolled-up, RFIC
96.	[96]	(Sato et al. 2016)	2D spiral (2D surface micromachining)
97.	[97]	(Zhang et al. 2016)	2D in-sub spiral inductor (TSV)
98.	[98]	(S. Hsieh et al. 2016)	3D solenoid (3D surface micromachining)
99.	[99]	(X. Li et al. 2016)	Review: Micromachined RF MEMS inductors
100.	[100]	(H. Lee et al. 2016)	Others: Printing
101.	[101]	(Thadesar and Bakir 2016)	2D in-sub spiral inductor (TSV)
102.	[102]	(Ren et al. 2016)	2D in-sub spiral inductor (TSV)
103.	[103]	(Y. C. Hsieh et al. 2016)	3D in-sub solenoid (TSV)
104.	[104]	(Dhahri et al. 2016)	2D in-sub spiral inductor (TSV)
105.	[105]	(Xu and Luo 2017)	2D spiral (2D surface micromachining), suspended
106.	[106]	(Wang et al. 2017)	2D spiral (2D surface micromachining), suspended
107.	[107]	(Vaseem and Shamim 2017)	Others: Printing
108.	[108]	(White and Wang 2017)	3D solenoid (3D surface micromachining)
109.	[109]	(J. Gu et al. 2017)	3D in-sub solenoid (TSV), Others: micro-casting method
110.	[110]	(Serrano et al. 2017)	3D in-sub solenoid (TSV), nanowire
111.	[111]	(Fernández-Bolaños et al. 2017)	3D in-sub solenoid (TSV)
112.	[112]	(Cho, and Kim 2017)	3D in-sub solenoid (TSV)
113.	[113]	(Bedair and Lazarus 2017)	2D spiral (2D surface micromachining), varied winding thickness
114.	[114]	(W. Huang et al. 2017)	Others: self-assembly rolled-up, RFICs and MMICs
115.	[115]	(Viallon et al., 2018)	3D solenoid (3D surface micromachining)
116.	[116]	(Yunheng Sun et al. 2018)	2D in-sub spiral inductor (TSV)
117.	[117]	(Lopez-Villegas et al. 2018)	Others: Printing
118.	[118]	(Zia, Oh, and Bakir 2018)	3D solenoid (3D surface micromachining)
119.	[119]	(Huang et al. 2018)	Others: self-assembly rolled-up
120.	[120]	(Huang and Yu 2018)	2D in-sub spiral inductor (TSV)

121.	[121]	(Huang and Li 2018)	2D spiral (2D surface micromachining), graphene
122.	[122]	(Wang et al. 2018)	3D solenoid (3D surface micromachining)
123.	[123]	(Sun et al. 2018)	3D in-sub solenoid (TSV)
124.	[124]	(Kang et al. 2018)	2D spiral (2D surface micromachining), graphene
125.	[125]	(Maganti et al., 2018)	2D spiral (2D surface micromachining)
126.	[126]	(Ming 2018)	3D in-sub solenoid (TSV)
127.	[127]	(Eblabla et al. 2018)	2D spiral (2D surface micromachining
128.	[128]	(Hu et al. 2018)	2D in-sub spiral inductor (TSV)
129.	[129]	(Z. Ali et al. 2019)	2D spiral (2D surface micromachining), CNTs
130.	[130]	(Hirmer et al. 2019)	Others: wire-bond
131.	[131]	(Michel et al. 2019)	3D solenoid (3D surface micromachining)
132.	[132]	(Bajwa and Yapici 2019)	Others: self-assembly, (2D surface micromachining)
133.	[133]	(Pares et al. 2019)	3D solenoid (3D surface micromachining)
134.	[134]	(Prabhakaran and Son 2019)	Others: Printing
135.	[135]	(Li et al. 2019)	2D in-sub spiral inductor (TSV)
136.	[136]	(Yin et al. 2019)	2D in-sub spiral inductor (TSV)
137.	[137]	(Zhang et al. 2020)	3D in-sub solenoid (TSV)
138.	[138]	(Wang et al. 2020)	2D in-sub planar stripes (TSV)
139.	[139]	(Freitas et al. 2020)	2D spiral (2D surface micromachining), flexible substrate
140.	[140]	(Prawoto et al. 2020)	2D spiral (2D surface micromachining)
141.	[141]	(Khan and Younis 2020)	Others: Wire-bond
142.	[142]	(Shousha and Haug 2020)	3D solenoid (3D surface micromachining)
143.	[143]	(H. Chen et al. 2020)	3D solenoid (3D surface micromachining)

II. Power Electronics:

No.	Ref.	Author Year	Fabrication Technology
144.	[144]	(Masato Mino et al. 1992)	3D solenoid (surface micromachining)
145.	[145]	(Sullivan and Sander 1993)	2D racetrack (2D surface micromachining)
146.	[5]	(Ahn and Allen 1994)	Meander 3D solenoid (surface micromachining)
147.	[146]	(Sato et al. 1994)	2D racetrack (2D surface micromachining)
148.	[147]	(Kim and Allen 1994)	3D solenoid (surface micromachining)
149.	[148]	(M. Mino et al. 1995)	3D solenoid (surface micromachining)
150.	[149]	(Sanders 1995)	2D racetrack (2D surface micromachining)
151.	[150]	(Masato Mino et al. 1996)	2D racetrack (2D surface micromachining)
152.	[151]	(Ahn and Allen 1996)	3D solenoid (surface micromachining)
153.	[152]	(Sullivan et al. 1996)	2D racetrack (2D surface micromachining)
154.	[153]	(Sullivan and Sanders 1996b)	2D racetrack (2D surface micromachining)
155.	[154]	(Sullivan and Sanders 1996a)	2D racetrack (2D surface micromachining)
156.	[155]	(T. Sato et al. 1996)	2D spiral (2D surface micromachining
157.	[13]	(J. Y. P. and M. G. Allen 1997)	3D solenoid (surface micromachining)
158.	[156]	(Xu et al. 1998)	3D solenoid (surface micromachining)
159.	[157]	(Inoue et al. 1998)	2D spiral (2D surface micromachining)
160.	[158]	(Sugahara et al. 1998)	2D spiral (2D surface micromachining)

161.	[26]	(Liakopoulos and Ahn 1999)	3D toroidal (surface micromachining)
162.	[27]	(Park and Allen 1999)	2D spiral (2D surface micromachining)
163.	[159]	(Liakopoulos and Ahn 1999)	3D toroidal (surface micromachining)
164.	[160]	(Coonley and Sullivan 1999)	2D V-groove Inductor (2D surface micromachining)
165.	[26]	(Liakopoulos and Ahn 1999)	3D toroidal (surface micromachining
166.	[161]	(Sullivan and Sanders 1999)	2D racetrack (2D surface micromachining)
167.	[162]	(Park et al. 1999)	3D solenoid (surface micromachining)
168.	[163]	(Park and Allen 2000)	3D solenoid (surface micromachining)
169.	[164]	(Nakazawa et al. 2000)	2D spiral (2D surface micromachining)
170.	[165]	(Prabhakaran et al. 2000)	2D V-groove Inductor (2D surface micromachining)
171.	[166]	(Katayama et al. 2000)	2D spiral (2D surface micromachining)
172.	[167]	(Sato et al. 2001)	2D spiral (2D surface micromachining)
173.	[168]	(Park and Bu 2001)	2D spiral (2D surface micromachining)
174.	[169]	(Sato et al. 2001)	2D racetrack (2D surface micromachining)
175.	[170]	(Kim et al. 2001)	2D spiral (2D surface micromachining)
176.	[171]	(Kim et al. 2001)	2D racetrack (2D surface micromachining)
177.	[172]	(Brunet et al. 2001)	2D racetrack (2D surface micromachining)
178.	[173]	(Lotfi and Wilkowski 2001)	Review on Magnetics for HF Switching Power Supplies
179.	[174]	(Boggetto et al. 2002)	2D racetrack (2D surface micromachining)
180.	[175]	(Park et al. 2002)	3D toroidal (surface micromachining)
181.	[38]	(Brunet et al. 2002)	2D racetrack (2D surface micromachining)
182.	[176]	(Park et al. 2003)	2D racetrack (2D surface micromachining)
183.	[177]	(Prabhakaran et al. 2003)	2D V-groove Inductor (2D surface micromachining)
184.	[178]	(Musunuri and Chapman 2003)	Stacked spiral (2D surface micromachining)
185.	[179]	(Park and Allen 2003)	3D solenoid (surface micromachining)
186.	[180]	(Fukuda et al. 2003)	2D spiral (2D surface micromachining)
187.	[181]	(Brandon et al. 2003)	2D spiral (2D surface micromachining)
188.	[182]	(Arnold et al. 2004)	Laminated magnetic core, 3D solenoid
189.	[183]	(Donnell et al. 2004)	2D racetrack (2D surface micromachining)
190.	[184]	(Troussier et al. 2004)	2D spiral (2D surface micromachining)
191.	[185]	(Ningning Wang et al. 2004)	2D racetrack (2D surface micromachining)
192.	[186]	(Park et al. 2004)	2D spiral (2D surface micromachining), 3D laminated core
193.	[187]	(Martincic et al. 2004)	2D racetrack (2D surface micromachining), Flip-Chip
194.	[47]	(Pan et al. 2004)	2D in-Si Spiral (substrate-embedded)
195.	[188]	(Laur et al. 2005)	2D spiral (2D surface micromachining)
196.	[189]	(Kowase et al. 2005)	2D spiral (2D surface micromachining)
197.	[190]	(Estibals et al. 2005)	Stacked spiral (2D surface micromachining)
198.	[191]	(Musunuri and Chapman 2005)	2D spiral (2D surface micromachining)
199.	[192]	(Sun et al. 2005)	2D V-groove Inductor (2D surface micromachining)
200.	[193]	(Gao et al. 2005)	3D solenoid (surface micromachining), UV-LIGA
201.	[194]	(Mathúna et al. 2005)	Review on Magnetics for Power Supply on Chip
202.	[195]	(Yoon and Allen 2005)	3D solenoid (surface micromachining),
203.	[196]	(Wang et al. 2005)	2D racetrack (2D surface micromachining)
204.	[194]	(Mathúna et al. 2005)	Review on Magnetics for Power Supply on Chip

205.	[197]	(Gao et al. 2006)	3D solenoid (surface micromachining)
206.	[198]	(Flynn et al. 2006)	3D solenoid (surface micromachining)
207.	[199]	(Flynnt et al. 2006)	3D solenoid (surface micromachining)
208.	[200]	(Hua et al. 2006)	3D solenoid (surface micromachining)
209.	[201]	(Orlando et al. 2006)	3D toroidal (surface micromachining)
210.	[198]	(Flynn et al. 2006)	3D solenoid (surface micromachining)
211.	[202]	(Lei et al. 2007)	3D solenoid (surface micromachining)
212.	[203]	(Galle et al. 2007)	2D spiral (2D surface micromachining)
213.	[204]	(Shen et al. 2007)	Others: wire-bond
214.	[205]	(Wang et al. 2007)	2D racetrack (2D surface micromachining)
215.	[206]	(Wang et al. 2007)	2D in-sub spiral inductor (TSV)
216.	[207]	(Lu et al. 2008)	Others: wire-bond
217.	[208]	(Mastouli et al. 2008)	2D racetrack (2D surface micromachining)
218.	[209]	(Zhou et al. 2008)	3D solenoid (surface micromachining)
219.	[210]	(Steyaert 2008)	Others: wire-bond
220.	[211]	(O'Donnell et al. 2008)	2D racetrack (2D surface micromachining)
221.	[209]	(Zhou et al. 2008)	3D solenoid (3D surface micromachining)
222.	[212]	(Wang et al. 2008)	3D in-sub toroidal (TSV)
223.	[213]	(Lee et al. 2008)	3D solenoid (3D surface micromachining)
224.	[207]	(Lu et al. 2008)	Others: wire-bond
225.	[214]	(Wang et al. 2008)	2D racetrack (2D surface micromachining)
226.	[215]	(Sullivan 2009)	Review on Magnetics for Power Supply on Chip
227.	[216]	(Martin et al. 2009)	2D spiral (2D surface micromachining)
228.	[217]	(Kim et al. 2009)	2D spiral (2D surface micromachining)
229.	[218]	(Bae et al. 2009)	2D spiral (2D surface micromachining)
230.	[219]	(O'Donnell et al. 2009)	2D racetrack (2D surface micromachining)
231.	[220]	(Flynn and Desmulliez 2009)	Others: Flip-Chip
232.	[221]	(O'Donnell et al. 2009)	2D racetrack (2D surface micromachining)
233.	[222]	(Gardner et al. 2009)	Review on on-chip Magnetics
234.	[223]	(Meyer et al. 2010)	Stacked spiral (2D surface micromachining)
235.	[224]	(O'Donnell et al. 2010)	2D racetrack (2D surface micromachining)
236.	[225]	(Lee et al. 2010)	2D racetrack (2D surface micromachining)
237.	[226]	(Rodgers 2010)	2D racetrack (2D surface micromachining)
238.	[227]	(Wang et al. 2010)	2D in-sub spiral inductor (TSV)
239.	[228]	(Jia et al. 2011)	Others: wire-bond
240.	[229]	(Wang et al. 2011)	2D in-sub spiral inductor (TSV)
241.	[230]	(Wu and Sin 2011)	2D in-sub spiral inductor (TSV)
242.	[231]	(Wang et al. 2011)	2D in-sub spiral inductor (TSV)
243.	[232]	(Jaejin Lee et al. 2011)	2D spiral (2D surface micromachining)
244.	[233]	(Xue et al. 2011)	Stacked spiral (2D surface micromachining)
245.	[234]	(Ito et al. 2011)	2D spiral (2D surface micromachining)
246.	[74]	(Artillan et al. 2011)	2D spiral (2D surface micromachining)
247.	[235]	(Rabia et al. 2011)	2D spiral (2D surface micromachining)
248.	[236]	(Meere et al. 2011)	Review: Air-core vs. magnetic-core inductor

249.	[237]	(Li 2012)	2D spiral (2D surface micromachining)
250.	[238]	(Qiu and Sullivan 2012a)	2D racetrack (2D surface micromachining)
251.	[239]	(Qiu and Sullivan 2012b)	3D toroidal (surface micromachining)
252.	[240]	(Li et al. 2012)	3D in-sub toroidal (TSV)
253.	[241]	(Yu et al. 2012)	3D in-sub toroidal (substrate-embedded), UV-LIGA
254.	[242]	(Fang et al. 2012)	2D in-sub spiral inductor (substrate-embedded)
255.	[243]	(Wu and Sin 2012)	2D in-sub spiral inductor (substrate-embedded)
256.	[244]	(Yu et al. 2012)	3D in-sub toroidal (substrate-embedded), UV-LIGA
257.	[245]	(Kriga et al. 2012)	2D spiral (2D surface micromachining)
258.	[246]	(Harburg et al. 2012)	2D racetrack (2D surface micromachining)
259.	[247]	(Harburg et al. 2012)	2D racetrack (2D surface micromachining)
260.	[238]	(Qiu and Sullivan 2012)	2D racetrack (2D surface micromachining)
261.	[237]	(Li 2012)	2D spiral (2D surface micromachining)
262.	[248]	(Yu et al. 2013)	3D in-sub toroidal (TSV)
263.	[249]	(Harburg et al. 2013)	2D racetrack (2D surface micromachining)
264.	[250]	(Peng et al. 2013)	2D in-sub spiral inductor (TSV)
265.	[251]	(Wu et al. 2013)	2D in-sub spiral inductor (TSV)
266.	[252]	(Jamieson et al. 2013)	2D racetrack (2D surface micromachining)
267.	[253]	(Wang et al. 2013)	2D racetrack (2D surface micromachining)
268.	[254]	(Sugawa et al. 2013)	2D spiral (2D surface micromachining)
269.	[255]	(Sturcken et al. 2013)	2D racetrack (2D surface micromachining)
270.	[86]	(Moazenzadeh et al. 2013)	Others: wire-bond
271.	[256]	(Yao et al. 2013)	2D V-groove Inductor (2D surface micromachining)
272.	[257]	(Kim et al. 2013)	3D toroidal (3D surface micromachining), UV-LIGA
273.	[258]	(Kim et al. 2013)	3D toroidal (3D surface micromachining), UV-LIGA
274.	[259]	(Araghchini et al. 2014)	3D in-sub toroidal (TSV), UV-LIGA
275.	[260]	(Fang et al. 2014)	3D in-sub toroidal (TSV)
276.	[261]	(Tida et al. 2014)	3D in-sub solenoid (TSV)
277.	[262]	(Nguyen et al. 2014)	3D solenoid (3D surface micromachining)
278.	[263]	(Peng et al. 2014)	3D in-sub solenoid (TSV)
279.	[264]	(Anthony et al. 2014)	2D racetrack (2D surface micromachining)
280.	[265]	(Kim et al. 2015)	3D toroidal (3D surface micromachining)
281.	[266]	(Macrelli et al. 2015)	Others: wire-bond
282.	[267]	(Yu and Allen 2015)	3D in-sub toroidal (substrate-embedded), UV-LIGA
283.	[268]	(Ostfeld et al. 2015)	Others: Printing
284.	[269]	(Tien et al. 2015)	2D in-sub spiral inductor (TSV)
285.	[270]	(Tida et al. 2015)	3D in-sub solenoid (TSV)
286.	[271]	(Fang et al. 2015)	2D in-sub spiral inductor (TSV)
287.	[272]	(Feeney et al. 2015)	2D racetrack (2D surface micromachining)
288.	[273]	(Feeney et al. 2015)	2D racetrack (2D surface micromachining)
289.	[274]	(Kim et al. 2015)	3D solenoid (3D surface micromachining)
290.	[275]	(Sturcken et al. 2015)	3D solenoid (3D surface micromachining)
291.	[276]	(Pavlovic et al. 2015)	Stacked 2D racetrack (2D surface micromachining)
292.	[277]	(Mondal et al. 2015)	3D in-sub solenoid (TSV)

293.	[278]	(Anthony et al. 2016)	2D racetrack (2D surface micromachining)
294.	[279]	(Fang et al. 2016)	2D in-sub spiral inductor (TSV)
295.	[280]	(Mishra et al. 2016)	3D solenoid (3D surface micromachining)
296.	[281]	(Anthony et al. 2016)	2D racetrack (2D surface micromachining)
297.	[278]	(Anthony et al. 2016)	2D racetrack (2D surface micromachining)
298.	[282]	(Anthony et al. 2016)	2D racetrack (2D surface micromachining)
299.	[283]	(He et al. 2016)	3D solenoid (3D surface micromachining)
300.	[284]	(Lee et al. 2016)	2D spiral (2D surface micromachining)
301.	[285]	(Dinulovic et al. 2016)	3D solenoid (3D surface micromachining)
302.	[286]	(Krishnamurthy et al. 2017)	3D solenoid (3D surface micromachining)
303.	[287]	(Li et al. 2017)	2D in-sub spiral inductor (TSV)
304.	[288]	(Muthukumaraswamy et al. 2017)	3D solenoid (3D surface micromachining)
305.	[289]	(Femandez et al. 2017)	2D racetrack (2D surface micromachining)
306.	[290]	(Ding et al. 2018)	2D in-sub spiral inductor (TSV)
307.	[291]	(Syed Mohammed et al. 2018)	2D in-sub spiral inductor (substrate-embedded)
308.	[292]	(Dinulovic et al. 2018)	3D solenoid (3D surface micromachining)
309.	[293]	(Chen et al. 2018)	3D in-sub solenoid (TSV)
310.	[294]	(Bellaredj et al., 2018)	2D spiral (2D surface micromachining)
311.	[295]	(Li et al. 2018)	3D in-sub solenoid (TSV)
312.	[296]	(Le et al. 2018)	3D in-sub solenoid (TSV)/ toroidal
313.	[297]	(Harburg et al. 2018)	2D racetrack (2D surface micromachining)
314.	[298]	(Wu et al. 2018)	2D in-sub spiral inductor (TSV)
315.	[299]	(Chan et al. 2018)	3D in-sub solenoid (TSV)
316.	[300]	(Le et al. 2018)	3D in-sub solenoid (TSV)/ toroidal
317.	[301]	(Beilliard et al., 2018)	2D racetrack (2D surface micromachining)
318.	[302]	(Hamid et al., 2018)	2D racetrack (2D surface micromachining)
319.	[303]	(Krishnamurthy et al. 2018)	3D solenoid (3D surface micromachining)
320.	[304]	(Le et al. 2019)	3D in-sub solenoid (TSV)
321.	[305]	(Fukuoka et al. 2019)	2D spiral (2D surface micromachining)
322.	[306]	(Hsieh et al. 2019)	3D in-sub solenoid (TSV)/ toroidal
323.	[307]	(Bellaredj et al. 2019)	3D solenoid (3D surface micromachining)
324.	[308]	(Renz et al. 2019)	2D spiral (2D surface micromachining)
325.	[309]	(Xu et al. 2019)	3D in-sub solenoid (TSV)
326.	[307]	(Bellaredj et al. 2019)	3D solenoid (3D surface micromachining)
327.	[310]	(Xu et al. 2019)	2D spiral (2D surface micromachining)
328.	[311]	(Ding et al. 2019)	2D spiral (2D surface micromachining), Others: Flip-Chip,
329.	[312]	(Singh et al. 2019)	3D solenoid (3D surface micromachining)
330.	[313]	(Xu et al. 2019)	3D in-sub solenoid (TSV)
331.	[314]	(Akiyama et al. 2019)	2D spiral (2D surface micromachining), magnetic substrate
332.	[315]	(Sun et al. 2020)	3D in-sub solenoid (TSV), organic substrate
333.	[316]	(Huang et al. 2020)	3D in-sub solenoid (TSV)
334.	[317]	(Pavlovic et al. 2020)	3D solenoid (3D surface micromachining)

III. Biomedical and Neurotechnology Applications

No.	Ref.	Author Year	Fabrication Technology
335.	[37]	(Massin et al. 2002)	2D spiral (2D surface micromachining)
336.	[318]	(Gimi et al. 2003)	2D spiral (2D surface micromachining)
337.	[319]	(Syms et al. 2005)	Planar coil (2D surface micromachining)
338.	[320]	(Mona J. K. Klein et al. 2007)	3D In-Sub solenoid, MRI
339.	[321]	(Klein et al. 2008)	3D In-Sub solenoid, MRI
340.	[322]	(Wu et al. 2012)	2D in-sub spiral inductor (TSV), Brain-Machine Interface
341.	[81]	(Xue et al. 2012)	Stacked spiral (2D surface micromachining), implantable
342.	[323]	(Wu et al. 2013)	2D in-sub spiral inductor (TSV) , implantable
343.	[324]	(Coskun et al. 2013)	2D spiral (2D surface micromachining), implantable pressure sensor
344.	[325]	(Wu et al. 2014)	2D spiral (2D surface micromachining), NMR probe
345.	[326]	(Fratila et al. 2014)	2D spiral (2D surface micromachining), NMR probe
346.	[327]	(Olivo et al. 2014)	2D spiral (2D surface micromachining), implantable biosensors
347.	[328]	(Sun et al. 2014)	2D spiral (2D surface micromachining), implantable
348.	[329]	(Lee et al. 2016)	2D planar (2D surface micromachining), intracortical magnetic stimulation
349.	[330]	(Sun et al. 2017)	2D spiral (2D surface micromachining), microbeads trapping
350.	[331]	(Cardoso et al. 2017)	Review: planar inductors for biomedical applications
351.	[332]	(Likhite et al. 2017)	2D spiral (2D surface micromachining), neural probe
352.	[333]	(Zhang and Nguyen 2017)	Review: Magnetics for microfluidics
353.	[334]	(Feng et al. 2018)	2D spiral (2D surface micromachining), magnetic microbeads
354.	[335]	(Rizou and Prodromakis 2018)	2D spiral (2D surface micromachining), magnetic stimulation
355.	[336]	(Rizou and Prodromakis 2018)	2D spiral (2D surface micromachining), non-invasive neuro-stimulation
356.	[337]	(Shadid and Noghanian 2018)	Review on Inductors for Wireless Power Transfer of Biomedical Devices
357.	[338]	(Dupré et al. 2019)	Review: planar inductors for NMR
358.	[339]	(Lee et al. 2019)	2D planar (2D surface micromachining), intracortical magnetic stimulation
359.	[340]	(Mansour et al. 2019)	2D spiral (2D surface micromachining), implantable NMR coil
360.	[341]	(Zhi et al. 2019)	2D planar (2D surface micromachining), bio-target sorting
361.	[342]	(Lefebvre et al. 2020)	2D spiral (2D surface micromachining), magnetic microbeads trapping
362.	[343]	(Handwerker et al. 2020)	2D spiral (2D surface micromachining), implantable NMR coil

IV. Wireless and sensors:

No.	Ref.	Author Year	Fabrication Technology
363.	[344]	(Kawahito et al. 1994)	3D solenoid (surface micromachining)
364.	[345]	(Woytasik et al. 2006)	3D solenoid (surface micromachining)
365.	[322]	(Wu et al. 2012)	2D in-sub spiral inductor (TSV)
366.	[81]	(Xue et al. 2012)	Stacked spiral (2D surface micromachining), intraocular pressure sensor
367.	[323]	(Wu et al. 2013)	2D in-sub spiral inductor (TSV) , wireless power transfer

368.	[85]	(Mohamed Ali et al. 2013)	2D spiral (2D surface micromachining), wireless SMA actuators
369.	[324]	(Coskun et al. 2013)	2D spiral (2D surface micromachining), implantable pressure sensor
370.	[327]	(Olivo et al. 2014)	2D spiral (2D surface micromachining), wireless power transfer
371.	[346]	(Mohamed Ali et al. 2014)	2D spiral (2D surface micromachining), wireless displacement sensing
372.	[328]	(Sun et al. 2014)	2D spiral (2D surface micromachining), wireless power transfer
373.	[347]	(Wu et al. 2015b)	2D in-sub spiral inductor (TSV), wireless power transfer
374.	[348]	(Zargham 2015)	2D spiral (2D surface micromachining), wireless power transfer
375.	[92]	(Wu et al. 2015)	Others: Printing, wireless sensors
376.	[349]	(Wu et al. 2015a)	2D in-sub spiral inductor (TSV), Signal Isolation
377.	[350]	(Kim et al. 2015)	Others: printing, wireless communication
378.	[95]	(Huang et al. 2015)	Others: self-assembly rolled-up, RFIC
379.	[351]	(Jun et al. 2015)	2D spiral (2D surface micromachining), wireless thin-film transistor
380.	[352]	(Xin Yu et al. 2015)	Others: self-assembly rolled-up, RFIC
381.	[353]	(Wu et al. 2016)	2D in-sub spiral inductor (TSV), wireless power transfer
382.	[97]	(Zhang et al. 2016)	2D in-sub spiral inductor (TSV) , RFIC
383.	[106]	(Wang et al. 2017)	2D spiral (2D surface micromachining), RFIC
384.	[354]	(Baldwin et al. 2017)	2D spiral (2D surface micromachining), wireless communication
385.	[332]	(Likhite et al. 2017)	2D spiral (2D surface micromachining), wireless power coupling
386.	[355]	(Wu et al. 2017)	2D in-sub spiral inductor (TSV), wireless power transfer
387.	[356]	(Pardue et al. 2018)	2D spiral (2D surface micromachining), wireless energy harvesting
388.	[119]	(Huang et al. 2018)	Others: self-assembly rolled-up, wireless communication
389.	[334]	(Feng et al. 2018)	2D spiral (2D surface micromachining), magnetic sensor
390.	[357]	(Karnaushenko et al., 2018)	Others: self-assembly rolled-up, magnetic sensor
391.	[358]	(Tang et al. 2018)	2D planar (2D surface micromachining), displacement sensor
392.	[359]	(Pardue et al. 2019)	2D spiral (2D surface micromachining), wireless power transfer
393.	[134]	(Ha et al. 2019)	Others: Printing, wireless communication
394.	[360]	(Komaragiri and K.J 2019)	2D planar (2D surface micromachining), biosensing
395.	[361]	(Hara and Yamaguchi 2020)	2D planar (2D surface micromachining), IoT sensor
396.	[142]	(Dinulovic et al. 2020)	3D solenoid (3D surface micromachining), signal applications
397.	[362]	(Lin et al. 2020)	2D planar (2D surface micromachining), fingerprint sensor

Bibliography

[1] R. G. M. Nhat M. Nguyen, "Si IC-Compatible Inductors and LC Passive Filters," *IEEE J. Solid-State Circuits*, vol. 25, no. 9036489, pp. 1028–1031, 1990.

[2] H. Matsuki, N. Fujii, K. Shirakawa, J. Toriu, and K. Murakami, "Magnetic-Multi-Turn Planar Coil Inductor," *IEEE Trans. Magn.*, vol. 27, no. 6, pp. 5438–5440, 1991.

[3] C. H. Ahn and M. G. Allen, "Fully integrated micromagnetic actuator with a multilevel meander magnetic core," *Tech. Dig. IEEE Solid-State Sens. Actuator Work.*, no. July 1992, pp. 16–18, 1992.

[4] C. H. Ahn and M. G. Allen, "A Fully Integrated Surface Micromachined Magnetic Microactuator with a Multilevel Meander Magnetic Core," *J. Microelectromechanical Syst.*, vol. 2, no. 1, pp. 15–22, 1993.

[5] C. H. Ahn and M. G. Allen, "A New toroidal-meander type integrated inductor with a multilevel meander magnetic core," *IEEE Trans. Magn.*, vol. 30, no. 1, pp. 73–79, 1994.

[6] A. C. Reyes, S. J. Dorn, H. Patterson, S. M. El-Ghazaly, D. K. Schroder, and M. Dydyk, "Coplanar Waveguides and Microwave Inductors on Silicon Substrates," *IEEE Trans. Microw. Theory Tech.*, vol. 43, no. 9, pp. 2016–2022, 1995.

[7] J. R. Long and M. A. Copeland, "Modeling of monolithic inductors and transformers for silicon RFIC design," in *1995 Int. Top. Symp.*, 1995, pp. 129–134.

[8] A. Rofougaran, J. Rael, M. Rofougaran, and A. Abidi, "A 900MHz CMOS LC-Oscillator with Quadrature Outputs," *Analog Tech.*, vol. 14, no. 5, pp. 392–393, 1996.

[9] A. Rofougaran *et al.*, "900 MHz CMOS frequency-hopped spread-spectrum RF transmitter IC," *Proc. Cust. Integr. Circuits Conf.*, pp. 209–212, 1996.

[10] Y. Sun, F. E. van Vliet, J. L. Tauritz, and R. G. F. Baets, "Monolithic narrow-band active inductors using suspended membrane passive components on silicon substrate," *Work. High Perform. Electron Devices Microw. Optoelectron. Appl. EDMO*, no. November, pp. 79–84, 1996.

[11] L. Zu *et al.*, "High Q-factor inductors integrated on MCM Si substrates," *IEEE Trans. Components Packag. Manuf. Technol. Part B*, vol. 19, no. 3, pp. 635–642, 1996.

[12] C. M. Nam and Y. S. Kwon, "High-performance planar inductor on thick oxidized porous silicon (OPS) substrate," *IEEE Microw. Guid. Wave Lett.*, vol. 7, no. 8, pp. 236–238, 1997.

[13] J. Y. P. and M. G. Allen, "High current integrated microinductors and microtransformers using low temperature fabrication processes," *Microelectron. Int.*, vol. 14, no. 3, 1997.

[14] C. H. Ahn and M. G. Allen, "Micromachined planar inductors on silicon wafers for MEMS applications," *IEEE Trans. Ind. Electron.*, 1998.

[15] A. Rofougaran, J. Y. C. Chang, M. Rofougaran, and A. A. Abidi, "A 1 GHz CMOS RF front-end IC for a direct-conversion wireless receiver," *Integr. Circuits Wirel. Commun.*, vol. 31, no. 7, pp. 248–257, 1998.

[16] R. P. Ribas, J. Lescot, J. L. Leclercq, J. M. Karam, and F. Ndagijimana, "Monolithic micromachined planar spiral transformer," *Tech. Dig. - GaAs IC Symp. (Gallium Arsenide Integr. Circuit)*, pp. 255–258, 1998.

[17] J. B. Yoon, C. H. Han, E. Yoon, and C. K. Kim, "Monolithic fabrication of electroplated solenoid inductors using three-dimensional photolithography of a thick photoresist," *Japanese J. Appl. Physics, Part 1 Regul. Pap. Short Notes Rev. Pap.*, vol. 37, no. 12 B, pp. 7081–7085, 1998.

[18] Y. J. Kim and M. G. Allen, "Surface micromachined solenoid inductors for high frequency applications," *IEEE Trans. components, Packag. Manuf. Technol. Part C. Manuf.*, vol. 21, no. 1, pp. 26–33, 1998.

[19] J.-B. Yoon, C.-H. Han, E. Yoon, and C.-K. Kim, "Novel and high-yield fabrication of electroplated 3D micro-coils for MEMS and microelectronics," *Micromach. Microfabr. Process Technol. IV*, vol. 3511, pp. 233–240, 1998.

[20] M. Park, S. Lee, C. S. Kim, H. K. Yu, and K. S. Nam, "The detailed analysis of high Q CMOS-compatible microwave spiral inductors in silicon technology," *IEEE Trans. Electron Devices*, vol. 45, no. 9, pp. 1953–1959, 1998.

[21] R. P. Ribas, J. Lescot, J. L. Leclercq, N. Bennouri, J. M. Karam, and B. Courtois, "Micromachined planar spiral inductor in standard GaAs HEMT MMIC technology," *IEEE Electron Device Lett.*, vol. 19, no. 8, pp. 285–287, 1998.

[22] D. H. Young, V. Malba, J. J. Ou, A. Bernhardt, and B. E. Boser, "A low-noise RF voltage-controlled oscillator using on-chip high-Q three dimensional coil inductor and micromachined variable capacitor," *Solid-State Sens. Actuator Work.*, vol. pp, pp. 128–131, 1998.

[23] J. Yoon, B. Kim, C. Han, E. Yoon, and K. Lee, "High-Performance Electroplated Solenoid-Type Integrated Inductor (SI2) for," in *Int. Electron Devices Meet.*, 1998, vol. i, pp. 544–547.

[24] L. Fan, R. T. Chen, A. Nespola, and M. C. Wu, "Universal MEMS platforms for passive RF components: Suspended inductors and variable capacitors," in *Proc. IEEE Micro Electro Mech. Syst.*, 1998, pp. 29–33.

[25] Y. J. Kim and M. G. Allen, "Integrated solenoid-type inductors for high frequency applications and their characteristics," *Proc. - Electron. Components Technol. Conf.*, vol. Part F1334, pp. 1247–1252, 1998.

[26] T. M. Liakopoulos and C. H. Ahn, "3-D Microfabricated Toroidal Planar Inductors with Different Magnetic Core Schemes for MEMS and Power Electronic Applications," *IEEE Trans. Magn.*, vol. 35, no. 5 PART 2, pp. 3679–3681, 1999.

[27] J. Y. Park and M. G. Allen, "New micromachind inductors on silicon substrates," *Dig. Intermag Conf.*, vol. 35, no. 5, pp. 3547–3549, 1999.

[28] J. B. Yoon, C. H. Han, E. Yoon, and C. K. Kim, “Monolithic high-Q overhang inductors fabricated on silicon and glass substrates,” *Tech. Dig. - Int. Electron Devices Meet.*, pp. 753–756, 1999.

[29] M. Yamaguchi *et al.*, “Microfabrication and characteristics of magnetic thin-film inductors in the ultrahigh frequency region,” *J. Appl. Phys.*, vol. 85, no. 11, pp. 7919–7922, 1999.

[30] J.-B. Yoon and E. Y. and C.-K. K. , Chul-Hi Han, “High-performance three-dimensional on-chip inductors fabricated by novel micromachining technology for RF MMIC,” in *IEEE MTT-S Int. Microw. Symp. Dig.*, 1999, pp. 1523–1526.

[31] C. Patrick Yue and S. Simon Wong, “Physical modeling of spiral inductors on silicon,” *IEEE Trans. Electron Devices*, vol. 47, no. 3, pp. 560–568, 2000.

[32] J. M. López-Villegas, J. Samitier, C. Cane, P. Losantos, and J. Bausells, “Improvementof the quality factor of rf integrated inductors by layout optimization,” *IEEE Trans. Microw. Theory Tech.*, vol. 48, no. 1, pp. 76–83, 2000.

[33] N. Chomnawang and J.-B. Lee, “On-chip 3D air-core microinductor for high-frequency applications using deformation of sacrificial polymer,” *Smart Struct. Mater. 2001 Smart Electron. MEMS*, vol. 4334, p. 54, 2001.

[34] M. Yamaguchi, M. Baba, and K. I. Arai, “Sandwich type ferromagnetic RF integrated inductor,” *IEEE MTT-S Int. Microw. Symp. Dig.*, vol. 3, pp. 185–188, 2001.

[35] Y. C. Liang, W. Zeng, P. H. Ong, Z. Gao, J. Cai, and N. Balasubramanian, “A concise process technology for 3-D suspended radio frequency micro-inductors on silicon substrate,” *IEEE Electron Device Lett.*, vol. 23, no. 12, pp. 700–703, 2002.

[36] M. Gel, S. Takeuchi, and I. Shimoyama, “Fabrication method for out-of-plane, micro-coil by surface micromachining,” *Sensors Actuators, A Phys.*, vol. 97–98, pp. 702–708, 2002.

[37] C. Massin, G. Boero, F. Vincent, J. Abenhaim, P. A. Besse, and R. S. Popovic, “High-Q factor RF planar microcoils for micro-scale NMR spectroscopy,” *Sensors Actuators, A Phys.*, vol. 97–98, no. November 2001, pp. 280–288, 2002.

[38] M. Brunet, T. O’Donnell, J. O’Brien, P. McCloskey, and S. C. Ó. Mathuna, “Thick photoresist development for the fabrication of high aspect ratio magnetic coils,” *J. Micromechanics Microengineering*, vol. 12, no. 4, pp. 444–449, 2002.

[39] H. Lakdawala, X. Zhu, H. Luo, S. Santhanam, L. Richard Carley, and G. K. Fedder, “Micromachined high-Q inductors in a 0.18-µm copper interconnect low-K dielectric CMOS process,” *IEEE J. Solid-State Circuits*, vol. 37, no. 3, pp. 394–403, 2002.

[40] J. N. Burghartz and B. Rejaei, “On the design of RF spiral inductors on silicon,” *IEEE Trans. Electron Devices*, vol. 50, no. 3, pp. 718–729, 2003.

[41] Y. Cao *et al.*, “Frequency-independent equivalent-circuit model for on-chip spiral inductors,” *IEEE J. Solid-State Circuits*, vol. 38, no. 3, pp. 419–426, 2003.

[42] J. Zou, C. Liu, D. R. Trainor, J. Chen, J. E. Schutt-Ainé, and P. L. Chapman, “Development of three-dimensional inductors using plastic deformation magnetic assembly (PDMA),” *IEEE Trans. Microw. Theory Tech.*, vol. 51, no. 4 I, pp. 1067–1075, 2003.

[43] M. G. Allen, “MEMS technology for the fabrication of RF magnetic components,” *Intermag 2003 - Progr. 2003 IEEE Int. Magn. Conf.*, vol. 39, no. 5, pp. 3073–3078, 2003.

[44] C. L. Chua, D. K. Fork, K. Van Schuylenbergh, and J. P. Lu, “Out-of-plane high-Q inductors on low-resistance silicon,” *J. Microelectromechanical Syst.*, vol. 12, no. 6, pp. 989–995, 2003.

[45] J. Chen and J. Liou, “On-chip spiral inductors for RF applications: an overview,” *J. Semicond. Technol. Sci.*, vol. 4, no. 3, pp. 149–167, 2004.

[46] X. N. Wang, X. L. Zhao, Y. Zhou, X. H. Dai, and B. C. Cai, “Fabrication and performance of a novel suspended RF spiral inductor,” *IEEE Trans. Electron Devices*, vol. 51, no. 5, pp. 814–816, 2004.

[47] T. Pan, A. Baldi, R. F. Drayton, and B. Ziaie, “Fabrication and modeling of silicon-embedded high Q inductors,” in *17th IEEE Int. Conf. Micro Electro Mech. Syst.*, 2004, pp. 809–812.

[48] A. M. I. M.B. Pisani, C. Hibert, D. Bouvet, P. Beaud, “Copper/polyimide fabrication process for above-IC integration of high quality factor inductors,” *Microelectron. Eng.*, vol. 73–74, pp. 474–479, 2004.

[49] K. L. Scott, T. Hirano, H. Yang, H. Singh, R. T. Howe, and A. M. Niknejad, “High-performance inductors using capillary based fluidic self-assembly,” *J. Microelectromechanical Syst.*, vol. 13, no. 2, pp. 300–309, 2004.

[50] Y. Zhuang, M. Vroubel, B. Rejaei, J. N. Burghartz, and K. Attenborough, “Magnetic properties of electroplated nano/microgranular NiFe thin films for rf application,” *J. Appl. Phys.*, vol. 97, no. 10, pp. 10–13, 2005.

[51] M. Raieszadeh, P. Monajemi, S.-W. Yoon, J. Lash-ur, and F. Ayuzi, “High-Q integrated inductors on trenched silicon islands,” in *18th IEEE Int. Conf. Micro Electro Mech. Syst.*, 2005, pp. 199–202.

[52] M. Rais-Zadeh and F. Ayazi, “Characterization of high-Q spiral inductors on thick insulator-on-silicon,” *J. Micromechanics Microengineering*, vol. 15, no. 11, pp. 2105–2112, 2005.

[53] Y. K. Yoon, J. W. Park, and M. G. Allen, “Polymer-core conductor approaches for RF MEMS,” *J. Microelectromechanical Syst.*, vol. 14, no. 5, pp. 886–894, 2005.

[54] E. G. Turitsyna and S. Webb, “High performance MEMS inductors fabricated on localised and planar thick SiO2 layer,” *Electron. Lett.*, vol. 41, no. 2, pp. 40–41, 2005.

[55] P. Dixit and J. Miao, “Aspect-Ratio-Dependent Copper Electrodeposition Technique for Very High Aspect-Ratio Through-Hole Plating,” *J. Electrochem. Soc.*, vol. 153, no. 6, p. G552, 2006.

[56] C. Lei *et al.*, “Fabrication of 3D MEMS toroidal microinductor for high temperature application,” *Microelectronics J.*, vol. 37, no. 11, pp. 1347–1351, 2006.

[57] K. Seemann, H. Leiste, and V. Bekker, “A new generation of CMOS-compatible high frequency micro-inductors with ferromagnetic cores: Theory, fabrication and characterisation,” *J. Magn. Magn. Mater.*, vol. 302, no. 2, pp. 321–326, 2006.

[58] D. M. Fang, X. N. Wang, Y. Zhou, and X. L. Zhao, “Fabrication and performance of a micromachined 3-D solenoid inductor,” *Microelectronics J.*, vol. 37, no. 9, pp. 948–951, 2006.

[59] D. M. Fang, Y. Zhou, X. N. Wang, and X. L. Zhao, “Surface micromachined high-performance RF MEMS inductors,” *Microsyst. Technol.*, vol. 13, no. 1, pp. 79–83, 2007.

[60] P. J. Bell *et al.*, “Flip-Chip-Assembled Air-Suspended Inductors,” *IEEE Trans. Adv. Packag.*, vol. 30, no. 1, pp. 148–154, 2007.

[61] H. Lu, B. Pillans, J. C. Lee, and J. B. Lee, “High aspect ratio air core solenoid inductors using an improved UV-LIGA process with contrast enhancement material,” *Microsyst. Technol.*, vol. 13, no. 3–4, pp. 237–243, 2007.

[62] C. M. Tai and C. N. Liao, “Multilevel suspended thin-film inductors on silicon wafers,” *IEEE Trans. Electron Devices*, vol. 54, no. 6, pp. 1510–1514, 2007.

[63] Y. Zhuang, M. Vroubel, B. Rejaei, and J. N. Burghartz, “Integrated RF inductors with micro-patterned NiFe core,” *Solid. State. Electron.*, vol. 51, no. 3, pp. 405–413, 2007.

[64] L. Gu and X. Li, “High-Q solenoid inductors with a CMOS-compatible concave-suspending MEMS process,” *J. Microelectromechanical Syst.*, vol. 16, no. 5, pp. 1162–1172, 2007.

[65] H. Mekaru *et al.*, “Fabrication of a spiral microcoil using a 3D-LIGA process,” *Microsyst. Technol.*, vol. 13, no. 3–4, pp. 393–402, 2007.

[66] I. Zine-El-Abidine and M. Okoniewski, “CMOS-Compatible Micromachined Toroid and Solenoid Inductors With High Q-Factors,” *IEEE Electron Device Lett.*, vol. 28, no. 3, pp. 226–228, 2007.

[67] C. Xue, F. Yao, B. Cheng, and Q. Wang, “Effect of the silicon substrate structure on chip spiral inductor,” *Front. Electr. Electron. Eng. China*, vol. 3, no. 1, pp. 110–115, 2008.

[68] M. C. Hsieh, D. K. Jair, and C. S. Lin, “Design and fabrication of the suspended high-Q spiral inductors with X-beams,” *Microsyst. Technol.*, vol. 14, no. 7, pp. 903–907, 2008.

[69] D. S. W. Park, Y. Jeong, J. B. Lee, and S. Jung, “Chip-level integration of RF MEMS on-chip inductors using UV-LIGA technique,” *Microsyst. Technol.*, vol. 14, no. 9–11, pp. 1429–1438, 2008.

[70] J. C. Wu and M. E. Zaghloul, “CMOS micromachined inductors with structure supports for RF mixer matching networks,” *IEEE Electron Device Lett.*, vol. 29, no. 11, pp. 1209–1211, 2008.

[71] J. Lee, S. Park, H. C. Kim, and K. Chun, “Substrates and dimension dependence of MEMS inductors,” *J. Micromechanics Microengineering*, vol. 19, no. 8, 2009.

[72] K. Kratt, V. Badilita, T. Burger, J. G. Korvink, and U. Wallrabe, “A fully MEMS-compatible process for 3D high aspect ratio micro coils obtained with an automatic wire bonder,” *J. Micromechanics Microengineering*, vol. 20, no. 1, 2010.

[73] Q. Ding, Y. Shi, and X. Li, “Quality factor enhancement of spiral inductors with patterned trench isolation,” *ICSICT-2010 - 2010 10th IEEE Int. Conf. Solid-State Integr. Circuit Technol. Proc.*, pp. 731–733, 2010.

[74] P. Artillan *et al.*, “Integrated LC filter on silicon for dc-dc converter applications,” *IEEE Trans. Power Electron.*, vol. 26, no. 8, pp. 2319–2325, 2011.

[75] C. Pacurar, V. Topa, A. Racasan, and C. Munteanu, “Inductance calculation and layout optimization for planar spiral inductors,” *Proc. Int. Conf. Optim. Electr. Electron. Equipment, OPTIM*, pp. 225–232, 2012.

[76] P. Shen, W. Zhang, L. Huang, D. Jin, and H. Xie, “Improving the quality factor of an RF spiral inductor with non-uniform metal width and non-uniform coil spacing,” *J. Semicond.*, vol. 32, no. 6, 2011.

[77] M. Z. Yang, C. L. Dai, and J. Y. Hong, “Manufacture and characterization of high Q-factor inductors based on CMOS-MEMS techniques,” *Sensors*, vol. 11, no. 10, pp. 9798–9806, 2011.

[78] W. A. Vitale, M. Fernandez-Bolanos, and A. M. Ionescu, “High-Q 3D embedded inductors using TSV for RF MEMS tunable bandpass filters (4.65-6.8 GHz),” *Eur. Microw. Week 2012 “sp. Microwaves”, EuMW 2012, Conf. Proc. - 7th Eur. Microw. Integr. Circuits Conf. EuMIC 2012*, no. February 2015, pp. 822–825, 2012.

[79] C. D. Meyer, S. S. Bedair, B. C. Morgan, and D. P. Arnold, “Influence of layer thickness on the performance of stacked thick-film copper air-core power inductors,” *IEEE Trans. Magn.*, vol. 48, no. 11, pp. 4436–4439, 2012.

[80] J. Zhan *et al.*, “Stacked-spiral RF inductor with vertical nano-powder magnetic core in CMOS,” *IEEE Microw. Wirel. Components Lett.*, vol. 22, no. 1, pp. 29–31, 2012.

[81] N. Xue, S. P. Chang, and J. B. Lee, “A SU-8-based microfabricated implantable inductively coupled passive RF wireless intraocular pressure sensor,” *J. Microelectromechanical Syst.*, vol. 21, no. 6, pp. 1338–1346, 2012.

[82] H. Y. Li *et al.*, “Three-dimensional solenoids realized via high-density deep coil stacking for MEMS application,” *IEEE Electron Device Lett.*, vol. 33, no. 3, pp. 432–434, 2012.

[83] T. Ebefors *et al.*, “The development and evaluation of RF TSV for 3D IPD applications,” *2013 IEEE Int. 3D Syst. Integr. Conf. 3DIC 2013*, 2013.

[84] F. Liang, S. Q. Zhao, A. Chen, and G. Wang, “Three-dimensional on-chip inductor design based on through-silicon vias,” *Proc. Int. Conf. ASIC*, no. 2, pp. 13–15, 2013.

[85] M. S. Mohamed Ali, B. Bycraft, A. Bsoul, and K. Takahata, “Radio-controlled microactuator based on shape-memory-alloy spiral-coil inductor,” *J. Microelectromechanical Syst.*, vol. 22, no. 2, pp. 331–338, 2013.

[86] A. Moazenzadeh *et al.*, “Wire bonded 3D coils render air core microtransformers competitive,” *J. Micromechanics Microengineering*, vol. 23, no. 11, 2013.

[87] Y. Gao *et al.*, “Significantly enhanced inductance and quality factor of GHz integrated magnetic solenoid inductors with FeGaB/Al2O3 Multilayer Films,” *IEEE Trans. Electron Devices*, vol. 61, no. 5, pp. 1470–1476, 2014.

[88] J. Kim, R. Shenoy, K. Y. Lai, and J. Kim, “High-Q 3D RF solenoid inductors in glass,” *Dig. Pap. - IEEE Radio Freq. Integr. Circuits Symp.*, pp. 199–200, 2014.

[89] F. Kahlouche *et al.*, “Fabrication and characterization of a planar interleaved micro-transformer with magnetic core,” *Microelectronics J.*, vol. 45, no. 7, pp. 893–897, 2014.

[90] J. M. Yook, D. S. Kim, and J. C. Kim, “High Power and High Q Spiral Inductors using TSV Processes.,” in *2013 Int. Conf. Solid State Devices Mater.*, 2015, pp. 126–127.

[91] B. M. F. Rahman, R. Divan, D. Rosenmann, T. Wang, Y. Peng, and G. Wang, “Application of sub-micrometer patterned permalloy thin film in tunable radio frequency inductors,” *J. Appl. Phys.*, vol. 117, no. 17, 2015.

[92] S. Y. Wu, C. Yang, W. Hsu, and L. Lin, “3D-printed microelectronics for integrated circuitry and passive wireless sensors,” *Microsystems Nanoeng.*, vol. 1, no. June, pp. 1–9, 2015.

[93] W. A. Vitale, M. Fernandez-Bolanos, A. Klumpp, J. Weber, P. Ramm, and A. M. Ionescu, “Ultra fine-pitch TSV technology for ultra-dense high-Q RF inductors,” *Dig. Tech. Pap. - Symp. VLSI Technol.*, vol. 2015-Augus, no. June, pp. T52–T53, 2015.

[94] T. Kifle, S. Chandra, and S. K. Koul, “A novel fabrication process for MEMS spiral inductors realized on oxide islands,” *Microsyst. Technol.*, vol. 21, no. 7, pp. 1415–1422, 2015.

[95] W. Huang, M. Li, S. Gong, and X. Li, “RFIC transformer with 12x size reduction and 15x performance enhancement by self-rolled-up membrane nanotechnology,” *ASME 2015 Int. Tech. Conf. Exhib. Packag. Integr. Electron. Photonic Microsystems*, vol. 3, pp. 2015–2018, 2015.

[96] M. S. K. F. J. K. S. T. T. Sato, “RF 2D micromachining - Fundamental study of high Q-factor RF spiral inductor using carbonyl-iron epoxy composite magnetic core.pdf,” *IEEJ Trans. Electr. Electron. Eng.*, vol. 11, 2016.

[97] M. A. Zhang, J. Shang, and B. Luo, “High Performance 3D Glass-Embedded Inductors Fabricated by a Glass Reflow Process,” *Proc. - Electron. Components Technol. Conf.*, vol. 2016-Augus, pp. 2588–2593, 2016.

[98] S. Hsieh, C. Chen, T. C. Lee, P. Lee, and C. Wang, “Fabrication Challenge, Device Characterization of High-Q 2D, 3D Passives Devices on Glass,” in *2016 IEEE Electr. Des. Adv. Packag. Syst.*, 2016, pp. 75–77.

[99] X. Li, Z. Ni, L. Gu, Z. Wu, and C. Yang, “Micromachined high-performance RF passives in CMOS substrate,” *J. Micromechanics Microengineering*, vol. 26, no. 11, 2016.

[100] H. Lee, B. S. Cook, K. P. Murali, M. Raj, and M. M. Tentzeris, “Inkjet Printed High-Q RF Inductors on Paper Substrate with Ferromagnetic Nanomaterial,” *IEEE Microw. Wirel. Components Lett.*, vol. 26, no. 6, pp. 419–421, 2016.

[101] P. A. Thadesar and M. S. Bakir, “Fabrication and Characterization of Polymer-Enhanced TSVs, Inductors, and Antennas for Mixed-Signal Silicon Interposer Platforms,” *IEEE Trans. Components, Packag. Manuf. Technol.*, vol. 6, no. 3, pp. 455–463, 2016.

[102] K. Ren *et al.*, “Process development of thick Si interposer for 2.5D integration of RF MEMS devices,” *2016 IEEE MTT-S Int. Microw. Work. Ser. Adv. Mater. Process. RF THz Appl. IMWS-AMP 2016 - Proceeding*, pp. 5–8, 2016.

[103] Y. C. Hsieh, Y. S. Chang, T. C. Lee, and C. C. Wang, “Characterization of through glass via (TGV) RF inductors,” *Proc. Tech. Pap. - Int. Microsystems, Packag. Assem. Circuits Technol. Conf. IMPACT*, no. 26, pp. 87–90, 2016.

[104] Y. Dhahri, S. Ghedira, R. Zrafi, and K. Besbes, “3D numerical analysis of integrated power inductor in silicon for DC/DC converters,” *16th Int. Conf. Sci. Tech. Autom. Control Comput. Eng. STA 2015*, no. 3, pp. 557–561, 2016.

[105] T. Zheng, G. Xu, and L. Luo, “High performance suspended spiral inductor and band-pass filter by wafer level packaging technology,” *Microsyst. Technol.*, vol. 23, no. 6, pp. 2107–2111, 2017.

[106] G. Wang, H. Liu, X. Li, H. Qiu, Y. Yang, and T. L. Ren, “Novel on-chip spiral inductors with back hollow structure,” *Mod. Phys. Lett. B*, vol. 31, no. 1, pp. 4–6, 2017.

[107] G. McKerricher, M. Vaseem, and A. Shamim, “Fully inkjet-printed microwave passive electronics,” *Microsystems Nanoeng.*, vol. 3, no. September 2016, 2017.

[108] A. El-Ghazaly, R. M. White, and S. X. Wang, “Gigahertz-band integrated magnetic inductors,” *IEEE Trans. Microw. Theory Tech.*, vol. 65, no. 12, pp. 4893–4900, 2017.

[109] J. Gu, Z. Guo, H. Yang, X. Li, and B. Row, “A micro-casting method for solenoid coil fabrication,” in *Transducers 2017*, 2017, pp. 1108–1111.

[110] A. L. C. Serrano *et al.*, “3D inductors with nanowire through substrate vias,” *IEEE MTT-S Int. Microw. Symp. Dig.*, pp. 1641–1644, 2017.

[111] M. Fernández-Bolaños *et al.*, “3D TSV based high frequency components for RF IC and RF MEMS applications,” *2016 IEEE Int. 3D Syst. Integr. Conf. 3DIC 2016*, pp. 8–11, 2017.

[112] S. Mondal, S. B. Cho, and B. C. Kim, “Modeling and crosstalk evaluation of 3-D TSV-based inductor with ground TSV shielding,” *IEEE Trans. Very Large Scale Integr. Syst.*, vol. 25, no. 1, pp. 308–318, 2017.

[113] V. F. G. Tseng, S. S. Bedair, and N. Lazarus, “3D electroplated inductors with thickness variation for improved broadband performance,” *J. Micromechanics Microengineering*, vol. 27, no. 1, 2017.

[114] W. Huang *et al.*, “CMOS-Compatible On-Chip Self-Rolled-Up Inductors for RF / mm-Wave Applications,” in *2017 IEEE MTT-S Int.*

Microw. Symp., 2017, pp. 1645–1648.

[115] A. Cayron, C. Viallon, O. Bushueva, A. Ghannam, and T. Parra, "High-Performance Compact 3-D Solenoids for RF Applications," *IEEE Microw. Wirel. Components Lett.*, vol. 28, no. 6, pp. 479–481, 2018.

[116] Y. Sun *et al.*, "Design, fabrication and characterization of a novel TSV interposer integrated inductor for RF applications," *Proc. - Electron. Components Technol. Conf.*, vol. 2018-May, pp. 2492–2497, 2018.

[117] J. M. Lopez-Villegas, N. Vidal, J. Sieiro, A. Salas, B. Medina, and F. M. Ramos, "Study of 3-D printed conical inductors for broadband RF applications," *IEEE Trans. Microw. Theory Tech.*, vol. 66, no. 8, pp. 3597–3602, 2018.

[118] M. Zia, H. Oh, and M. S. Bakir, "Post-CMOS fabrication technology enabling simultaneous fabrication of 3-D solenoidal micro-inductors and flexible I/Os," *IEEE Trans. Components, Packag. Manuf. Technol.*, vol. 8, no. 11, pp. 2039–2044, 2018.

[119] W. Huang *et al.*, "Three-dimensional radio-frequency transformers based on a self-rolled-up membrane platform," *Nat. Electron.*, vol. 1, no. 5, pp. 305–313, 2018.

[120] F. Wang, J. Huang, and N. Yu, "A low-pass filter made up of the cylindrical through-silicon-via," *Proc. - 2018 19th Int. Conf. Electron. Packag. Technol. ICEPT 2018*, pp. 257–259, 2018.

[121] W. Huang and X. Li, "Downscaling inductors with graphene," *Nat. Electron.*, vol. 1, no. 1, pp. 6–7, 2018.

[122] L. Wang *et al.*, "High-frequency Magnetic Properties of [Fe80Ni20-O/SiO2]n Multilayer Film Served as Magnetic Core of Solenoid Inductor," *IOP Conf. Ser. Mater. Sci. Eng.*, vol. 381, no. 1, 2018.

[123] X. Sun, G. Van Der Plas, and E. Beyne, "Improved staggered through silicon via inductors for RF and power applications," *Proc. - Electron. Components Technol. Conf.*, vol. 2018-May, pp. 1692–1697, 2018.

[124] J. Kang *et al.*, "On-chip intercalated-graphene inductors for next-generation radio frequency electronics," *Nat. Electron.*, vol. 1, no. 1, pp. 46–51, 2018.

[125] K. M. Subramani, G. T. Maganti, M. Gowri Jegatheesh, Y. A. Krishnaa, and K. Balamurugan, "High Performance Square Spiral Inductor for 65 nm CMOS V-band Low Noise Amplifier," *2018 Int. Conf. Adv. Comput. Commun. Informatics, ICACCI 2018*, no. Figure 1, pp. 1715–1719, 2018.

[126] X. Z. C. W. J. N. G. X. Ming, "Through glass via technology for ultra-high Q factor inductors," in *19th Int. Conf. Electron. Packag. Technol.*, 2018, pp. 215–218.

[127] A. Eblabla, X. Li, D. J. Wallis, I. Guiney, and K. Elgaid, "High-Performance MMIC Inductors for GaN-on-Low-Resistivity Silicon for Microwave Applications," *IEEE Microw. Wirel. Components Lett.*, vol. 28, no. 2, pp. 99–101, 2018.

[128] L. Hu, S. He, Y. Sun, and S. Ma, "Design and Process Technology for High Q Integrated Inductor on Interposer with TSV," *2018 Int. Conf. Microw. Millim. Wave Technol. ICMMT 2018 - Proc.*, pp. 1–3, 2018.

[129] Z. Ali, K. Ghosh, S. Aditya, and D. P. Poenar, "Fabrication of CNT-Based planar micro-coils on silicon substrate," *IEEE Trans. Nanotechnol.*, vol. 18, no. c, pp. 167–175, 2019.

[130] K. Hirmer, K. Hofmann, T. Casper, and S. Schöps, "3D field simulation model for bond wire on-chip inductors validated by measurements," *2019 Kleinheubach Conf. KHB 2019*, no. 2, pp. 5–8, 2019.

[131] J. P. Michel *et al.*, "Ultra-Low Profile Integrated Magnetic Inductors and Transformers for HF Applications," *IEEE Trans. Magn.*, vol. 55, no. 7, pp. 1–7, 2019.

[132] R. Bajwa and M. K. Yapici, "Intrinsic stress-induced bending as a platform technology for controlled self-assembly of high-Q on-chip RF inductors," *J. Micromechanics Microengineering*, vol. 29, no. 6, 2019.

[133] G. Pares, J. P. Michel, E. Deschaseaux, P. Ferris, A. Serhan, and A. Giry, "Highly compact RF transceiver module using high resistive silicon interposer with embedded inductors and heterogeneous dies integration," *Proc. - Electron. Components Technol. Conf.*, vol. 2019-May, pp. 1279–1286, 2019.

[134] C. W. Ha, P. Prabhakaran, and Y. Son, "3D-printed polymer/metal hybrid microstructures with ultraprecision for 3D microcoils," *3D Print. Addit. Manuf.*, vol. 6, no. 3, pp. 165–170, 2019.

[135] S. H. Li *et al.*, "Fully Symmetric 3-D Transformers with Through-Silicon via IPD Technology for RF Applications," *IEEE Trans. Components, Packag. Manuf. Technol.*, vol. 9, no. 11, pp. 2143–2151, 2019.

[136] X. Yin, Z. Zhu, Y. Liu, Q. Lu, X. Liu, and Y. Yang, "Ultra-Compact TSV-Based L-C Low-Pass Filter with Stopband Up to 40 GHz for Microwave Application," *IEEE Trans. Microw. Theory Tech.*, vol. 67, no. 2, pp. 738–745, 2019.

[137] Z. Zhang *et al.*, "Design and Evaluation of a Novel and Ultra-Compact Fully-TGV-based Self-Shielding Bandpass Filter for 5G Applications," *2019 Int. 3D Syst. Integr. Conf.*, pp. 1–4, 2020.

[138] F. Wang, V. F. Pavlidis, and N. Yu, "Miniaturized SIW Bandpass Filter Based on TSV Technology for THz Applications," *IEEE Trans. Terahertz Sci. Technol.*, no. c, pp. 2019–2021, 2020.

[139] W. J. Freitas, M. H. O. Piazzetta, L. T. Manera, and Â. L. Gobbi, "Fabrication process of integrated inductors on flexible substrate for radio frequency and microwave applications," *J. Vac. Sci. Technol. B*, vol. 38, no. 2, p. 023204, 2020.

[140] C. Prawoto, Z. Ma, Y. Xiao, S. Raju, C. Zhou, and M. Chan, "High Frequency Monolithic Inductor with Air-Gaps," in *2020 IEEE Electron Devices Technol. Manuf. Conf. Proc. Tech. Pap.*, 2020, vol. 1, no. 1, pp. 1–4.

[141] F. Khan and M. I. Younis, "Investigation of on-chip integrated inductors fabricated in SOI-MUMPs for RF MEMS ICs," *Analog Integr. Circuits Signal Process.*, vol. 102, no. 3, pp. 585–591, 2020.

[142] D. Dinulovic, M. Shousha, and M. Haug, "Microtransformer on silicon with CoFeB magnetic core for high-frequency signal applications," *AIP Adv.*, vol. 10, no. 1, 2020.

[143] H. Chen *et al.*, “Integrated Tunable Magnetoelectric RF Inductors,” *IEEE Trans. Microw. Theory Tech.*, vol. 68, no. 3, pp. 951–963, 2020.

[144] M. Mino, T. Yachi, A. Tago, K. Yanagisawa, and K. Sakakibara, “A New Planar Microtransformer for Use in Micro-Switching Converters,” *IEEE Trans. Magn.*, vol. 28, no. 4, pp. 1969–1973, 1992.

[145] C. R. Sullivan and S. R. Sander, “Microfabrication of transformers and inductors for high frequency power conversion,” *PESC Rec. - IEEE Annu. Power Electron. Spec. Conf.*, pp. 33–41, 1993.

[146] T. Sato, H. Tomita, A. Sawabe, T. Inoue, T. Mizoguchi, and M. Sahashi, “A Magnetic Thin Film Inductor and its Application to a MHz Switching dc-dc Converter,” *IEEE Trans. Magn.*, vol. 30, no. 2, pp. 217–223, 1994.

[147] Y. J. Kim and M. G. Allen, “A Fully Integrated Planar Toroidal Inductor With A Micromachined Nickel-Iron Magnetic Bar,” *IEEE Trans. Components Packag. Manuf. Technol. Part A*, vol. 17, no. 3, pp. 463–469, 1994.

[148] M. Mino, T. Yachi, K. Yanagisawa, A. Tago, and K. Tsukamoto, “Switching converter using thin film microtransformer with monolithically-integrated rectifier diodes,” *PESC Rec. - IEEE Annu. Power Electron. Spec. Conf.*, vol. 2, pp. 665–670, 1995.

[149] C. R. S. ; S. R. Sanders, “Microfabrication process for high-frequency power-conversion transformers,” in *Proc. PESC ’95 - Power Electron. Spec. Conf.*, 1995.

[150] M. Mino, K. Tsukamoto, K. Yanagisawa, A. Tago, and T. Yachi, “A Compact Buck-Converter Using a Thin-Film Inductor,” in *Proc. Appl. Power Electron. Conf. APEC ’96*, 1996, pp. 422–426.

[151] C. H. Ahn and M. G. Allen, “A comparison of two micromachined inductors (bar- and meander-type) for fully integrated boost DC/DC power converters,” *IEEE Trans. Power Electron.*, vol. 11, no. 2, pp. 239–245, 1996.

[152] C. R. Sullivan, C. R. Sullivan, S. R, and S. R, “Design of Microfabricted Transformers and Inductors for High-frequency Power Conversion,” *IEEE Trans. Power Electron.*, vol. 11, no. 2, pp. 228–238, 1996.

[153] C. R. Sullivan and S. R. Sanders, “Design of microfabricated transformers and inductors for high-frequency power conversion,” *IEEE Trans. Power Electron.*, vol. 11, no. 2, pp. 228–238, 1996.

[154] C. R. Sullivan and S. R. Sanders, “Measured performance of a high-power-density microfabricated transformer in a DC-DC converter,” *PESC Rec. - IEEE Annu. Power Electron. Spec. Conf.*, vol. 1, pp. 287–294, 1996.

[155] T. Sato *et al.*, “5 MHz switching micro DC-DC converter using planar inductor,” *INTELEC, Int. Telecommun. Energy Conf.*, pp. 485–490, 1996.

[156] M. Xu, T. M. Liakopoulos, and C. H. Ahn, “A Microfabricated Transformer for High-Frequency Power or Signal Conversion,” *IEEE Trans. Magn.*, vol. 34, no. 4 PART 1, pp. 1369–1371, 1998.

[157] and T. M. T. Inoue, K. Nishijima, S. Yatabe, “The effect of magnetic thin film structure on the Inductance of a Planar Inductor,” *IEEE Trans. Magn.*, vol. 34, no. 4, pp. 1372–1374, 1998.

[158] S. Sugahara, M. Edo, T. Sato, and K. Yamasawa, “Optimum chip size of a thin film reactor for a high-efficiency operation of a micro DC-DC converter,” *PESC Rec. - IEEE Annu. Power Electron. Spec. Conf.*, vol. 2, pp. 1499–1503, 1998.

[159] S. Iyengar, T. M. Liakopoulos, and C. H. Ahn, “A DC/DC boost converter toward fully on-chip integration using new micromachined planar inductors,” *PESC Rec. - IEEE Annu. Power Electron. Spec. Conf.*, vol. 1, pp. 72–76, 1999.

[160] G. J. Mehas, K. D. Coonley, and C. R. Sullivan, “Design of microfabricated inductors for microprocessor power delivery,” *Conf. Proc. - IEEE Appl. Power Electron. Conf. Expo. - APEC*, vol. 2, pp. 1181–1187, 1999.

[161] L. Daniel, C. R. Sullivan, and S. R. Sanders, “Design of microfabricated inductors,” *IEEE Trans. Power Electron.*, vol. 14, no. 4, pp. 709–723, 1999.

[162] J. Y. Park, S. H. Han, and M. G. Allen, “Batch-fabricated microinductors with electroplated magnetically anisotropie and laminated alloy cores,” *IEEE Trans. Magn.*, vol. 35, no. 5 PART 3, pp. 4291–4300, 1999.

[163] J. Y. Park and M. G. Allen, “Integrated electroplated micromachined magnetic devices using low temperature fabrication processes,” *IEEE Trans. Electron. Packag. Manuf.*, vol. 23, no. 1, p. 3, 2000.

[164] H. Nakazawa, M. Edo, Y. Katayama, M. Gekinozu, S. Sugahara, and Z. Hayashi, “Micro-DC/DC converter that integrates planar inductor on power IC,” *Ieee Trans. Magn.*, vol. 36, no. 5, pp. 3518–3520, 2000.

[165] S. Prabhakaran, D. E. Kreider, Y. Lin, C. R. Sullivan, and C. G. Levey, “Fabrication of thin-film V-groove inductors using composite magnetic materials,” *IWIPP 2000 - Int. Work. Integr. Power Packag.*, pp. 102–105, 2000.

[166] Y. Katayama, S. Sugahara, H. Nakazawa, and M. Edo, “High-Power-Density MHz-Switching Monolithic DC-DC Converter with Thin-Film Inductor,” in *2000 IEEE 31st Annu. Power Electron. Spec. Conf.*, 2000, vol. 00, pp. 1–6.

[167] T. Sato, K. Yamasawa, H. Tomita, T. Inoue, and T. Mizoguchi, “FeCoBN Magnetic Thin Film Inductor for MHz Switching Micro DC-DC Converters,” *IEEJ Trans. Ind. Appl.*, vol. 121, no. 1, pp. 84–89, 2001.

[168] J. Y. Park and J. U. Bu, “Fully integrated spiral-type microtransformers on a silicon substrate,” *Japanese J. Appl. Physics, Part 1 Regul. Pap. Short Notes Rev. Pap.*, vol. 40, no. 6 A, pp. 4023–4026, 2001.

[169] T. Sato, H. Yokoyama, K. Yamasawa, K. Toya, S. Kobayashi, and T. Minamisawa, “Multilayered transformer utilizing Mn-Zn ferrite and its application to a forward-type DC–DC converter,” *Electr. Eng. Japan*, vol. 135, no. 4, 2001.

[170] S. G. Kim, E. J. Yun, J. Y. Kim, J. Kim, and K. L. Cho, “Microfabrication and characteristics of double-rectangular spiral type thin-film inductors with an upper NiFe magnetic core,” *J. Appl. Phys.*, vol. 90, no. 7, pp. 3533–3538, 2001.

[171] C. S. Kim, S. Bae, H. J. Kim, S. E. Nam, and H. J. Kim, “Fabrication of high frequency DC-DC converter using Ti/FeTaN film inductor,” *IEEE Trans. Magn.*, vol. 37, no. 4 I, pp. 2894–2896, 2001.

[172] M. Brunet, T. O'Donnell, J. O'Brien, P. McCloskey, and C. O'Mathuna, "Design study and fabrication techniques for high power density micro-transformers," *Conf. Proc. - IEEE Appl. Power Electron. Conf. Expo. - APEC*, vol. 2, pp. 1189–1195, 2001.

[173] A. W. Lotfi and M. A. Wilkowski, "Issues and Advances in High-Frequency Magnetics for Switching Power Supplies," *Proc. IEEE*, vol. 89, no. 6, pp. 833–845, 2001.

[174] J. M. Boggetto, Y. Lembeye, J. P. Ferrieux, and J. P. Keradec, "Copper losses in power integrated inductors on silicon," *Conf. Rec. - IAS Annu. Meet. (IEEE Ind. Appl. Soc.*, vol. 2, pp. 977–983, 2002.

[175] J. W. Park, F. Cros, and M. G. Allen, "A sacrificial layer approach to highly laminated magnetic cores," *Proc. IEEE Micro Electro Mech. Syst.*, pp. 380–383, 2002.

[176] and J. K. Il-Yong Park, Sang Gi Kim, Jin Gun Koo, Tae Moon Roh, Dae Woo Lee, Yil Suk Yang, "A Fully Integrated Thin-Film Inductor and Its Application to a DC-DC Converter," *ETRI J.*, vol. 25, no. 4, 2003.

[177] S. Prabhakaran, C. R. Sullivan, and K. Venkatachalam, "Measured electrical performance of V-groove inductors for microprocessor power delivery," *Intermag 2003 - Progr. 2003 IEEE Int. Magn. Conf.*, vol. 39, no. 5, pp. 3190–3192, 2003.

[178] S. Musunuri and P. L. Chapman, "Multi-Layer Spiral Inductor Design for Monolithic DC-DC Converters," *Conf. Rec. - IAS Annu. Meet. (IEEE Ind. Appl. Soc.*, vol. 2, no. November 2003, pp. 1270–1275, 2003.

[179] J. W. Park and M. G. Allen, "Ultralow-Profile Micromachined Power Inductors with Highly Laminated Ni/Fe Cores: Application to Low-Megahertz DC-DC Converters," *IEEE Trans. Magn.*, vol. 39, no. 5 II, pp. 3184–3186, 2003.

[180] Y. Fukuda, T. Inoue, T. Mizoguchi, S. Yatabe, and Y. Tachi, "Planar inductor with ferrite layers for DC-DC converter," *IEEE Trans. Magn.*, vol. 39, no. 4 II, pp. 2057–2061, 2003.

[181] E. J. Brandon, E. Wesseling, V. White, C. Ramsey, L. Del Castillo, and U. Lieneweg, "Fabrication and characterization of microinductors for distributed power converters," *IEEE Trans. Magn.*, vol. 39, no. 4 II, pp. 2049–2056, 2003.

[182] D. P. Arnold, I. Zana, F. Cros, and M. G. Allen, "Vertically laminated magnetic cores by electroplating Ni-Fe into micromachined Si," *IEEE Trans. Magn.*, vol. 40, no. 4 II, pp. 3060–3062, 2004.

[183] T. O. Donnell *et al.*, "Thin Film Micro-Transformers for Future Power Conversion," in *19th Annu. IEEE Appl. Power Electron. Conf. Expo.*, 2004, pp. 939–944.

[184] G. Troussier *et al.*, "Fabrication of integrated inductors on silicon for fully integrated DC-DC microconverters," *Micromach. Microfabr. Process Technol. IX*, vol. 5342, no. 1, p. 86, 2004.

[185] N. Wang, H. Hauser, T. O. Donnell, M. Brunet, P. Mccloskey, and S. C. O. Mathuna, "Modeling of High-Frequency Micro-Transformers," *IEEE Trans. Magn.*, vol. 40, no. 4, pp. 2014–2016, 2004.

[186] J. W. Park, F. Cros, and M. G. Allen, "Planar spiral inductors with multilayer micrometer-scale laminated cores for compact-packaging power converter applications," *IEEE Trans. Magn.*, vol. 40, no. 4 II, pp. 2020–2022, 2004.

[187] E. Martincic, E. Figueras, E. Cabruja, E. Dufour-Gergam, and M. Woytasik, "Magnetic micro-transformers realized with a flip-chip process," *J. Micromechanics Microengineering*, vol. 14, no. 9, 2004.

[188] J. Laur *et al.*, "Fabrication of an integrated spiral-type inductor dedicated to DC-DC micro converters," in *2005 Eur. Conf. Power Electron. Appl.*, 2005, pp. 1–6.

[189] I. Kowase, T. Sato, K. Yamasawa, and Y. Miura, "A Planar Inductor Using Mn – Zn Ferrite / Polyimide Composite Thick Film for Low-Voltage and Large-Current DC–DC Converter," *IEEE Trans. Magn.*, vol. 41, no. 10, pp. 3991–3993, 2005.

[190] B. Estibals *et al.*, "Micro inductors using low temperature fabrication processes for integrated DC-DC microconverters," *Conf. Rec. - IAS Annu. Meet. (IEEE Ind. Appl. Soc.*, vol. 3, pp. 2212–2215, 2005.

[191] S. Musunuri and P. L. Chapman, "Design of low power monolithic DC-DC buck converter with integrated inductor," *PESC Rec. - IEEE Annu. Power Electron. Spec. Conf.*, vol. 2005, pp. 1773–1779, 2005.

[192] Y. Sun, W. Li, and C. R. Sullivan, "Microfabricated V-Groove Power Inductors for DC-DC Converters," in *IEEE 36th Power Electron. Spec. Conf.*, 2005, no. June, pp. 1513–1519.

[193] X. Y. Gao *et al.*, "Fabrication of ultralow-profile micromachined inductor with magnetic core material," *IEEE Trans. Magn.*, vol. 41, no. 12, pp. 4397–4400, 2005.

[194] S. C. Ó. Mathúna, T. O'Donnell, N. Wang, and K. Rinne, "Magnetics on silicon: An enabling technology for power supply on chip," *IEEE Trans. Power Electron.*, vol. 20, no. 3, pp. 585–592, 2005.

[195] Y. K. Yoon and M. G. Allen, "Embedded conductor technology for micromachined RF elements," *J. Micromechanics Microengineering*, vol. 15, no. 6, pp. 1317–1326, 2005.

[196] N. Wang, T. O'Donnell, S. Roy, M. Brunet, P. McCloskey, and S. C. O'Mathuna, "High-frequency micro-machined power inductors," *J. Magn. Magn. Mater.*, vol. 290-291 PA, pp. 1347–1350, 2005.

[197] X. Y. Gao, Y. Cao, Y. Zhou, W. Ding, C. Lei, and J. A. Chen, "Fabrication of solenoid-type inductor with electroplated NiFe magnetic core," *J. Magn. Magn. Mater.*, vol. 305, no. 1, pp. 207–211, 2006.

[198] D. Flynn, R. S. Dhariwal, and M. P. Y. Desmulliez, "A design study of microscale magnetic components for operation in the MHz frequency range," *J. Micromechanics Microengineering*, vol. 16, no. 9, pp. 1811–1818, 2006.

[199] D. Flynnt, H. Lup, C. Bailey, and M. Desmulliezt, "Assessment of MicroInductors for DC-DC Converters," in *2006 Int. Conf. Electron. Mater. Packag.*, 2006.

[200] L. Hua, D. Flynn, C. Bailey, and M. Desmulliez, "An analysis of a microfabricated solenoid inductor," *ESTC 2006 - 1st Electron. Syst. Technol. Conf.*, vol. 1, no. June 2015, pp. 556–561, 2006.

[201] B. Orlando, B. Orlando, R. Hida, R. Cuchet, M. Audoin, and B. Viala, "Low-resistance integrated toroidal inductor for power management," *IEEE Trans. Magn.*, vol. 42, no. 10, pp. 3374–3376, 2006.

[202] C. Lei *et al.*, "Fabrication of a solenoid-type inductor with Fe-based soft magnetic core," *J. Magn. Magn. Mater.*, vol. 308, no. 2, pp. 284–288, 2007.

[203] P. Galle *et al.*, "Ultra-Compact Power Conversion Based on a CMOS-Compatible Microfabricated Power Inductor with Minimized Core Losses," in *2007 Electron. Components Technol. Conf.*, 2007, pp. 1889–1894.

[204] Z. J. Shen, J. Lu, X. Cheng, H. N. Jia, and X. G. Ng, "On-chip bondwire inductor with ferrite-epoxy coating: A cost-effective approach to realize power systems on chip," *PESC Rec. - IEEE Annu. Power Electron. Spec. Conf.*, pp. 1599–1604, 2007.

[205] N. Wang, T. O'Donnell, S. Roy, P. McCloskey, and C. O'Mathuna, "Micro-inductors integrated on silicon for power supply on chip," *J. Magn. Magn. Mater.*, vol. 316, no. 2 SPEC. ISS., 2007.

[206] M. Wang, I. Batarseh, K. D. T. Ngo, and H. Xie, "Design and fabrication of integrated power inductor based on silicon molding technology," *PESC Rec. - IEEE Annu. Power Electron. Spec. Conf.*, pp. 1612–1618, 2007.

[207] J. Lu, H. Jia, A. Arias, X. Gong, and Z. J. Shen, "On-Chip Bondwire Magnetics with Ferrite-Epoxy Glob Coating for Power Systems on Chip," *Int. J. Power Manag. Electron.*, vol. 2008, pp. 1–9, 2008.

[208] T. El Mastouli, J. P. Laur, J. L. Sanchez, M. Brunet, D. Bourrier, and M. Dilhan, "Micro-inductors integrated on silicon for DC-DC converters," *Micromach. Microfabr. Process Technol. XIII*, vol. 6882, no. February 2008, p. 68820A, 2008.

[209] Y. Zhou, Z. M. Zhou, Y. Cao, X. Y. Gao, and W. Ding, "Fabrication and performance of Fe-based magnetic thin film inductor for high-frequency application," *J. Magn. Magn. Mater.*, vol. 320, no. 20, pp. 963–966, 2008.

[210] M. W. and M. Steyaert, "A fully-integrated 0.18 μm CMOS DC-DC step-down converter, using a bondwire spiral inductor," in *IEEE 2008 Cust. Intergrated Circuits Conf. A*, 2008.

[211] T. O'Donnell *et al.*, "Microfabricated inductors for 20 MHz dc-dc converters," *Conf. Proc. - IEEE Appl. Power Electron. Conf. Expo. - APEC*, pp. 689–693, 2008.

[212] M. Wang, K. D. T. Ngo, and H. Xie, "SU-8 enhanced high power density MEMS inductors," *IECON Proc. (Industrial Electron. Conf.*, pp. 2672–2676, 2008.

[213] D. W. Lee, K. P. Hwang, and S. X. Wang, "Fabrication and analysis of high-performance integrated solenoid inductor with magnetic core," *IEEE Trans. Magn.*, vol. 44, no. 11 PART 2, pp. 4089–4095, 2008.

[214] N. Wang, T. O'Donnell, R. Meere, F. M. F. Rhen, S. Roy, and S. C. O'Mathuna, "Thin-film-integrated power inductor on Si and its performance in an 8-MHz buck converter," *IEEE Trans. Magn.*, vol. 44, no. 11 PART 2, pp. 4096–4099, 2008.

[215] C. R. Sullivan, "Integrating magnetics for on-chip power: Challenges and opportunities," *Proc. Cust. Integr. Circuits Conf.*, no. Cicc, pp. 291–298, 2009.

[216] C. Martin *et al.*, "Planar inductors for high frequency DC-DC converters using microwave magnetic material," *2009 IEEE Energy Convers. Congr. Expo. ECCE 2009*, vol. i, pp. 1890–1894, 2009.

[217] S. G. Kim *et al.*, "Monolithic double rectangular spiral thin-film inductors implemented with NiFe magnetic cores for on-chip dc-dc converter applications," *Thin Solid Films*, vol. 517, no. 14, pp. 4204–4206, 2009.

[218] S. Bae *et al.*, "High Q Ni-Zn-Cu Ferrite inductor for on-chip power module," *IEEE Trans. Magn.*, vol. 45, no. 10, pp. 4773–4776, 2009.

[219] R. Meere, T. O'Donnell, H. J. Bergveld, N. Wang, and S. C. O'Mathuna, "Analysis of microinductor performance in a 20-100 MHz DC/DC converter," *IEEE Trans. Power Electron.*, vol. 24, no. 9, pp. 2212–2218, 2009.

[220] D. Flynn and M. P. Y. Desmulliez, "Design, fabrication, and characterization of flip-chip bonded microinductors," *IEEE Trans. Magn.*, vol. 45, no. 8, pp. 3055–3063, 2009.

[221] R. Meere, T. O'Donnell, N. Wang, N. Achotte, S. Kulkarni, and S. C. O'Mathuna, "Size and performance tradeoffs in micro-inductors for high frequency DC-DC conversion," *IEEE Trans. Magn.*, vol. 45, no. 10, pp. 4234–4237, 2009.

[222] D. S. Gardner, G. Schrom, F. Paillet, B. Jamieson, T. Karnik, and S. Borkar, "Review of on-chip inductor structures with magnetic films," *IEEE Trans. Magn.*, vol. 45, no. 10, pp. 4760–4766, 2009.

[223] C. D. Meyer, S. S. Bedair, B. C. Morgan, and D. P. Arnold, "High-Inductance-Density, Air-Core, Power Inductors, and Transformers Designed for Operation at 100–500 MHz," *IEEE Trans. Magn.*, vol. 46, no. 6, pp. 2236–2239, 2010.

[224] T. O'Donnell *et al.*, "Electrodeposited anisotropic NiFe 45/55 thin films for high-frequency micro-inductor applications," *J. Magn. Magn. Mater.*, vol. 322, no. 9–12, pp. 1690–1693, 2010.

[225] J. J. Lee *et al.*, "High-quality factor Ni-Zn ferrite planar inductor," *IEEE Trans. Magn.*, vol. 46, no. 6, pp. 2417–2420, 2010.

[226] N. W. ; J. H. ; R. F. ; K. M. ; T. O. ; K. Rodgers, "Integrated magnetics on silicon for power supply in package (PSiP) and power supply on chip (PwrSoC)," in *3rd Electron. Syst. Integr. Technol. Conf. ESTC*, 2010, pp. 0–5.

[227] M. Wang, J. Li, K. D. T. Ngo, and H. Xie, "A novel integrated power inductor in silicon substrate for ultra-compact power supplies," *Conf. Proc. - IEEE Appl. Power Electron. Conf. Expo. - APEC*, no. c, pp. 2036–2041, 2010.

[228] H. Jia, J. Lu, X. Wang, K. Padmanabhan, and Z. J. Shen, "Integration of a monolithic buck converter power IC and bondwire inductors with ferrite epoxy glob cores," *IEEE Trans. Power Electron.*, vol. 26, no. 6, pp. 1627–1630, 2011.

[229] M. Wang, J. Li, K. D. T. Ngo, and H. Xie, "A surface-mountable microfabricated power inductor in silicon for ultracompact power supplies," *IEEE Trans. Power Electron.*, vol. 26, no. 5, pp. 1310–1315, 2011.

[230] R. Wu and J. K. O. Sin, "A novel silicon-embedded coreless inductor for high-frequency power management applications," *IEEE*

Electron Device Lett., vol. 32, no. 1, pp. 60–62, 2011.

[231] M. Wang, J. Li, K. Ngo, and H. Xie, "Silicon molding techniques for integrated power MEMS inductors," *Sensors Actuators, A Phys.*, vol. 166, no. 1, pp. 157–163, 2011.

[232] J. Lee *et al.*, "Integrated ferrite film inductor for power system-on-chip (PowerSoC) smart phone applications," *IEEE Trans. Magn.*, vol. 47, no. 2 PART 1, pp. 304–307, 2011.

[233] L. Xue *et al.*, "Towards miniature step-up power converters for mobile microsystems," *Conf. Proc. - IEEE Appl. Power Electron. Conf. Expo. - APEC*, pp. 1451–1455, 2011.

[234] H. Ito *et al.*, "Fabrication of planar power inductor for embedded passives in LSI package for hundreds megahertz switching DC-DC buck converter," *IEEE Trans. Magn.*, vol. 47, no. 10, pp. 3204–3207, 2011.

[235] T. Rabia, Melati ; Azzedine, Hamid; Lebey, "Modeling and dimensioning of a planar inductor for a monolithic integration," in *2011 Asia-Pacific Power Energy Eng. Conf.*, 2011.

[236] R. Meere, N. Wang, T. O'Donnell, S. Kulkarni, S. Roy, and S. C. O'Mathuna, "Magnetic-core and air-core inductors on silicon: A performance comparison up to 100 MHz," *IEEE Trans. Magn.*, vol. 47, no. 10, pp. 4429–4432, 2011.

[237] Q. Li, "A fully-integrated buck converter design and implementation for on-chip power supplies," *J. Comput.*, vol. 7, no. 5, pp. 1270–1277, 2012.

[238] J. Qiu and C. R. Sullivan, "Design and fabrication of VHF tapped power inductors using nanogranular magnetic films," *IEEE Trans. Power Electron.*, vol. 27, no. 12, pp. 4965–4975, 2012.

[239] J. Qiu and C. R. Sullivan, "Radial-anisotropy thin-film magnetic material for high-power-density toroidal inductors," *2012 7th Int. Conf. Integr. Power Electron. Syst. CIPS 2012*, pp. 491–497, 2012.

[240] J. Li, K. D. T. Ngo, G. Q. Lu, and H. Xie, "Wafer-level fabrication of high-power-density MEMS passives based on silicon molding technique," *2012 7th Int. Conf. Integr. Power Electron. Syst. CIPS 2012*, no. February 2017, 2012.

[241] X. Yu, M. Araghchini, F. Herrault, J. K. Kim, J. H. Lang, and M. G. Allen, "Fabrication, modeling, and Performance Analysis of Silicon-Embedded 3-D Toroidal Inductors," *Proc. IEEE PowerMEMS*, pp. 3–6, 2012.

[242] X. Fang, R. Wu, L. Peng, and J. K. O. Sin, "A new embedded inductor for ZVS DC-DC converter applications," *Proc. Int. Symp. Power Semicond. Devices ICs*, no. June, pp. 53–56, 2012.

[243] R. Wu and J. K. O. Sin, "High-efficiency silicon-embedded coreless coupled inductors for power supply on chip applications," *IEEE Trans. Power Electron.*, vol. 27, no. 11, pp. 4781–4787, 2012.

[244] X. Yu, M. Kim, F. Herrault, C. H. Ji, J. Kim, and M. G. Allen, "Silicon-embedded 3D toroidal air-core inductor with through-wafer interconnect for on-chip integration," *Proc. IEEE Int. Conf. Micro Electro Mech. Syst.*, no. February, pp. 325–328, 2012.

[245] A. Kriga *et al.*, "Frequency characterization of thin soft magnetic material layers used in spiral inductors," *J. Magn. Magn. Mater.*, vol. 324, no. 14, pp. 2227–2232, 2012.

[246] D. V. Harburg, X. Yu, F. Herrault, C. G. Levey, M. G. Allen, and C. R. Sullivan, "Micro-fabricated thin-film inductors for on-chip power conversion," *2012 7th Int. Conf. Integr. Power Electron. Syst. CIPS 2012*, vol. 9, pp. 2–7, 2012.

[247] D. V. Harburg, J. Qiu, and C. R. Sullivan, "An improved AC loss model for the optimization of planar-coil inductors," *2012 IEEE 13th Work. Control Model. Power Electron. COMPEL 2012*, pp. 1–7, 2012.

[248] X. Yu, M. Kim, F. Herrault, C. H. Ji, J. Kim, and M. G. Allen, "Silicon-embedding approaches to 3-D toroidal inductor fabrication," *J. Microelectromechanical Syst.*, vol. 22, no. 3, pp. 580–588, 2013.

[249] D. V. Harburg, G. R. Khan, F. Herrault, J. Kim, C. G. Levey, and C. R. Sullivan, "On-chip RF power inductors with nanogranular magnetic cores using prism-assisted UV-LED lithography," *2013 Transducers Eurosensors XXVII 17th Int. Conf. Solid-State Sensors, Actuators Microsystems, TRANSDUCERS EUROSENSORS 2013*, no. June, pp. 701–704, 2013.

[250] L. Peng *et al.*, "A novel 3D TSV transformer technology for digital isolator gate driver applications," *Proc. Int. Symp. Power Semicond. Devices ICs*, pp. 69–72, 2013.

[251] R. Wu, J. K. O. Sin, and C. P. Yue, "High-Q Backside silicon-embedded inductor for power applications in μh and MHz range," *IEEE Trans. Electron Devices*, vol. 60, no. 1, pp. 339–345, 2013.

[252] B. Jamieson, J. F. Godsell, N. Wang, and S. Roy, "Device geometry effects in an integrated power microinductor with a Ni 45Fe55 enhancement layer," *IEEE Trans. Magn.*, vol. 49, no. 2, pp. 869–873, 2013.

[253] N. Wang *et al.*, "High frequency DC-DC converter with co-packaged planar inductor and power IC," *Proc. - Electron. Components Technol. Conf.*, pp. 1946–1952, 2013.

[254] Y. Sugawa, K. Ishidate, M. Sonehara, and T. Sato, "Carbonyl-iron/epoxy composite magnetic core for planar power inductor used in package-level power grid," *IEEE Trans. Magn.*, vol. 49, no. 7, pp. 4172–4175, 2013.

[255] N. Sturcken *et al.*, "A 2.5D integrated voltage regulator using coupled-magnetic-core inductors on silicon interposer," *IEEE J. Solid-State Circuits*, vol. 48, no. 1, pp. 244–254, 2013.

[256] D. Yao, C. G. Levey, R. Tian, and C. R. Sullivan, "Microfabricated V-groove power inductors using multilayer Co-Zr-O thin films for very-high-frequency DC-DC converters," *IEEE Trans. Power Electron.*, vol. 28, no. 9, pp. 4384–4394, 2013.

[257] J. Kim, F. Herrault, X. Yu, M. Kim, R. H. Shafer, and M. G. Allen, "Microfabrication of air core power inductors with metal-encapsulated polymer vias," *J. Micromechanics Microengineering*, vol. 23, no. 3, 2013.

[258] J. Kim, J. K. Kim, M. Kim, F. Herrault, and M. G. Allen, "Microfabrication of toroidal inductors integrated with nanolaminated ferromagnetic metallic cores," *J. Micromechanics Microengineering*, vol. 23, no. 11, 2013.

[259] M. Araghchini *et al.*, "Modeling and measured verification of stored energy and loss in MEMS toroidal inductors," *IEEE Trans. Ind. Appl.*, vol. 50, no. 3, pp. 2029–2038, 2014.

[260] X. Fang, R. Wu, L. Peng, and J. K. O. Sin, "A novel integrated power inductor with vertical laminated core for improved L/R ratios," *IEEE Electron Device Lett.*, vol. 35, no. 12, pp. 1287–1289, 2014.

[261] U. R. A. O. Tida, C. Zhuo, and Y. Shi, "Novel Through-Silicon-Via Inductor-Based On-Chip DC-DC Converter Designs in 3D ICs," *ACM J. Emerg. Technol. Comput. Syst.*, vol. 11, no. 2, 2014.

[262] Y. M. Nguyen *et al.*, "Soft ferrite cores characterization for integrated micro-inductors," *J. Micromechanics Microengineering*, vol. 24, no. 10, 2014.

[263] L. Peng *et al.*, "Optimization of Monolithic 3D TSV Transformers for High-Voltage Digital Isolators," *ECS J. Solid State Sci. Technol.*, vol. 3, no. 10, pp. Q207–Q211, 2014.

[264] R. Anthony, N. Wang, S. Kulkarni, and C. Mathuna, "Advances in planar coil processing for improved microinductor performance," *IEEE Trans. Magn.*, vol. 50, no. 11, pp. 18–21, 2014.

[265] J. Kim, M. Kim, F. Herrault, J. Y. Park, and M. G. Allen, "Electrodeposited nanolaminated conife cores for ultracompact DC-DC power conversion," *IEEE Trans. Power Electron.*, vol. 30, no. 9, pp. 5078–5087, 2015.

[266] E. Macrelli *et al.*, "Modeling, Design, and Fabrication of High-Inductance Bond Wire Microtransformers With Toroidal Ferrite Core," *IEEE Trans. Power Electron.*, vol. 30, no. 10, pp. 5724–5737, 2015.

[267] X. Yu and M. G. Allen, "Fabrication of multi-layer windings in silicon-embedded toroidal inductors," *2015 Transducers - 2015 18th Int. Conf. Solid-State Sensors, Actuators Microsystems*, pp. 610–613, 2015.

[268] A. E. Ostfeld, I. Deckman, A. M. Gaikwad, C. M. Lochner, and A. C. Arias, "Screen printed passive components for flexible power electronics," *Sci. Rep.*, vol. 5, pp. 1–11, 2015.

[269] K. Tien *et al.*, "An 82%-Efficient Multiphase Voltage-Regulator 3D Interposer with On-Chip Magnetic Inductors," *2015 Symp. VLSI Circuits*, pp. 192–193, 2015.

[270] U. R. Tida, S. Member, R. Yang, and C. Zhuo, "On the Efficacy of Through-Silicon-Via Inductors," *IEEE Trans. Very Large Scale Integr. Syst.*, vol. 23, no. 7, pp. 1322–1334, 2015.

[271] X. Fang, T. H. Mak, Y. Gao, K. M. Lau, P. K. T. Mok, and J. K. O. Sin, "A low substrate loss, monolithically integrated power inductor for compact LED drivers," *Proc. Int. Symp. Power Semicond. Devices ICs*, vol. 2015-June, pp. 53–56, 2015.

[272] C. Feeney, N. Wang, S. Cian O'Mathuna, and M. Duffy, "Design Procedure for Racetrack Microinductors on Silicon in Multi-MHz DC-DC Converters," *IEEE Trans. Power Electron.*, vol. 30, no. 12, pp. 6897–6905, 2015.

[273] M. Feeney, Ciaran; Wang, Ningning; Mathúna, Seán Cian; Duffy, "A 20-MHz 1.8-W DC–DC Converter With Parallel Microinductors and Improved Light-Load Efficienc," *IEEE Trans. Power Electron.*, vol. 30, no. 2, pp. 771–779, 2015.

[274] J. Kim, M. Kim, J. K. Kim, F. Herrault, and M. G. Allen, "Anisotropic nanolaminated CoNiFe cores integrated into microinductors for high-frequency DC-DC power conversion," *J. Phys. D. Appl. Phys.*, vol. 48, no. 46, p. 462001, 2015.

[275] N. Sturcken *et al.*, "Magnetic thin-film inductors for monolithic integration with CMOS," *Tech. Dig. - Int. Electron Devices Meet. IEDM*, vol. 2016-Febru, pp. 11.4.1-11.4.4, 2015.

[276] Z. Pavlovic, S. Kulkarni, N. Wang, and C. O. Mathuna, "High efficiency on-silicon coupled inductors using stacked copper windings," *2015 IEEE Energy Convers. Congr. Expo. ECCE 2015*, no. 318529, pp. 5302–5307, 2015.

[277] S. Mondal, J. Gamboa, and B. Kim, "Development of TSV-based Inductors in Power Electronics Packaging," in *16th Int. Conf. Electron. Packag. Technol.*, 2015, pp. 622–626.

[278] R. Anthony, E. Laforge, D. P. Casey, J. F. Rohan, and C. O'Mathuna, "High-aspect-ratio photoresist processing for fabrication of high resolution and thick micro-windings," *J. Micromechanics Microengineering*, vol. 26, no. 10, p. 105012, 2016.

[279] X. Fang, R. Wu, and J. K. O. Sin, "Analytical modeling of AC resistance in thick coil integrated spiral inductors," *IEEE Trans. Electron Devices*, vol. 63, no. 2, pp. 760–766, 2016.

[280] D. Mishra, P. M. Raj, and R. Tummala, "Design, fabrication and characterization of thin power inductors with multilayered ferromagnetic-polymer composite structures," *Microelectron. Eng.*, vol. 160, pp. 34–38, 2016.

[281] R. Anthony, N. Wang, D. P. Casey, C. Mathúna, and J. F. Rohan, "MEMS based fabrication of high-frequency integrated inductors on Ni-Cu-Zn ferrite substrates," *J. Magn. Magn. Mater.*, vol. 406, pp. 89–94, 2016.

[282] R. Anthony, C. O'Mathúna, and J. F. Rohan, "MEMS based electrochemical process for fabrication of laminated micro-inductors on silicon," *Microelectron. Eng.*, vol. 155, pp. 33–38, 2016.

[283] Y. He, L. Wang, Y. Wang, H. Zhang, Z. Zhong, and F. Bai, "On-chip solenoid power inductors with nanogranular magnetic cores," *Proc. - Int. Nanoelectron. Conf. INEC*, vol. 2016-Octob, no. 7, pp. 3–6, 2016.

[284] M. Lee, Y. Choi, and J. Kim, "A 500-MHz, 0.76-W/mm2 Power Density and 76.2% Power Efficiency, Fully Integrated Digital Buck Converter in 65-nm CMOS," *IEEE Trans. Ind. Appl.*, vol. 52, no. 4, pp. 3315–3323, 2016.

[285] D. Dinulovic, M. Shousha, M. Haug, A. Gerfer, M. Wens, and J. Thone, "On-chip high performance magnetics for point-of-load high-frequency DC-DC converters," *Conf. Proc. - IEEE Appl. Power Electron. Conf. Expo. - APEC*, vol. 2016-May, pp. 3097–3100, 2016.

[286] H. K. Krishnamurthy *et al.*, "A Digitally Controlled Fully Integrated Voltage Regulator With On-Die Solenoid Inductor With Planar Magnetic Core in 14-nm Tri-Gate CMOS," in *IEEE Int. Solid-State Circuits Conf.*, 2017, pp. 346–348.

[287] J. Li, V. F. G. Tseng, Z. Xiao, and H. Xie, "A High-Q In-Silicon Power Inductor Designed for Wafer-Level Integration of Compact DC-DC Converters," *IEEE Trans. Power Electron.*, vol. 32, no. 5, pp. 3858–3867, 2017.

[288] A. A. Muthukumaraswamy *et al.*, "Thin-Film Magnetic Inductor for Integrated Power Management," *Proc. - Electron. Components Technol. Conf.*, pp. 1485–1490, 2017.

[289] C. Femandez, Z. Pavlovic, S. Kulkami, P. McCloskey, and C. O'Mathuna, "High frequency, single/dual phases, large AC/DC signal power characterization for two phase on-silicon coupled inductors," *Conf. Proc. - IEEE Appl. Power Electron. Conf. Expo. - APEC*, pp. 2488–2493, 2017.

[290] Y. Ding *et al.*, "A power inductor integration technology using a silicon interposer for DC-DC converter applications," *Proc. Int. Symp. Power Semicond. Devices ICs*, vol. 2018-May, pp. 347–350, 2018.

[291] Z. A. Syed Mohammed, P. D. Puiu, and S. Aditya, "Fabrication of silicon-embedded low resistance high-aspect ratio planar copper microcoils," *J. Micro/Nanolithography, MEMS, MOEMS*, vol. 17, no. 01, p. 1, 2018.

[292] D. Dinulovic *et al.*, "High inductance thin-film transformer for high switching frequency," *Conf. Proc. - IEEE Appl. Power Electron. Conf. Expo. - APEC*, vol. 2018-March, pp. 560–564, 2018.

[293] B. Chen, U. Tida, C. Zhuo, and Y. Shi, "Modeling and optimization of magnetic core TSV-inductor for on-chip DC-DC converter," *IEEE/ACM Int. Conf. Comput. Des. Dig. Tech. Pap. ICCAD*, 2018.

[294] M. L. F. Bellaredj, C. A. Pardue, P. Kohl, and M. Swaminathan, "Fabrication of package embedded spiral inductors with two magnetic layers for flexible SIP point of load converters in Internet of Everything devices," *Microelectron. Eng.*, vol. 189, pp. 18–27, 2018.

[295] H. Li, J. Liu, T. Xu, J. Xia, X. Tan, and Z. Tao, "Fabrication and optimization of high aspect ratio through-silicon-vias electroplating for 3D inductor," *Micromachines*, vol. 9, no. 10, 2018.

[296] H. T. Le *et al.*, "Fabrication of 3D air-core MEMS inductors for very-high-frequency power conversions," *Microsystems Nanoeng.*, 2018.

[297] D. V. Harburg *et al.*, "Microfabricated Racetrack Inductors with Thin-Film Magnetic Cores for On-Chip Power Conversion," *IEEE J. Emerg. Sel. Top. Power Electron.*, vol. 6, no. 3, pp. 1280–1294, 2018.

[298] R. Wu, N. Liao, J. K. O. Sin, B. Bardet, J. Billoué, and G. Gautier, "A Silicon-Embedded Inductor Surrounded by Porous Silicon for Improved Quality Factor," *ECS J. Solid State Sci. Technol.*, vol. 7, no. 6, pp. Q112–Q115, 2018.

[299] H.-Y. Chan, M. Bachman, and G.-P. Li, "Interconnects and Interposer in Porous Anodic Aluminum Oxide: The Fabrication Technology and Process Integration," *ECS J. Solid State Sci. Technol.*, vol. 7, no. 8, pp. P385–P390, 2018.

[300] H. T. Le, Y. Nour, A. Han, F. Jensen, Z. Ouyang, and A. Knott, "Microfabricated Air-Core Toroidal Inductor in Very High-Frequency Power Converters," *IEEE J. Emerg. Sel. Top. Power Electron.*, vol. 6, no. 2, pp. 604–613, 2018.

[301] V. Lafage, Y. Beilliard, A. Sridhar, T. Brunschwiler, and D. Drouin, "Fabrication of 2D and 3D inductors for DC-DC converters integrated on glass interposer," *2018 Pan Pacific Microelectron. Symp. Pan Pacific 2018*, vol. 2018-Janua, pp. 1–8, 2018.

[302] A. Abdeldjebbar, A. Hamid, Y. Guettaf, and R. Melati, "Design of micro-transformer in monolithic technology for high frequencies fly-back type converters," *Prz. Elektrotechniczny*, vol. 94, no. 8, pp. 85–94, 2018.

[303] H. K. Krishnamurthy *et al.*, "A Digitally Controlled Fully Integrated Voltage Regulator With On-Die Solenoid Inductor With Planar Magnetic Core in 14-nm Tri-Gate CMOS," *IEEE J. Solid-State Circuits*, vol. 53, no. 1, pp. 8–19, 2018.

[304] H. T. Le *et al.*, "High-Q Three-Dimensional Microfabricated Magnetic-Core Toroidal Inductors for Power Supplies in Package," *IEEE Trans. Power Electron.*, vol. 34, no. 1, pp. 74–85, 2019.

[305] T. Fukuoka, Y. Karasawa, T. Akiyama, R. Oka, S. Ishida, and T. Shirasawa, "An 86% Efficiency, 20MHz, 3D-Integrated Buck Converter with Magnetic Core Inductor Embedded in Interposer Fabricated by Epoxy/Magnetic-Filler Composite Build-Up Sheet," in *2019 IEEE Appl. Power Electron. Conf. Expo.*, 2019, pp. 1561–1566.

[306] Y. C. Hsieh, S. Y. Lin, C. Y. Kung, P. N. Lee, and C. C. Wang, "Ultra low profile power inductor integrated in wafer level package," *2019 IEEE 21st Electron. Packag. Technol. Conf. EPTC 2019*, no. 26, pp. 175–178, 2019.

[307] M. L. F. Bellaredj, A. K. Davis, P. Kohl, and M. Swaminathan, "Magnetic Core Solenoid Power Inductors On Organic Substrate for System in Package Integrated High Frequency Voltage Regulators," *IEEE J. Emerg. Sel. Top. Power Electron.*, vol. 30332, no. c, pp. 1–1, 2019.

[308] P. Renz, M. Kaufmann, M. Lueders, and B. Wicht, "A Fully Integrated 85%-Peak-Efficiency Hybrid Multi- Ratio Resonant DC-DC Converter with 3.0-to-4.5V Input and 500µA-to-120mA Load Range," in *2019 IEEE Int. Solid- State Circuits Conf.* -, 2019, pp. 156–158.

[309] T. Xu, J. Sun, H. Wu, H. Li, H. Li, and Z. Tao, "3D MEMS In-Chip Solenoid Inductor with High Inductance Density for Power MEMS Device," *IEEE Electron Device Lett.*, vol. 40, no. 11, pp. 1816–1819, 2019.

[310] N. Xu, S. Member, and W. Ki, "Investigation of On-Chip Inductors for Fully Integrated DC-DC Converters," *IEEE APCCAS 2019*, no. c, pp. 2–5, 2019.

[311] Y. Ding, X. Fang, R. Wu, Q. Guo, and J. K. O. Sin, "A silicon molded metal transfer process for on-chip suspended power inductors," in *2019 20th Int. Conf. Solid-State Sensors, Actuators Microsystems Eurosensors XXXIII (TRANSDUCERS EUROSENSORS XXXIII)*, 2019, no. June, pp. 142–145.

[312] R. P. Singh, R. Salahuddin, L. Peng, L. Selvaraj, D. Disney, and M. A. Arasu, "Thin-Film Magnetic Inductors for High-Frequency Switching Regulator," *2019 Electron Devices Technol. Manuf. Conf. EDTM 2019*, pp. 398–400, 2019.

[313] T. Xu *et al.*, "A 3D MEMS In-Chip Solenoid Inductor of High Inductance Density for Future Power-MEMS Device," *2019 20th Int. Conf. Solid-State Sensors, Actuators Microsystems Eurosensors XXXIII, TRANSDUCERS 2019 EUROSENSORS XXXIII*, no. June, pp. 1459–1462, 2019.

[314] T. Akiyama *et al.*, "Integrated CMOS switch buck DC-DC converter fabricated in organic interposer with embedded magnetic core

inductor," *J. Magn. Soc. Japan*, vol. 43, no. 3, pp. 64–69, 2019.

[315] T. Sun *et al.*, "Substrate-embedded low-resistance solenoid inductors for integrated voltage regulators," *IEEE Trans. Components, Packag. Manuf. Technol.*, vol. 10, no. 1, pp. 134–141, 2020.

[316] Y. Huang, H. Li, J. Sun, Y. Zhai, H. Li, and T. Xu, "Powder filling and sintering of 3D in-chip solenoid coils with high aspect ratio structure," *Micromachines*, vol. 11, no. 3, pp. 1–10, 2020.

[317] Z. Pavlovic *et al.*, "Thin - film Magnetics - on - Silicon Integrated Transformer for Isolated Signal and Power Coupling Applications Device design Transformer design specifications," in *11th Int. Conf. Integr. Power Electron. Syst.*, 2020, pp. 264–268.

[318] B. Gimi, S. Eroglu, L. Leoni, T. A. Desai, R. L. Magin, and B. B. Roman, "NMR spiral surface microcoils: Applications," *Concepts Magn. Reson. Part B Magn. Reson. Eng.*, vol. 18, no. 1, pp. 1–8, 2003.

[319] R. R. A. Syms, M. M. Ahmad, I. R. Young, D. Gilderdale, D. J. Collins, and M. O. Leach, "Batch fabrication of micro-coils for MR spectroscopy on silicon," *Proc. IEEE Sensors*, vol. 2005, pp. 227–230, 2005.

[320] J. G. K. Mona J. K. Klein, T. Ono, M. Esashi, "RIE of solenoidal microcoil glass mould with integrated sample container for micro-MRI," in *IEEE 20th Int. Conf. Micro Electro Mech. Syst.*, 2007, no. January, pp. 345–348.

[321] M. J. K. Klein, T. Ono, M. Esashi, and J. G. Korvink, "Process for the fabrication of hollow core solenoidal microcoils in borosilicate glass," *J. Micromechanics Microengineering*, vol. 18, no. 7, 2008.

[322] R. Wu, S. Raju, M. Chan, J. K. O. Sin, and C. P. Yue, "Wireless power link design using silicon-embedded inductors for brain-machine interface," *2012 Int. Symp. VLSI Des. Autom. Test, VLSI-DAT 2012 - Proc. Tech. Pap.*, no. April, 2012.

[323] R. Wu, S. Raju, M. Chan, J. K. O. Sin, and C. P. Yue, "Silicon-embedded receiving coil for high-efficiency wireless power transfer to implantable biomedical ics," *IEEE Electron Device Lett.*, vol. 34, no. 1, pp. 9–11, 2013.

[324] M. B. Coskun, K. Thotahewa, Y. S. Ying, M. Yuce, A. Neild, and T. Alan, "Nanoscale displacement sensing using microfabricated variable-inductance planar coils," *Appl. Phys. Lett.*, vol. 103, no. 14, 2013.

[325] W. Wu *et al.*, "The design and fabrication of a low-field NMR probe based on a multilayer planar microcoil," *Microsyst. Technol.*, vol. 20, no. 3, pp. 419–425, 2014.

[326] R. M. Fratila, M. V. Gomez, S. Sýkora, and A. H. Velders, "Multinuclear nanoliter one-dimensional and two-dimensional NMR spectroscopy with a single non-resonant microcoil," *Nat. Commun.*, vol. 5, 2014.

[327] J. Olivo, S. Carrara, and G. De Micheli, "Micro-fabrication of high-thickness spiral inductors for the remote powering of implantable biosensors," *Microelectron. Eng.*, vol. 113, pp. 130–135, 2014.

[328] X. Sun, X. Peng, Y. Zheng, X. Li, and H. Zhang, "A 3-D stacked high-Q PI-based MEMS inductor for wireless power transmission system in bio-implanted applications," *J. Microelectromechanical Syst.*, vol. 23, no. 4, pp. 888–898, 2014.

[329] S. W. Lee, F. Fallegger, B. D. F. Casse, and S. I. Fried, "Implantable microcoils for intracortical magnetic stimulation," *Sci. Adv.*, vol. 2, no. 12, 2016.

[330] X. Sun, Z. Feng, S. Zhi, C. Lei, D. Zhang, and Y. Zhou, "An integrated microfluidic system using a micro-fluxgate and micro spiral coil for magnetic microbeads trapping and detecting," *Sci. Rep.*, vol. 7, no. 1, pp. 1–8, 2017.

[331] S. Cardoso *et al.*, "Challenges and trends in magnetic sensor integration with microfluidics for biomedical applications," *J. Phys. D. Appl. Phys.*, vol. 50, no. 21, 2017.

[332] R. Likhite, W. Luo, D. J. Young, and C. H. Mastrangelo, "Fabrication and inductive power transfer in wireless spherical pill microprobes," *TRANSDUCERS 2017 - 19th Int. Conf. Solid-State Sensors, Actuators Microsystems*, pp. 32–35, 2017.

[333] Y. Zhang and N. T. Nguyen, "Magnetic digital microfluidics - a review," *Lab Chip*, vol. 17, no. 6, pp. 994–1008, 2017.

[334] Z. Feng, S. Zhi, L. Guo, M. Wei, Y. Zhou, and C. Lei, "A novel integrated microfluidic platform based on micro-magnetic sensor for magnetic bead manipulation and detection," *Microfluid. Nanofluidics*, vol. 22, no. 8, pp. 1–9, 2018.

[335] M. E. Rizou and T. Prodromakis, "Magnetic stimulation in the microscale: The development of a 6 × 6 array of micro-coils for stimulation of excitable cells in vitro," *Biomed. Phys. Eng. Express*, vol. 4, no. 2, 2018.

[336] M. E. Rizou and T. Prodromakis, "Electrothermal deterioration factors in gold planar inductors designed for microscale bio-applications," *Microelectron. Eng.*, vol. 197, pp. 61–66, 2018.

[337] R. Shadid and S. Noghanian, "A Literature Survey on Wireless Power Transfer for Biomedical Devices," *Int. J. Antennas Propag.*, vol. 2018, 2018.

[338] A. Dupré, K. M. Lei, P. I. Mak, R. P. Martins, and W. K. Peng, "Micro- and nanofabrication NMR technologies for point-of-care medical applications – A review," *Microelectron. Eng.*, vol. 209, no. October 2018, pp. 66–74, 2019.

[339] S. W. Lee, K. Thyagarajan, and S. I. Fried, "Micro-Coil Design Influences the Spatial Extent of Responses to Intracortical Magnetic Stimulation," *IEEE Trans. Biomed. Eng.*, vol. 66, no. 6, pp. 1680–1694, 2019.

[340] N. Ben Mansour, C. Dridi, N. Yaakoubi, and L. Fakri-Bouchet, "NMR Implantable Microcoil FEM Based Comparative Study for Numerical Brain Model Application," *IEEE Int. Conf. Des. Test Integr. Micro Nano-Systems, DTS 2019*, pp. 1–6, 2019.

[341] S. Zhi, X. Sun, Z. Feng, C. Lei, and Y. Zhou, "An innovative micro magnetic separator based on 3D micro-copper-coil exciting soft magnetic tips and FeNi wires for bio-target sorting," *Microfluid. Nanofluidics*, vol. 23, no. 3, p. 0, 2019.

[342] O. Lefebvre *et al.*, "Reusable embedded microcoils for magnetic nano-beads trapping in microfluidics: Magnetic simulation and experiments," *Micromachines*, vol. 11, no. 3, pp. 1–17, 2020.

[343] J. Handwerker *et al.*, "A CMOS NMR needle for probing brain physiology with high spatial and temporal resolution," *Nat. Methods*, vol. 17, no. 1, pp. 64–67, 2020.

[344] S. Kawahito, Y. Sasaki, H. Sato, T. Nakamura, and Y. Tadokoro, "A fluxgate magnetic sensor with micro-solenoids and electroplated permalloy cores," *Sensors Actuators A. Phys.*, vol. 43, no. 1–3, pp. 128–134, 1994.

[345] M. Woytasik *et al.*, "Two- and three-dimensional microcoil fabrication process for three-axis magnetic sensors on flexible substrates," *Sensors Actuators, A Phys.*, vol. 132, no. 1 SPEC. ISS., pp. 2–7, 2006.

[346] M. S. M. Ali, A. AbuZaiter, C. Schlosser, B. Bycraft, and K. Takahata, "Wireless displacement sensing of micromachined spiral-coil actuator using resonant frequency tracking," *Sensors (Switzerland)*, vol. 14, no. 7, pp. 12399–12409, 2014.

[347] R. Wu, N. Liao, X. Fang, and J. K. O. Sin, "A silicon-embedded transformer for high-efficiency, high-isolation, and low-frequency on-chip power transfer," *IEEE Trans. Electron Devices*, vol. 62, no. 1, pp. 220–223, 2015.

[348] P. G. G. Meysam Zargham, "Fully integrated on-chip coil in 0.13 μm CMOS for wireless power transfer through biological media," *IEEE Trans. Biomed. Circuits Syst.*, vol. 2, pp. 259–71, 2015.

[349] R. Wu, N. Liao, X. Fang, and J. K. O. Sin, "A novel 3D transformer for ultra-compact signal isolation," *Proc. Int. Symp. Power Semicond. Devices ICs*, vol. 2015-June, pp. 297–300, 2015.

[350] J. Kim *et al.*, "Miniaturized Flexible Electronic Systems with Wireless Power and Near-Field Communication Capabilities," *Adv. Funct. Mater.*, vol. 25, no. 30, pp. 4761–4767, 2015.

[351] B. O. Jun *et al.*, "Wireless thin film transistor based on micro magnetic induction coupling antenna," *Sci. Rep.*, vol. 5, pp. 1–9, 2015.

[352] X. Yu *et al.*, "Ultra-Small, High-Frequency, and Substrate-Immune Microtube Inductors Transformed from 2D to 3D," *Sci. Rep.*, vol. 5, pp. 1–6, 2015.

[353] R. Wu, N. Liao, and X. Fang, "A Novel Double-Side Silicon-Embedded Transformer for 10-MHz, 1-kV-Isolation, Compact Power Transfer Applications," *IEEE Trans. Electron Devices*, pp. 1–4, 2016.

[354] A. Baldwin, L. Yu, M. Pratt, K. Scholten, and E. Meng, "Passive, wireless transduction of electrochemical impedance across thin-film microfabricated coils using reflected impedance," *Biomed. Microdevices*, vol. 19, no. 4, 2017.

[355] R. Wu, N. Liao, X. Fang, J. Cai, Q. Wang, and J. K. O. Sin, "A 3D Assembled Silicon-Embedded Transformer for 10-MHz, Ultra-High-Isolation, Compact Chip-to-Chip Power Transfer," *IEEE Electron Device Lett.*, vol. 38, no. 3, pp. 356–358, 2017.

[356] C. A. Pardue *et al.*, "Design and Characterization of Inductors for Self-Powered IoT Edge Devices," *IEEE Trans. Components, Packag. Manuf. Technol.*, vol. 8, no. 7, pp. 1263–1271, 2018.

[357] and O. G. S. Dmitriy D. Karnaushenko, Daniil Karnaushenko, Hans-Joachim Grafe, Vladislav Kataev, Bernd Büchner, "Rolled-Up Self-Assembly of Compact Magnetic Inductors, Transformers, and Resonators," *Adv. Electron. Mater.*, vol. 4, no. 1800298, 2018.

[358] Q. Tang, L. Wu, X. Chen, and D. Peng, "An Inductive Linear Displacement Sensor Based on Planar Coils," *IEEE Sens. J.*, vol. 18, no. 13, pp. 5256–5264, 2018.

[359] C. A. Pardue, M. L. F. Bellaredj, H. M. Torun, M. Swaminathan, P. Kohl, and A. K. Davis, "Rf wireless power transfer using integrated inductor," *IEEE Trans. Components, Packag. Manuf. Technol.*, vol. 9, no. 5, pp. 913–920, 2019.

[360] K. S, R. Komaragiri, and S. K.J, "Fabrication, characterization, and modelling of a novel via-less single metal level magnetic microcoil sensor for biosensing applications," *Sensors Actuators, A Phys.*, vol. 290, pp. 190–197, 2019.

[361] T. Hara and M. Yamaguchi, "Spray-coated magnetic powder composite planar inductor for compact IoT sensor node," *AIP Adv.*, vol. 10, no. 1, 2020.

[362] S. Lin *et al.*, "Usage of the impedance effect of a spiral dual-coil for fingerprint sensor application," *Sensors Actuators, A Phys.*, vol. 303, no. xxxx, p. 111740, 2020.
