# Supplementary material for: MEMS inductor fabrication and emerging applications in power electronics and neurotechnologies
Source: Microsyst Nanoeng. 2021 Aug 11;7:59. doi: 10.1038/s41378-021-00275-w (PMC8433479; doi:10.1038/s41378-021-00275-w)
Supplement: Supplementary file 2 — Table: MEMS inductor categories and their characteristics [file 41378_2021_275_MOESM2_ESM.pdf]

Table 1 : MEMS inductor categories and their characteristics

Characteristics \ Category	2D on-substrate Inductors*	3D on-substrate inductors	2D in-substrate inductors	3D in-substrate inductors
**Geometry**	2D spiral, racetrack. Planar- and suspended windings.	Solenoid, toroid. On-substrate 3D windings.	2D spiral. Si-embedded windings with TSVs and front/backside redistribution layers (RDL).	Si-embedded 2D solenoid and toroid. Si-embedded windings with TSVs and front/backside redistribution layers (RDL).
**Applications**	RF, wireless, wireless power transfer, magnetic sensors	RF, power electronics	RF, power electronics, wireless power transfer	Power electronics
**Fabrication technology**	Simlpe 2D surface micromachining.<br>CMOS-compatible. Magnetic core integration.	Advanced 3D surface micromachining, wire-bonding, self-assembly.<br>CMOS-compatible.	Advanced 2D TSV-based processes, silicon-mold process.<br>CMOS-compatible.	Highly advanced 3D TSV-based processes.<br>CMOS-compatible. Magnetic core integration.
**Merits**	Compact size. Fabrication ease.	High inductance density.	Low profile. Efficient thermal dissipation. 3D integration with TSVs.	Low profile. Higher inductance density. Efficient thermal dissipation. 3D integration with TSVs.
**Electromagnetic properties**	Out-of-plane magnetic field. High substrate losses.	In-plane magnetic field. Low substrate losses. High turn-to-turn parasitic.	Out-of-plane magnetic field. High substrate losses.	In-plane magnetic field. High substrate losses. High turn-to-turn parasitic.

**2D inductors include inductors with spiral winding shapes such as circular and rectangular spiral inductors, and racetrack inductors. 3D inductors include inductors that have at least three levels of windings such as solenoid and toroidal inductors.*
